# Precious Metal‐Free Artificial Leaf for Photosynthesis of Hydrogen Peroxide from Water

**DOI:** 10.1002/cssc.202501055

**Published:** 2025-09-17

**Authors:** Thomas Freese, Alexandra Matei, Maria B. Brands, Marina Karsakova, Diego A. Acevedo–Guzmán, Dominic Gerlach, Petra Rudolf, Joost N. H. Reek, Ben L. Feringa

**Affiliations:** ^1^ Stratingh Institute for Chemistry University of Groningen Nijenborgh 4 9747 AG Groningen The Netherlands; ^2^ van ’t Hoff Institute for Molecular Sciences University of Amsterdam Science Park 904 1098 XH Amsterdam The Netherlands; ^3^ Zernike Institute for Advanced Materials University of Groningen Nijenborgh 4 9747AG Groningen The Netherlands

**Keywords:** electrochemistry, hydrogen peroxide, photocatalysis, photosynthesis, solar fuels

## Abstract

Hydrogen peroxide (H_2_O_2_) is recognized as an environmentally friendly oxidant with a wide range of applications, as well as a promising future energy carrier compared to hydrogen. Light driven and electrochemical production of H_2_O_2_ have gained significant interest as promising alternatives to the energy‐intensive anthraquinone process. The two main approaches for the (photo)electrochemical production of H_2_O_2_ are the water oxidation reaction (WOR) and the oxygen reduction reaction (ORR). Considering the scarcity of noble metals, it is critical to develop successful high‐performing electrocatalysts based on earth‐abundant sources, thus adhering to principles of Green and Sustainable Chemistry. Herein, the use of the recently developed FeO_x_ nanoparticles (NP) catalyst as a photocathode, circumventing catalyst deactivation and oxidation, is reported. The FeO_x_ NP photocathode exhibited an increased catalytic current by 41% under illumination, demonstrating the advantage of a photoelectrochemical (PEC) setup. Combining the FeO_x_ NP photocathode for ORR with a Ti‐doped α‐Fe_2_O_3_ photoanode for WOR, robust PEC performance is successfully achieved in bias‐free conditions. The structural integrity of the FeO_x_ NP photocathode is preserved without degradation or oxidation for extended periods of irradiation of up to 10 h testimony of a benign and robust process.

## Graphical abstract

This work explores the photoelectrochemical production of H_2_O_2_ via oxygen reduction reaction and water oxidation reaction. It emphasizes the need for earth‐abundant photoelectrocatalysts and presents FeO_x_ nanoparticles as stable, efficient photocathode material. Combined with a Ti‐doped α‐Fe_2_O_3_ photoanode, the system showed robust, bias‐free operation and long‐term stability.

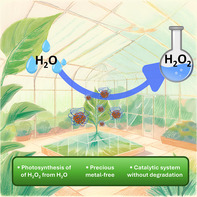

## 1 Introduction

Hydrogen peroxide (H_2_O_2_) is an environmentally friendly oxidant and is extensively used, among others in chemical synthesis, sterilization, wastewater treatment, and the paper industry.^[^ ^1^, ^2^, ^3^, ^4^ ^]^ However, its large–scale industrial production primarily relies on the anthraquinone process, which requires expensive palladium catalysts and involves a complex separation process to remove significant amounts of organic waste.^[^ ^5^, ^6^, ^7^ ^]^

Consequently, developing efficient, economical and sustainable technologies for hydrogen peroxide production is highly desirable.^[^ ^8^, ^9^, ^10^ ^]^ Electrochemical water splitting is anticipated to provide an eco–friendly method for harvesting and storing energy from renewable sources, such as solar energy, in the form of “green” hydrogen (H_2_) as well as H_2_O_2_.^[^ ^11^, ^12^, ^13^, ^14^, ^15^ ^]^ Like hydrogen, H_2_O_2_ also has been recognized as an energy carrier in fuel cells, providing several advantages over hydrogen (H_2_) such as being a transportable liquid with full solubility in water and thereby achieving safer operation, storage, and transportation.^[^ ^16^, ^17^, ^18^, ^19^, ^20^, ^21^ ^]^

Over the past few decades, there has been a growing interest in light‐driven water splitting.^[^ ^22^, ^23^, ^24^, ^25^ ^]^ There are two primary approaches for the (photo)electrochemical synthesis of H_2_O_2_: the water oxidation reaction (WOR, Equation (1))^[^ ^26^, ^27^, ^28^, ^29^ ^]^ and the oxygen reduction reaction (ORR).^[^ ^30^, ^31^, ^32^, ^33^ ^]^ The ORR can be conducted through either a direct one‐step two‐electron (2e^−^) mechanism (Equation (2))^[^ ^34^ ^]^ or a sequential two‐step single‐electron mechanism (Equation (3) and (4)).^[^ ^35^, ^36^ ^]^

(1) $$2\;H_{2}O+2\;h^{+}\to H_{2}O_{2}+2\;H^{+}\;\;\;\;\;\;\;\;E^{\theta }=+1.76\;V\;\text{vs}.\;\text{NHE},\;pH=0$$

(2) $$O_{2}+2\;H^{+}+2\;e^{-}\to H_{2}O_{2}\;\;\;\;\;\;\;\;\;\;\;\;\;\;E^{\theta }=+0.68\;V\;\text{vs}.\;\text{NHE},\;pH=0$$

(3) $$O_{2}+e^{-}\to O_{2}^{\cdot -}\;\;\;\;\;\;\;\;\;\;\;\;\;\;\;\;\;\;\;\;\;\;\;\;\;\;\;\;\;\;\;\;\;E^{\theta }=-0.33\;V\;\text{vs}.\;\text{NHE},\;pH=0$$

(4) $$O_{2}^{\cdot -}+e^{-}+2\;H^{+}\to H_{2}O_{2}\;\;\;\;\;\;\;\;\;\;\;\;\;\;\;\;E^{\theta }=+1.44\;V\;vs.\;\text{NHE},\;pH=0$$

(Photo)electrochemical synthesis via the two‐electron ORR (2e^−^ ORR) pathway provides a sustainable and practical method for on‐site H_2_O_2_ production under ambient conditions, offering an advantage over the traditional energy–intensive thermocatalytic approach.^[^ ^37^, ^38^, ^39^, ^40^, ^41^, ^42^, ^43^, ^44^, ^45^, ^46^, ^47^ ^]^ In the electrocatalytic ORR process, the four‐electron (4e^−^) pathway (Equation (5)) is thermodynamically favored over the desired two‐electron (2e^−^) pathway (Equation (2)),^[^ ^48^ ^]^ presenting a long‐standing challenge for catalyst design in achieving selectivity for H_2_O_2_ formation.^[^ ^49^ ^]^

(5) $$O_{2}+4\;e^{-}+4\;H^{+}\to 2\;H_{2}O\;\;\;\;\;\;\;\;\;\;\;\;\;E^{\theta }=+1.23\;V\;\text{vs}.\;\text{NHE},\;pH=0$$

Therefore, the success of this technology is critically dependant on developing high‐performance electrocatalysts that efficiently and selectively reduce oxygen to H_2_O_2_.^[^ ^50^, ^51^, ^52^, ^53^, ^54^ ^]^

In the context of light‐driven water splitting, two main approaches are typically explored. In photovoltaic‐electrolysis (PV‐E) light harvesting, photovoltaic (PV) cells are combined with water electrolysis. PV‐E is attractive because it allows independent optimization of light absorption for generating electricity and the production of fuels or chemicals via electrolysis.^[^ ^55^ ^]^ Alternatively, the direct utilization of solar energy in photoelectrochemical (PEC) cells is also commonly discussed.^[^ ^56^, ^57^, ^58^, ^59^, ^60^ ^]^ Direct PEC catalysis and systems offer the potential to utilize solar energy input to circumvent the four‐electron (4e^−^) ORR pathways (Equation (5)) toward two‐electron (2e^−^) ORR processes (Equation ((2), (3))–(4)), thereby enabling efficient production of H_2_O_2_.^[^ ^61^, ^62^, ^63^, ^64^, ^65^, ^66^, ^67^, ^68^ ^]^

**Figure 1:**
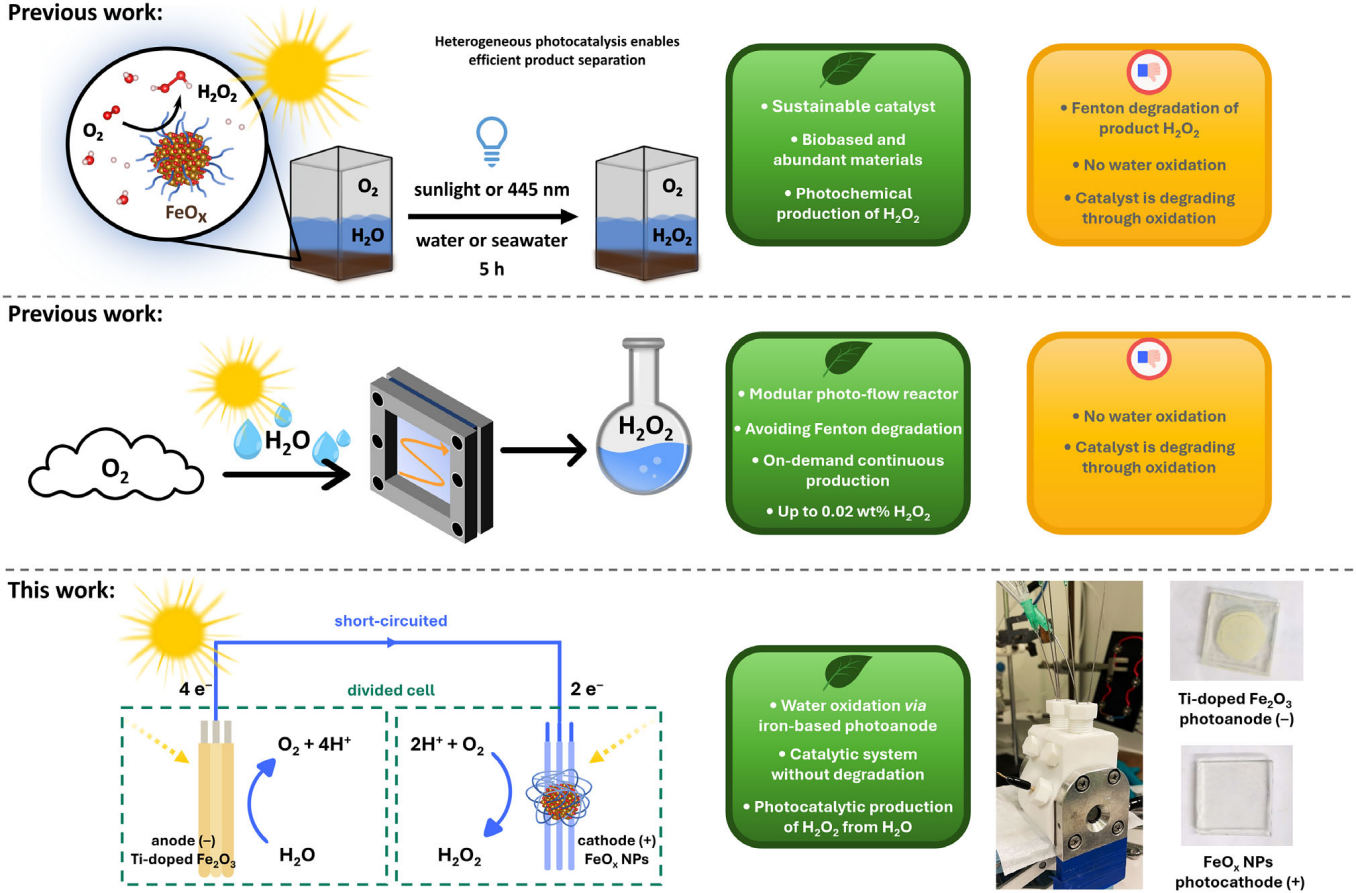
Schematic representation of advantages and merits of our previous work and our solutions for it. Photochemical production of hydrogen peroxide (H_2_O_2_) through iron‐oxide promoted oxygen reduction (ORR, top). Avoiding Fenton degradation of product H_2_O_2_ through a modular flow‐reactor (middle). PEC production of H_2_O_2_ via precious metal‐free photosynthesis of H_2_O_2_ from H_2_O (bottom).

Noble metals and their alloys are well‐studied catalysts for (photo)electrosynthesis of H_2_O_2_.^[^ ^69^, ^70^, ^71^, ^72^ ^]^ However, their scarcity and high cost pose significant barriers to their widespread application, leading to the development of sustainable alternatives.^[^ ^73^, ^74^, ^75^, ^76^, ^77^, ^78^, ^79^ ^]^ Based on these considerations, we previously developed a strategy for the photochemical production of H_2_O_2_ (9.4–14.8 mmol g^−1^ L^−1^) using earth–abundant iron.^[^ ^31^, ^80^ ^]^ By strictly adhering to the principles of Green and Sustainable Chemistry, we synthesized various iron oxide (FeO_x_) nanoparticles (NPs) capable of efficiently converting oxygen into H_2_O_2_ through ORR under visible light (445 nm), with >99% selectivity and an apparent quantum yield (AQY_445 nm_) of 0.11% (**Figure** **1** top).^[^ ^31^, ^81^ ^]^ In this work, we determine for the first time the oxygen‐to‐iron stoichiometric ratio (*x*) in FeO_x_, based on a combination of newly measured and recalculated data from our earlier study (see Results and Discussion). Crucially, the FeO_x_ NPs could be synthesized from biobased and abundant materials, including metal, surfactant (i.e., capping agent, ligand) and solvent. Photochemical production of H_2_O_2_ (9.4–14.8 mmol g^−1^ L^−1^) was also achieved under real sunlight irradiation and in seawater. We further designed a modular thin–film photo–flow reactor to increase production of H_2_O_2_ through minimizing Fenton degradation and facilitating product H_2_O_2_ separation from the catalyst surface.^[^ ^82^ ^]^ We successfully increased the production by 14.3x and productivity by 3.6x when compared to our original batch conditions (from 0.136 to 1.938 μmol and from 0.027 to 0.097 μmol h^−1^). This approach enabled continuous, on–demand production of H_2_O_2_, ultimately yielding solutions up to 0.02 wt% through water evaporation (Figure 1 middle).

A key issue in both batch and flow conditions, is that the catalyst exhibited deactivation after several cycles of oxygen reduction catalysis to H_2_O_2_. The absence of WORs or other sacrificial agents meant that the generated holes were not adequately compensated for; instead, electrons from Fe^2+^ in the FeOx NPs filled these holes, leading to gradual oxidation to Fe^3+^ and eventual catalyst deactivation.^[^ ^31^ ^]^

Building on these insights, we employed the designed FeO_x_ photocatalyst as a photoelectrode by depositing the FeO_x_ NPs onto a conductive fluorine‐doped tin oxide (FTO)–coated glass substrate, producing an H_2_O_2_‐generating photocathode. Our objective was to pair the FeO_x_ photocathode with a water–oxidizing photoanode, thereby supplying the electrons required for the ORR and enabling H_2_O_2_ photosynthesis from water. After thorough review of the literature,^[^ ^83^, ^84^, ^85^, ^86^, ^87^, ^88^, ^89^, ^90^ ^]^ we chose to combine the FeO_x_ photocathode (+) with Ti‐doped α‐Fe_2_O_3_ as photoanode (−).^[^ ^91^ ^]^ To maintain our focus on sustainability, we selected hematite (α–Fe_2_O_3_) based photoanodes for their well–documented properties and promising applications.^[^ ^23^, ^86^, ^92^ ^]^ Specifically, Ti‐doped α‐Fe_2_O_3_ was chosen for its facile synthesis and enhanced photochemical water‐oxidation performance afforded by titanium doping.^[^ ^91^ ^]^ Coupled with our FeO_x_ nanoparticle photocathode, this photoanode offers a promising route to harness solar energy for water splitting and store it as H_2_O_2_ solar fuel.

After mapping the properties of both photoelectrodes, each material was analyzed through cyclovoltammetry (CV) under electrochemical and PEC conditions. The FeO_x_ nanoparticle photocathode (+) was then paired with the Ti‐doped α‐Fe_2_O_3_ photoanode (−), enabling bias‐free operation (i.e., without external potential). Under simulated solar irradiation, this configuration achieved both WOR and ORR while preserving the structural integrity of the FeOx photocathode (+) without degradation or oxidation. By overcoming the limitations of our previous system, we established an iron‐based photoanode–photocathode pair for precious‐metal‐free photocatalytic H_2_O_2_ production from H_2_O as a high‐energy fuel (Figure 1 bottom).

## 2 Results and Discussion

Guided by the design strategy outlined above, we prepared the FeO_x_ photocathode and Ti‐doped α‐Fe_2_O_3_ photoanode and characterized them by scanning electron microscopy (SEM), energy‐dispersive X‐ray spectroscopy (EDX), dynamic light scattering (DLS) and solid–state UV–vis spectroscopy. Subsequently, we investigated the electrocatalytic and photoelectrocatalytic behavior of the photocathode and photoanode separately to assess their mutual compatibility. Finally, we combined the two photoelectrodes in a single device, with the aim to investigate their paired performance in the bias–free light‐driven H_2_O_2_ production from water, using a tandem PEC cell.

Characterization of the FeO_x_ (*x* ≈ 1.06–1.25) NPs showed that the oxygen‐to‐iron stoichiometric ratio varied with the analytical method used. X‐ray photoelectron spectroscopy (XPS) gave lower values, reflecting surface‐sensitive measurements, whereas bulk elemental analysis yielded higher values due to the inclusion of Fe^3+^‐rich core regions. Such differences are consistent with previous reports on nanoscale iron oxides, where surface Fe^2+^ enrichment relative to the bulk arises from synthesis conditions, particle size effects, and surface ligand interactions.^[^ ^93^, ^94^, ^95^, ^96^ ^]^ We therefore represent the material as mixed‐valence FeO_x_ (*x* ≈ 1.06–1.25), with full calculation details provided in the Supplementary Information (ESI 4.3 and 4.3.1).

### 2.1 Photoelectrode Preparation and Properties

**Figure 2:**
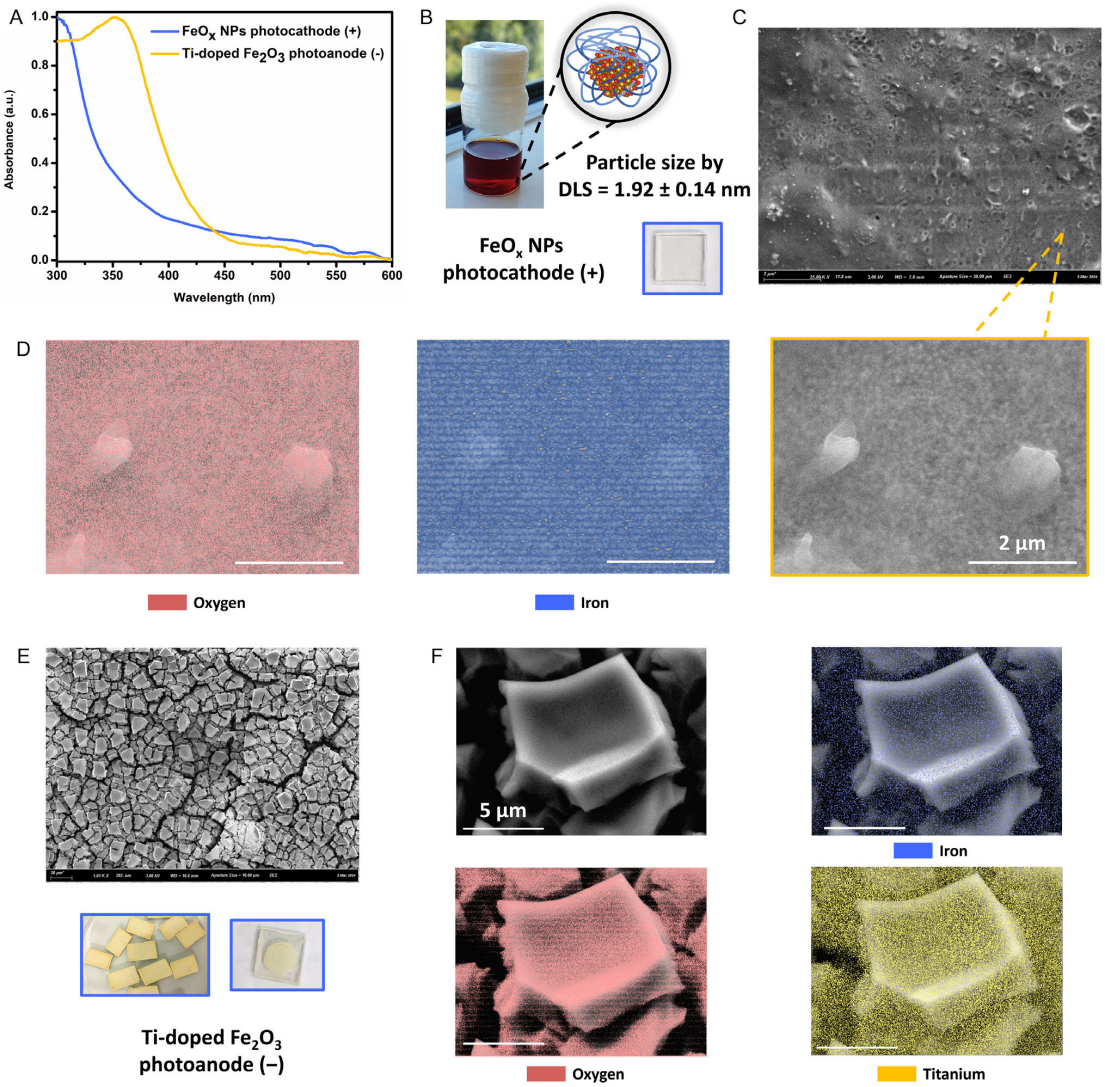
Main properties of the photoelectrodes. A) Solid‐state UV–Vis spectroscopy of the FeO_x_ NPs photocathode (+) and Ti@α‐Fe_2_O_3_ photoanode (–). B) Image of the suspended FeO_x_ nanoparticles in organic solvent, image of the FeO_x_ photocathode and its particle size (1.92 ± 0.14 nm) measured through DLS. C) Scanning electrode microscopy and D) EDX of the FeO_x_ photocathode. E) Image and scanning electrode microscopy and F) EDX of Ti@α‐Fe_2_O_3_ photoanode.

Both Fe‐based electrodes were prepared on FTO‐coated glass. The FeO_x_ NPs were synthesized according to our earlier reported procedure,^[^ ^31^ ^]^ and DLS measurements confirmed the FeO_x_ NPs had an average size of 1.92 ± 0.14 nm, allowing us to use this batch for the photocathode material (**Figure** **2**B). Prior to applying this catalyst batch on the FTO, we conducted benchmark photocatalytic studies as previously reported, confirming successful production of H_2_O_2_ through the ORR (Figure S13, Supporting Information). The FeO_x_ nanoparticle electrodes were subsequently prepared according to the previously established methodology by preparing a FeO_x_ nanoparticle suspension with Nafion, followed by drop‐casting on FTO and air drying.^[^ ^31^ ^]^

The Ti‐doped α‐Fe_2_O_3_ electrodes were prepared according to an adjusted literature procedure,^[^ ^91^ ^]^ following a deposition‐annealing (DA) methodology. The thickness and composition of the electrodes was controlled by the number of DA cycles as well as the concentration of FeCl_3_ and Ti precursor solutions. Further details on the preparations are described in the Supplementary Information.

Following the fabrication of the electrodes, we performed SEM and energy‐dispersive EDX to characterize their material properties. A homogeneous distribution of iron (blue) and oxygen (red) was confirmed on the FeO_x_ photocathode surface (Figure 2C and D). Similarly, SEM and EDX analyzes of the Ti‐doped α‐Fe_2_O_3_ photoanode (Figure 2E and F) revealed a consistent electrode preparation with a uniform distribution of iron (blue), oxygen (red), and titanium (yellow). Notably, the FeO_x_ NPs photocathode appeared transparent, likely due to the small size of the NPs, while the Ti‐doped α‐Fe_2_O_3_ photoanode exhibited a yellow/brown color, with TiO_2_ regions appearing white, making the electrode brighter than the typical hematite brown.

Solid state UV–Vis provided further insight into the light‐absorption properties of the electrodes. Compared to pure FTO and Nafion–coated FTO, both iron‐based photoelectrodes exhibited light absorption up to 600 nm (Figure 2A). The additional absorption band at 380 nm observed in the Ti–doped α‐Fe_2_O_3_ photoanode is attributed to TiO_2_ (Figure S16, Supporting Information).

To assess the electrochemical and PEC properties of the electrodes, we separately studied the redox properties of anode and cathode, in a three‐electrode setup. We investigated the electrodes by cyclic voltammetry (CV), both in the dark as well as under illumination. In these experiments, the photoelectrode was used as the working electrode (WE), an Ag/AgCl electrode in 3 M KCl as the reference electrode (RE), and a Pt wire as the counter electrode (CE). Finally, before pairing the electrodes in the bias‐free device, we studied the PEC behavior of the individual electrodes in a three‐electrode configuration, in the same device that we planned to pair the electrodes in for bias‐free water splitting to H_2_O_2_. Here, we examined the photocatalytic behavior of the materials via chopped‐light linear sweep voltammetry (LSV) and chopped‐light amperometry. In these latter experiments, the photoelectrode functioned again as WE, Ag/AgCl (3 M KCl) as RE, and a FTO plate with electrodeposited Pt as CE.

### 2.2 (Photo)electrochemical Characterization of the FeO_x_ Photocathode

**Figure 3:**
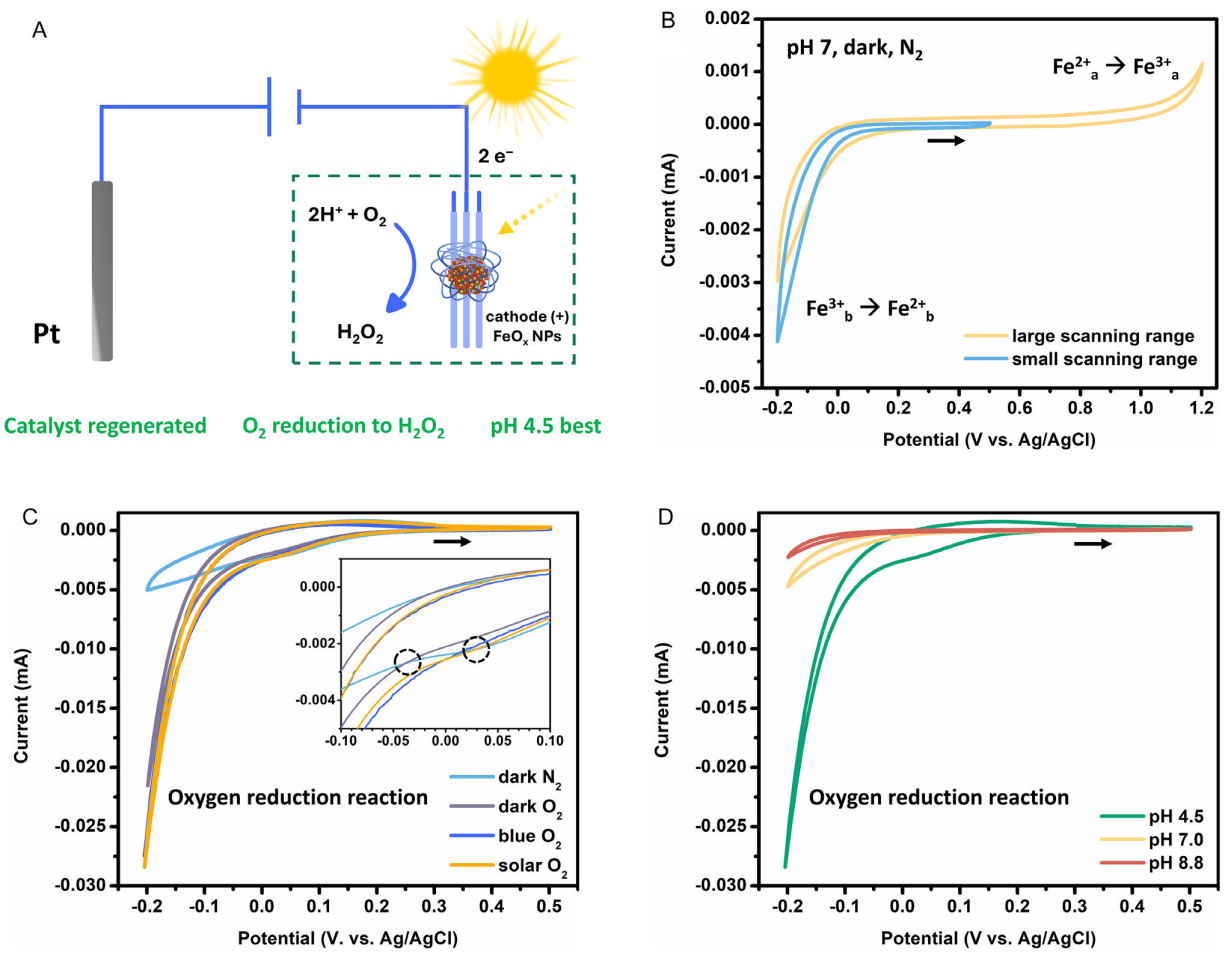
An overview of the electrochemical and PEC properties of the FeO_x_ photocathode (+), determined via CV studies in a three‐electrode setup in an undivided heart‐shaped cell, using the FeO_x_ NP catalyst @ FTO as the WE, Ag/AgCl (in 3 M KCl) as the RE, and Pt wire (*d* = 0.5 mm) as the CE. Unless otherwise specified, for all CV measurements shown we applied 0.85 × iR drop compensation. The second scans are shown, starting at 0.3 V, scanning toward anodic potential with a scan rate of 25 mV s^−1^. A) Schematic representation of the three‐electrode setup for CV in the heart‐shaped cell. B) Cyclic voltammograms of the FeO_x_ NP photoelectrode in darkness while varying the potential range from wide (yellow) to narrow (blue). The CVs were measured at pH 7 in Milli‐Q buffer solution (1:3 50 mM NaH_2_PO_4_: 50 mM Na_2_HPO_4_) purged with N_2_. Two nonoverlapping redox events are observed, which are attributed to distinct Fe sites within the material, denoted as Fe^2+^_a_/Fe^3+^_a_ at more positive potentials and Fe^3+^_b_/Fe^2+^_b_ at more negative potentials. C) Cyclic voltammograms of the n‐type FeO_x_ NP photoelectrode under a N_2_ atmosphere in the dark (light blue) and under an O_2_ atmosphere in the dark (gray), irradiated with blue light (blue), and a solar simulator (yellow). The CVs were measured at pH 4.5 in Milli‐Q buffer (50 mM NaH_2_PO_4_). D) Cyclic voltammograms of the FeO_x_ NP photoelectrode under an O_2_ atmosphere, illuminated with a solar simulator, while varying the pH of the electrolyte from pH 4.5 (green) to pH 7.0 (yellow) to pH 8.8 (red).

The redox properties of the FeO_x_ NPs photocathode were first evaluated in a three‐electrode setup under standard conditions (pH = 7, darkness, N_2_ atmosphere) using a heart–shaped electrochemical cell, both in the dark and under illumination (setup schematically shown in **Figure** **3**A).

The CV measurements (Figure 3B) revealed two distinct, nonoverlapping redox processes. First, at more positive potentials (above +0.8 V vs. Ag/AgCl) the Fe^2+^_a_ sites in the Fe_3_O_4_–like material were readily oxidized to Fe^3+^_a_ — a process previously associated with catalyst deactivation.^[^ ^31^, ^97^, ^98^ ^]^ To avoid this overoxidation and potential deactivation of the Fe^2+^_a_ sites, we narrowed the potential range between −0.2 and 0.5 V for further measurements (Figure 3B, blue line).

Notably, a second redox process could be observed. Even in the absence of O_2_ (i.e., without the possibility of oxygen reduction), we observed cathodic currents associated with the reduction of Fe^3+^_b_ to Fe^2+^_b_, suggesting that FeO_x_ NPs can regenerate catalytically active Fe^2+^ species in situ under electrochemical conditions. This regeneration capacity implies that the material can maintain catalytic function over time in a PEC system.^[^ ^64^, ^65^ ^]^ These two redox processes occur at different potentials and likely involve distinct populations of Fe sites (a and b) on the photocatalyst surface, indicating they do not belong to a single reversible Fe^2+^/Fe^3+^ couple.

Upon replacing the inert N_2_ atmosphere with O_2_ (Figure 3C), a significant increase in current was observed.^[^ ^64^, ^65^ ^]^ In darkness (Figure 3C, gray line), the current increased more than fourfold at −0.2 V, from −0.005 to − 0.022 mA cm^−2^, with a catalytic onset potential around −0.036 V vs. Ag/AgCl (dashed circle, left). Catalytic oxygen reduction was further enhanced under irradiation with blue light LEDs (445 nm) or sunlight (1 sun) from a solar simulator, as indicated by the further increase in photocurrent (−0.029 mA cm^−2^) and the even earlier catalytic onset around +0.031 V (dashed circle, right).

A noteworthy observation is the catalytic current observed in darkness under O_2_ atmosphere, which is likely due to a shift in the Fermi level induced by the applied potential, enabling electron transfer to O_2_ even without illumination. Under illumination, conduction band electrons are additionally supplied via photoexcitation, allowing catalytic current to arise at more cathodic potentials (see Figure 3C inset). This behavior is consistent with FeO_x_ acting as an n*‐*type semiconductor, where photogenerated electrons in the conduction band drive the ORR. Furthermore, upon illumination, not only does the catalytic overpotential decrease, but the catalytic current also increases. Specifically, the catalytic current increased an additional 41% (at a potential of −0.2 V vs. Ag/AgCl), from −0.022 to −0.029 mA cm^−2^. Thus, under illumination, only the valence band of the FeO_x_ NP catalyst needs to be replenished, allowing catalytic ORR to initiate at lower overpotential and proceed with higher catalytic current densities. This demonstrates the advantage of a PEC setup for the FeO_x_ NP photocathode.

Having successfully achieved photoelectrocatalytic oxygen reduction to H_2_O_2_ with FeO_x_ NPs—without catalyst deactivation, as supported by XPS analysis of the regenerated active species—we further investigated the pH dependance of the oxygen reduction process (Figure 3D). We studied the photoelectrocatalytic behavior of the catalyst under mildly acidic (pH 4.5), neutral (pH 7.0), and slightly basic (pH 8.8) conditions, finding the highest photocurrents at pH 4.5. This optimum arises because the slightly acidic environment increases proton availability, which facilitates the proton‐coupled electron transfer steps in both ORR pathways, leading to improved charge transfer and faster reaction kinetics—consistent with our previous experimental findings.

In an additional CV measurement (ESI 11.1.4), we initially operated under N_2_ atmosphere to reduce Fe^3+^ to Fe^2+^, then switched to an O_2_ atmosphere during the sequential measurement (Figure S22, Supporting Information). This revealed that even with short exposure to O_2_‐saturated atmosphere, ORR took over, with the artificially overpopulated active sites (Fe^2+^) reacting back to their initial state (Fe^3+^) while maintaining ORR through successful photoelectrocatalysis, facilitated by the replenishment of electrons in the system.

### 2.3 (Photo)electrochemical Characterization of the Ti‐Doped α–Fe_2_O_3_ Photoanode

The next step involved examining the PEC properties of the Ti‐doped α‐Fe_2_O_3_ photoanode for its ability to catalytically stimulate the WOR.

**Figure 4:**
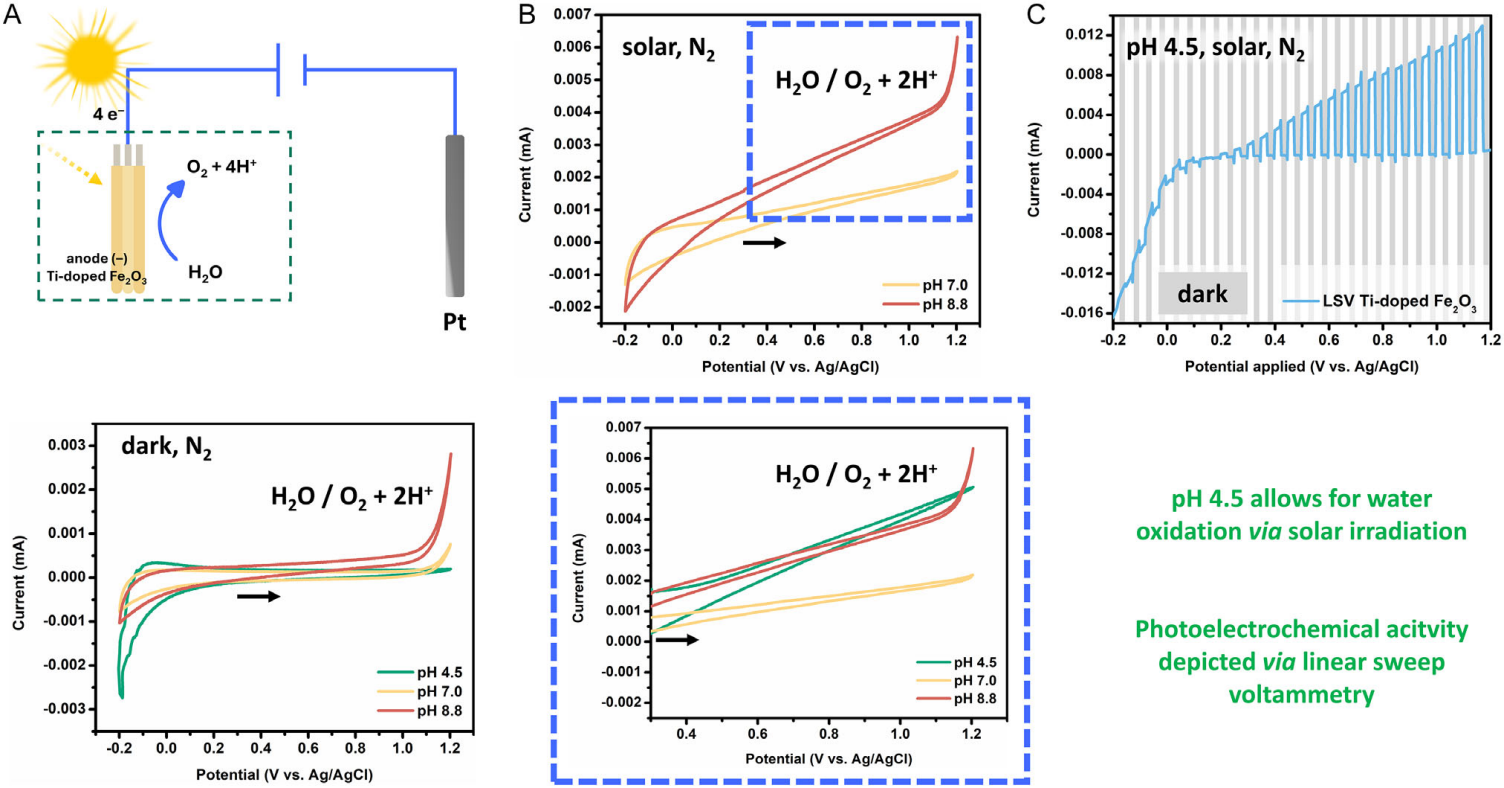
An overview of the electrochemical and PEC properties of the Ti‐doped α‐Fe_2_O_3_ photoanode (*–*) determined via CV studies in a three‐electrode setup in an undivided heart‐shaped cell, using Ti‐doped α‐Fe_2_O_3_ photoanode @ FTO as the WE, Ag/AgCl (in 3 M KCl) as the RE, and Pt wire (*d* = 0.5 mm) as the CE. Unless otherwise specified, for all CV measurements shown, we applied 0.85 × iR drop compensation. The second scans are shown, starting at 0.3 V, scanning toward anodic potential with a scan rate of 25 mV s^−1^. A) (A top) Schematic representation of the setup for cyclovoltammetry. (A bottom) Cyclic voltammograms of the Ti‐doped α‐Fe_2_O_3_ photoelectrode under an N_2_ atmosphere in the dark showing pH dependency. The CVs were measured in Milli‐Q buffers at pH 4.5 (50 mM NaH_2_PO_4_), pH 7.0 (1:3 50 mM NaH_2_PO_4_: 50 mM Na_2_HPO_4_) and at pH 8.8 (50 mM Na_2_HPO_4_). B) (B top) Cyclic voltammograms of the Ti‐doped α‐Fe_2_O_3_ photoelectrode under an N_2_ atmosphere under solar simulator (1 sun) illumination showing pH dependency; (B bottom) zoomed into WOR potential range. C) Chopped LSV with 5 s light on/off cycles on Ti‐doped α‐Fe_2_O_3_ WE, equipped with a FTO | Pt CE and a Ag/AgCl (in 3 M KCl) RE. The sweep was carried at out 5 mV s^−1^ from −0.2 to 0.5 V in Milli‐Q buffer at pH 4.5 (50 mM NaH_2_PO_4_). Illumination was done with solar simulator (1 sun).

To evaluate the water oxidation performance of the Ti‐doped α–Fe_2_O_3_ photoanode, we performed CV in a three‐electrode setup using a platinum wire (Pt) as the CE and Ag/AgCl (in 3 M KCl) as the RE (**Figure** **4**A, top). Under dark conditions and a nitrogen atmosphere, the photoanode exhibited pH‐dependent electrochemical activity (Figure 4A, bottom). At pH 8.8, anodic currents consistent with the WOR (2 H_2_O to O_2_ and 4 H^+^) were observed, while at pH 4.5 the electrode remained largely inactive (Figure 4A, bottom). These findings indicate that under purely electrochemical conditions, water oxidation is only favorable in alkaline media. This behavior poses a challenge for constructing a full electrochemical cell under bias‐free conditions, as the WOR requires basic pH (optimal at pH 8.8), whereas the ORR on the photocathode is favored in acidic conditions (optimal at pH 4.5).

Upon illumination with a solar simulator (1 sun), however, the photocurrent increased significantly at all pH values (Figure 4B), indicating that photogenerated charge carriers enable water oxidation even under otherwise unfavorable acidic conditions. The photoanode still performed best in basic conditions (with photocurrents of 0.0062 mA cm^−2^), but it also demonstrated sufficient activity in the less favorable acidic conditions, exhibiting photocurrents of 0.005 mA cm^−2^ (Figure 4B, bottom), suggesting the potential for operation outside of the optimal dark electrochemical regime. Furthermore, under illumination and in acidic conditions, the Ti‐doped α‐Fe_2_O_3_ photoanode exhibited a pronounced PEC onset, with a significant current enhancement observed across the potential range of 0.4 to 1.2 V versus Ag/AgCl (Figure 4B, top).

To ensure that this response originated from a photocatalytic process, we carried out chopped–light voltammetry, where we varied the potential while turning the solar light source on and off. The chopped‐light voltammetry experiments were carried out in the PEC cell (Figure S27, S28, Supporting Information), where we combined the Ti–doped α‐Fe_2_O_3_ photoanode as WE with electrodeposited Pt @ FTO as CE and Ag/AgCl (3 M KCl) as RE. The resulting chopped‐light voltammogram is displayed in Figure 4C, where the illuminated periods are indicated by the white panels and the dark periods by the gray panels. Under illumination, the voltammogram shows a similar response as the green curve in Figure 4B (bottom), and in the dark, it clearly resembles the green curve in Figure 4A (bottom). This resemblance does not change after repeated illumination cycles, indicating that the material itself remains intact and that the high photocurrent response most likely originates from photoelectrocatalytic water oxidation.

Overall, the photoanode is thus capable of catalytically oxidizing water under solar illumination. We hence demonstrated that both photoelectrodes are active at mildly acidic conditions (pH 4.5) while illuminated and can thus, in theory be paired in a bias‐free PEC cell to convert water and oxygen to H_2_O_2_.

### 2.4 Combined System Under Bias‐Free Conditions

Prior to combining both Fe‐based photoelectrodes, they were independently investigated as working electrodes (WE) in the PEC cell with Pt | FTO as the CE and Ag/AgCl (in 3 M KCl) as the RE. Having confirmed that the Ti‐doped α‐Fe_2_O_3_ photoanode could facilitate water oxidation under solar irradiation, chopped‐light chronoamperometry measurements showed enhanced photocurrents during the 10 s illumination period, compared to the alternating 10 s periods of darkness (Figure S30, Supporting Information). LSV experiments were performed for the FeO_x_ NP electrodes as WE, with Pt | FTO CE and Ag/AgCl (3 M KCl) as RE. The FeO_x_ NP electrodes demonstrated some photoelectrocatalytic activity, by showing very low photocurrents (1.1 μA cm^−2^, Figure S31, Supporting Information), when irradiated with a solar simulator.

**Figure 5:**
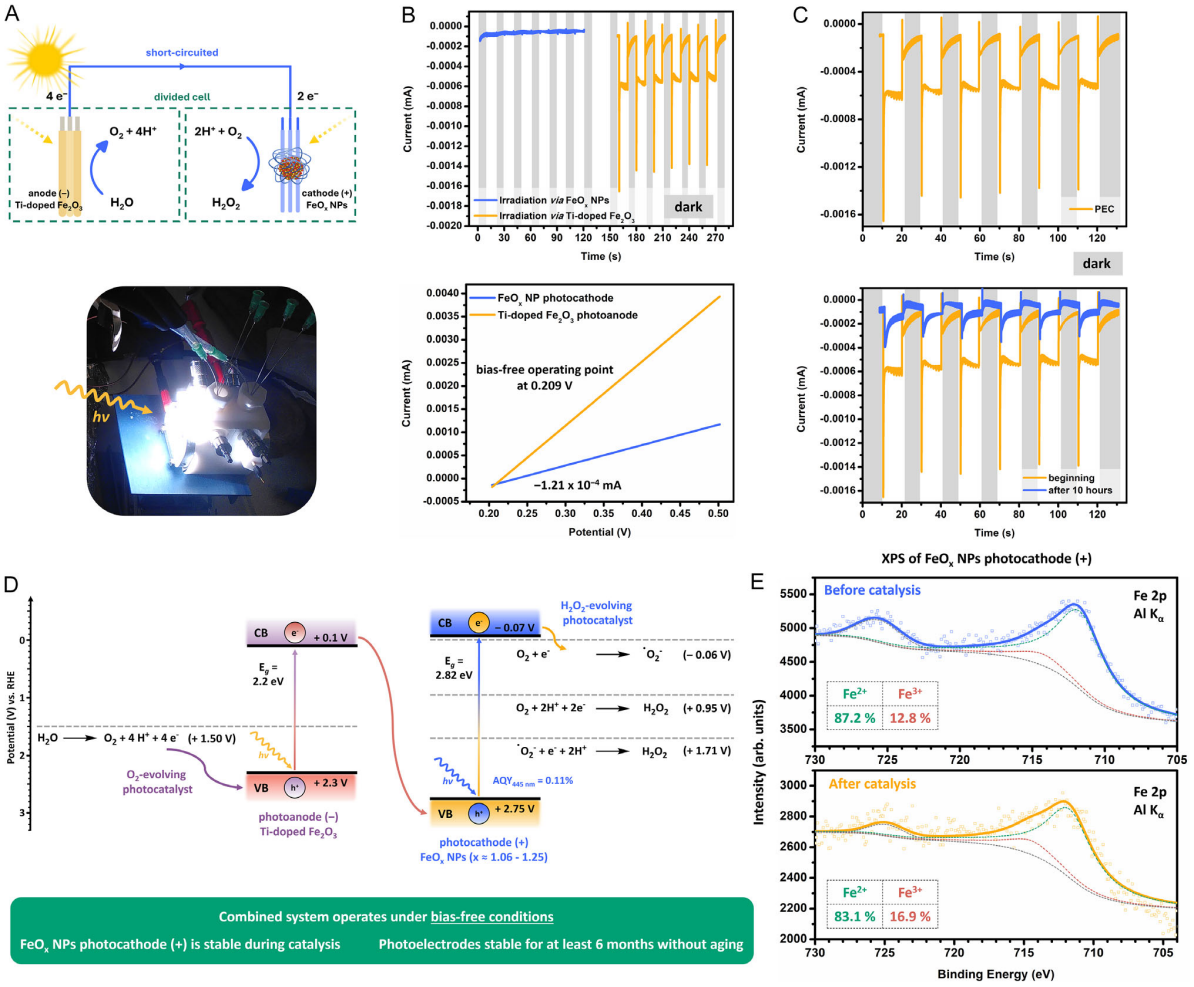
An overview of the PEC properties of the FeO_x_ NP photocathode (+) and Ti‐doped α‐Fe_2_O_3_ photoanode (*–*) determined via chopped‐light amperometry at 0.0 V in a bias‐free system in pH 4.5 Milli‐Q buffer (50 mM NaH_2_PO_4_). The cell was illuminated with a solar simulator (1 sun) for periods of 10 s followed by 10 s of darkness. A) Schematic representation and image of the combined system for photosynthesis of hydrogen peroxide from water under bias‐free conditions. B) (B top) Investigation for side of irradiation for optimal activity. Successful PEC operation of FeO_x_ NPs photocathode (+) with the Ti‐doped α‐Fe_2_O_3_ photoanode (*–*), enabling WOR and ORR to H_2_O_2_. (B bottom) Intersection of interpolated LSV current data of the FeO_x_ NP photocathode (blue) and Ti‐doped Fe_2_O_3_ photoanode (orange) plotted against a common potential axis calculated from LSV potential data recorded vs. Ag/AgCl as RE. C) Long term irradiation for photoelectrocatalytic production of H_2_O_2_. D) Z‐scheme energy diagram of Ti‐doped α‐Fe_2_O_3_/FeO_x_ at pH 4.5 (vs. RHE). Photogenerated holes in α‐Fe_2_O_3_ drive water oxidation (OER, +1.50 V), while electrons in FeO_x_ reduce O_2_ to H_2_O_2_ via indirect one‐electron (O_2_^•−^, –0.06 V and +1.71 V) pathways. Band alignment enables bias‐free operation in the tandem system, with photocurrent limited by the small driving force for the initial 1 e^−^ ORR step. The pure photochemical FeO_x_ step achieved an apparent quantum yield (AQY_445 nm_) of 0.11% under 445 nm monochromatic irradiation. E) XPS on the FeO_x_ NPs photocathode to compare before and after catalysis confirming catalyst stability: the Fe 2*p* core level region is presented; data are plotted as dots, the corresponding fits as continuous lines; the percentages correspond to the relative spectral intensities of the two components.

After characterizing the properties of both photoelectrodes, we combined the FeO_x_ NPs photocathode (+) with the Ti‐doped α–Fe_2_O_3_ photoanode (−) to test under bias‐free conditions (i.e., without external potential) (**Figure** **5**A). We combined the two electrodes in a custom‐made PEC cell,^[^ ^99^, ^100^ ^]^ where the photocathodic and photoanodic compartments were separated by a proton exchange membrane. Initially, we investigated which side (FeO_x_ NPs or Ti‐doped α‐Fe_2_O_3_) required irradiation for catalytic activity. We proceeded with this investigation by performing bias‐free, two‐electrode chopped‐light amperometry experiments with alternating 10 s periods of illumination and darkness (Figure 5B, top, indicated by the white and gray panels, respectively). Since the FeO_x_ NP electrode was fully transparent, and the Ti‐doped was not, we hypothesized it would be most productive to irradiate through the photocathode, since this way most photons would reach both electrodes. Surprisingly, we barely obtained any photocurrent by irradiating through the FeO_x_ NP photocathode (Figure 5B, top, blue). On the other hand, when performing the same experiment by illuminating through the Ti‐doped α‐Fe_2_O_3_ photoanode, measurable photocurrents were observed, which sustained catalytic activity over multiple on‐off cycles (Figure 5B, top, orange). We speculate that charge separation close to the junction between the FTO and Ti‐doped α‐Fe_2_O_3_ is important for the photoanode to function, but further research would be needed to elucidate the effect of the illumination direction on the photoanode performance.

LSV measurements of the FeO_x_ nanoparticle photocathode and the Ti‐doped α‐Fe_2_O_3_ photoanode, plotted on a common potential scale (converted from values recorded vs. Ag/AgCl), revealed an intersection at ≈0.209 V **(**Figure 5B, bottom). At this potential, both electrodes deliver matched photocurrents of about −1.21 × 10^−4^ mA, corresponding to the bias‐free operating point of the tandem PEC cell. The negative sign of the current indicates the cathodic nature of the process at the photocathode. At this operating point, the photocatalytic WOR and ORR are successfully paired to produce H_2_O_2_ from water and oxygen, confirming that the system can sustain net photocurrent generation under illumination without the need for an external bias. The chopped‐light amperogram under illumination through the photoanode (Figure 5C, top) shows several notable features. Firstly, the slow decay of current observed during dark periods is attributed to capacitive discharge within the photoelectrode system. This capacitive behavior reflects charge accumulation during illumination, which is gradually released after the light is turned off—distinct from the sharper current transitions observed in Figure 4C. Secondly, the system maintained stable photoelectrocatalytic activity under continuous irradiation (1 sun) for extended periods of up to 10 h. After 10 h of irradiation, the photocurrent reduced from 0.8 to 0.3 μA cm^−2^ (Figure 5C, bottom).

### 2.5 Mechanism and Catalyst Stability

As shown in Figure 5D, Ti–doped α‐Fe_2_O_3_ (VB ≈ +2.30 V; CB ≈ +0.10 V) and FeO_x_ (VB ≈ +2.75 V; CB ≈ −0.07 V) form a Z‐scheme pair. Upon illumination, both semiconductors generate electron–hole pairs. The photogenerated holes in the valence band of Ti‐doped α–Fe_2_O_3_ (VB = +2.3 V vs. RHE) oxidize water to O_2_ at +1.50 V vs. RHE, while electrons in the conduction band of α‐Fe_2_O_3_ recombine with holes in the valence band of FeO_x_. Here, the conduction band of FeO_x_ (−0.07 V vs. RHE) provides sufficient thermodynamic driving force for the reduction of O_2_, which may proceed via two plausible pathways under the slightly acidic conditions used (pH 4.5).

One‐electron pathway (stepwise formation of H_2_O_2_ via superoxide)

(6) $$O_{2}+e^{-}\to O_{2}^{\cdot -}\;\;\;\;\;\;\;\;\;\;\;\;\;\;\;\;\;\;\;\;\;\;\;\;\;\;\;\;E_{\text{RHE}}^{0}=-0.06\;V$$

The superoxide intermediate is then reduced in a second electron‐transfer step, coupled with proton uptake, to form H_2_O_2_.

(7) $$O_{2}^{\cdot -}+e^{-}+2\;H^{+}\to H_{2}O_{2}\;\;\;\;\;\;\;\;\;\;\;\;\;\;\;\;\;\;\;\;E_{\text{RHE}}^{0}=+1.71\;V$$

Under the slightly acidic reaction conditions, the O_2_•^−^ species exists in equilibrium with its protonated form HO_2_•, but the net pathway is captured by the above two‐step electron transfer. In our previous study, active‐species trapping experiments confirmed the involvement of superoxide radicals (O_2_•^−^), indicating that the FeO_x_ catalyst facilitates an indirect 2e^−^ oxygen reduction (ORR: +0.11 V vs. RHE, pH = 7) via O_2_•^−^ without significant overpotential.

Direct two‐electron pathway

(8) $$O_{2}+2\;H^{+}+2\;e^{-}\to H_{2}O_{2}\;\;\;\;\;\;\;\;\;\;E_{\text{RHE}}^{0}=+0.95\;V$$

This pathway bypasses discrete O_2_•^−^ accumulation and produces H_2_O_2_ in a concerted two‐electron step.

In both scenarios, α‐Fe_2_O_3_ supplies oxidative power for O_2_ evolution, while FeO_x_ provides reductive power for selective H_2_O_2_ generation, completing the bias‐free Z‐scheme. The modest overall photocurrent (0.8 → 0.3 μA cm^−2^) likely reflects the small driving force (≈10 mV) between the FeO_x_ conduction band and the 1e^−^ O_2_/O_2_•^−^ potential, rendering interfacial electron transfer and O_2_ transport kinetically sluggish, whereas water oxidation on α‐Fe_2_O_3_ remains thermodynamically well‐driven (more details in ESI 12.7).

Given that the paired photoelectrode configuration was designed to prevent Fe^2+^ oxidation and maintain catalyst stability, we assessed the FeO_x_ photocathode before and after catalysis using XPS. Results are shown in Figure 5E and in the Supplementary Information (ESI 13., Figure S34–36, Table S5, 6, Supporting Information). The analysis of the Fe 2*p* spectrum indicates successful application of the FeO_x_ NPs as photocatalyst (Fe^2+^ at a binding energy of 711.7 eV and Fe^3+^ at 714.3 eV). While the overall signal–to‐noise ratio decreased postcatalysis, possibly due to nanoparticle leaching, active sites of the FeO_x_ NPs were successfully replenished through the PEC reactions occurring on the photocathode. Notably, the FeO_x_ NPs photocathode remained stable during catalysis, as evidenced by CV and XPS analyzes. In our previous study, FeO_x_ NPs prior to catalysis exhibited an Fe^2+^/Fe^3+^ ratio of 83% and 17%, respectively, which shifted to 54% Fe^2+^ and 46% Fe^3+^ upon oxidation during deactivation.^[^ ^31^ ^]^ In this study, the application and regeneration of FeO_x_ NPs within the photoelectrode system maintained a stable catalytic state, with the Fe^2+^/Fe^3+^ ratio changing only slightly from 87.2%/12.8% before catalysis to 83.1%/16.9% after catalysis. Additionally, the FeO_x_ NPs photocathode remained stable for at least six months without signs of ageing during storage in the dark.

Thus, addressing the limitations of our previous system, we successfully employed an iron‐based photoanode as well as photocathode for precious metal‐free photocatalytic production of H_2_O_2_ from H_2_O as a high‐energy fuel.

In future research, two challenges must be tackled that are still present in the current system. As mentioned before, the FeO_x_ NPs could potentially be leaching off the electrode. Alternative methods to integrate the catalyst on the FTO electrode could result in a higher stability of the system.^[^ ^101^ ^]^ Furthermore, we were unable to quantify the H_2_O_2_ production in the device presented herein. Since H_2_O_2_ is prone to Fenton degradation on iron–surfaces, we hypothesize that the product is degraded before detection was possible.^[^ ^64^, ^102^, ^103^ ^]^ Therefore, we will next apply the PEC system in flow conditions, to remove the H_2_O_2_ from the photoreactor and minimize Fenton degradation, as well as to utilize the O_2_ generated by the WOR.

Through the integration of our FeO_x_ ORR catalyst with a WOR catalyst within a photoelectrode system, we effectively maintained the structural integrity of the FeO_x_ NPs photocathode (+) without experiencing degradation or oxidation during the reaction, thereby enabling genuine photocatalytic production of H_2_O_2_.

## 3 Conclusion

In this investigation, we have introduced a bias‐free PEC cell to convert H_2_O and O_2_ to H_2_O_2_, by pairing two iron‐based photoelectrodes. At the same time, we effectively addressed the deactivation pathways of our earlier developed FeO_x_ NPs ORR photocatalyst by integrating it within the photocathode. This approach circumvented catalyst deactivation via oxidation through continuous operation and facile electron replenishment. Following a thorough exploration of the PEC behavior of both electrodes, the FeO_x_ NPs photocathode (+) was successfully integrated with the Ti–doped α‐Fe_2_O_3_ photoanode (–) for operation under bias‐free conditions (i.e., without external potential). The iron‐based Ti–doped α‐Fe_2_O_3_ photoanode facilitated the WOR, synergistically complementing the FeO_x_ ORR photocathode. Using (simulated) sunlight as energy source, robust PEC performance was achieved, enabling simultaneous WOR and ORR while preserving the structural integrity of the FeO_x_ NPs photocathode (+) without degradation or oxidation. Through adherence to the principles of Green and Sustainable Chemistry in the synthesis of both catalysts and in their operation, we successfully demonstrated the functionality of a precious metal–free artificial leaf for the photosynthesis of hydrogen peroxide from water.

## Supporting Information

The data supporting this article have been included as part of the Supplementary Information.

## Conflict of Interest

The authors declare no conflict of interest.

## Supporting information

Supplementary Material

## Data Availability

The data that support the findings of this study are available from the corresponding author upon reasonable request.
