# Supplementary material for: Precious Metal‐Free Artificial Leaf for Photosynthesis of Hydrogen Peroxide from Water
Source: ChemSusChem. 2025 Sep 17;18(21):e202501055. doi: 10.1002/cssc.202501055 (PMC12584969; doi:10.1002/cssc.202501055)
Supplement: Supplementary file 1 — Supplementary Material [file CSSC-18-e202501055-s001.pdf]

Supplementary Information

for

Precious metal-free artificial leaf for photosynthesis of hydrogen peroxide from water

Thomas Freese,[a] Alexandra Matei,[a] Maria B. Brands,[b] Marina Karsakova,[a] Diego A. Acevedo-Guzmán,[c] Dominic Gerlach,[c] Petra Rudolf,[c] Joost N. H. Reek,[b] and Ben L. Feringa*[a]

a. Stratingh Institute for Chemistry, University of Groningen, Nijenborgh 4, 9747 AG Groningen, The Netherlands

b. van 't Hoff Institute for Molecular Sciences, University of Amsterdam, Science Park 904, 1098 XH Amsterdam, The Netherlands

c. Zernike Institute for Advanced Materials, University of Groningen, Nijenborgh 4, 9747AG Groningen, The Netherlands

E-Mail: b.l.feringa@rug.nl

Table of Contents

1. Materials and methods 4
   1.1 Reagents and solvents 4
2. Equipment and general analytical information 5
3. Experimental procedures 7
   3.1 Synthesis of iron oxide nanoparticles with oleic acid 7
   3.2 Synthesis of iron oxide nanoparticles with oleic acid: troubleshooting 9
   3.3 Sustainable storage of iron oxide nanoparticles with oleic acid, U.S. Environmental Protection Agency (EPA) ban on methylene chloride (dichloromethane, DCM). 11
4. Catalyst properties 13
   4.1 DLS results and size in different solvents 13
   4.2 Transmission electron microscopy (TEM), scanning transmission electron microscopy (STEM) and energy-dispersive X-ray spectroscopy (EDX) of FeO_x NPs 14
      4.2.1 FeO_x NP with oleic acid (*cis*) 15
   4.3 Detailed calculation of FeO_x species: 17
      4.3.1 Summary and conclusion: 20
   4.4 UV-Vis studies on FeO_x NPs suspensions 20
5. Irradiation studies 21
   5.1 Batch photocatalytic oxygen reduction 21
   5.2 Screening and batch irradiation of NPs synthesized in ethanol 22
6. Quantification of hydrogen peroxide 23
   6.1 Peroxide test strips 23
   6.2 Iodometric titration 24
      6.2.1 Quantification procedure 24
7. List of photochemical H_2O_2 production values 24
8. Preparation of electrodes 25
   8.1 Cleaning of FTO-coated glass 25
   8.2 Cutting of FTO-coated glass 25
   8.3 Preparation of Ti-doped α-Fe_2O_3 nanoparticle electrodes 25
   8.4 Preparation of FeO_x nanoparticles (FeO_x NP) on FTO electrodes 26
   8.5 Preparation of Pt | FTO counter electrodes for photoelectrochemistry 26
9. Properties of photoelectrodes 27
   9.1 Solid state UV-Vis spectroscopy on photoelectrodes and references 27
   9.2 Scanning electron microscopy (SEM) and energy-dispersive X-ray spectroscopy (EDX) of photoelectrodes 27

10. Electrochemistry 27
10.1 Electrolyte solutions 27
11. Cyclic voltammetry 28
11.1 FeO_x photocathode. 30
11.1.1 Blanks with Nafion (at pH 4.5) 30
11.1.2 Dark N_2, pH dependency 30
11.1.3 Dark N_2, pH 4.5 different scan rates 31
11.1.4 pH 4.5 dark, short O_2 atmosphere 32
11.2 Ti-doped α-Fe_2O_3 photoanode 33
11.2.1 Blanks with FTO 33
11.2.2 Irradiation with blue LED 34
11.2.3 Solar N_2, pH dependency 35
12. Photoelectrochemistry 36
12.1 General setup 36
12.2 Electrode assembly for photoelectrochemical cell 38
12.3 Performance photoanodes 39
12.4 Performance photocathodes 40
12.5 LSV integration of photocathode and photoanode 41
12.5.1 Methodology and Data 41
12.5.2 Notes about methodology 45
12.5.3 Conclusion 46
12.6 Paired bias-free photoelectrochemistry experiments 47
12.7 Detailed thermodynamic analysis and mechanism 48
12.7.1 pH referencing and RHE conversion 48
12.7.2 Thermodynamic potentials at pH 4.5 48
12.7.3 Band edge positions at pH 4.5 49
12.7.4 Thermodynamic feasibility 49
12.7.5 Complete electron/charge flow in the α-Fe_2O_3/FeO_x Z-scheme at pH 4.5 (vs. RHE) 50
12.7.6 Z-scheme operation 51
12.7.7 Implication for the observed small photocurrent (0.8 → 0.3 $\mu A\ cm^{-2}$) 51
13. XPS Analysis 52
13. References 58

1. Materials and methods

1.1 Reagents and solvents

Commercial reagents and solvents: Unless stated otherwise, all reagents and solvents were obtained from the commercial sources: Sigma–Aldrich, TCI, Boom and Linde-gas and were used as received. For aqueous solutions, Milli-Q water was used.
The following chemicals were purchased from *Sigma Aldrich*:
Iron (0) pentacarbonyl (Sigma Aldrich, 99.99%, SHBN5572, 09-05-22), oleic acid (Sigma Aldrich, 90%, MKCL2492, 21-07-20), trimethylamine N-oxide dihydrate (Sigma Aldrich, 98%, BCCF8795, 08-07-22), hydrochloric acid (Sigma Aldrich, 37%, STBK5583, 20-06-22), starch (Sigma Aldrich, SLCC5527, 21-01-21), sodium thiosulfate (Sigma Aldrich, 99%, BCCC5894, 21-01-21), potassium iodide (Sigma Aldrich, 99.5%, STBJ7197, 21-01-21), ammonium molybdate tetrahydrate (Sigma Aldrich, 81-83%, SLCH3187, 21-01-21), sodium hydroxide (Sigma Aldrich, 98%, SLCC5278, 07-01-20), 4-Methyl-2-pentanone (Sigma Aldrich, >98.5%, BCCJ6219, 26-09-23), 2-Butanone (Sigma Aldrich, >99.0%, SHBQ0260, 04-10-23), tert-Butyl methyl ether (Sigma Aldrich, >99.0%, SHBQ2623, 26-09-23).
The following chemicals are purchased from *Boom B.V.*:
Sulfuric acid (Boom B.V., 95-97%, 26-10-22), ethanol (Boom B.V., 100%, EA99-4422-10SD, 16-12-22), ammonia solution (Boom B.V., 25%, 14-09-21), ethyl acetate (Boom B.V., technical grade, PROD2303098, 26-02-24), tetrahydrofuran (Boom B.V., 100%, PROD2100983, 16-09-21).

The following chemicals are purchased from *Acros Organics*: iron (0) pentacarbonyl (Acros Organics, A0425102, 24-12-20), purchased from *TCI*: Dimethyl carbonate (TCI, >98%, DEQYA-LF, 30-06-22), 2-Methyltetrahydrofuran (TCI, >98%, stabilized with BHT, QRCVM-AT, 24-03-22), purchased from *Macron*: Dichloromethane (Macron, UN1593, 13-07-22), purchased from Honeywell: Pentane (Honeywell, >95.0%, Lot No. L1230, 26-02-24), purchased from *Linde-gas*: Oxygen compressed 5,0 (technical grade, SOL SpA, S161280921X01247DI, 01-09-22).

The following chemicals are purchased from *Alfa Aesar:* Nafion (D-521 dispersion, 5% w/w in water and 1-propanol, ≥ 0.92 meq/g exchange capacity), purchased from *Fisher Scientific*: $FeCl_3 \cdot 6\,H_2O$ (54 mg, 99 %).

Fluorine-doped tin oxide glass slides were purchases from Sigma Aldrich (30 × 30 cm, 2.3 mm thickness, 13 Ω/sq).

A Nafion proton exchange membrane (N-117, FuelCellStore) was used to divide the photocathodic and photoanodic compartment in the experiments with the photoelectrochemical cell.

2. Equipment and general analytical information

Photochemical equipment:

- LEDs (OSRAM Oslon SSL 80 royal blue, LDCQ7P-2U3U, 500 mW, λ = 445 nm, 180 mW/cm^2) as light source for batch production of hydrogen peroxide and irradiation during cyclic voltammetry and paired photoelectrochemistry measurements.
- An Oriel LCS-100 Series small area solar simulator (Sol1A, M94011A, Newport) equipped with a 100 W Xe lamp (Newport). To determine position and distance to the heart-shaped cell and the photoelectrochemical cell, a calibrated reference silicon solar cell (Oriel model 91150V, Newport) was used to measure solar simulator irradiance in "sun" units, where 1 sun corresponds to 100 mW/cm^2 of light with the solar emission spectrum (AM 1.5G). The solar simulator was placed at 1.0 sun from the (photo)electrochemical cell.

Electrochemical equipment:

- The electrochemical measurements were recorded on an Autolab PGSTAT101 potentiostat (Metrohm) equipped with NOVA 2.0 software. The measurements were performed in a heart-shaped cell equipped with a leakless Ag/AgCl (3 M KCl) reference electrode (eDAQ, ET069) and a Pt wire (diameter 0.5 mm) counter electrode. All the measurements were carried out at room temperature, and all measurements were iR drop compensated by 85%.

pH measurements:

- The pH values were determined with a SI Analytics Handylab 100 pH meter equipped with a SI Analytics pH electrode BlueLine 14 pH. The setup was calibrated with pH 4, 7 and 10 reference standard buffers purchased from Sigma Aldrich.

Hotplate and furnace for electrode synthesis:

- 5-minute annealing at 350 °C was done on a hotplate using a Programmer PR5 to control the temperature.
- 550 °C annealing was carried out in a Carbolite Gero CWF 1100 chamber furnace.

General Analytical Information: Nuclear Magnetic Resonance spectra were measured with an Agilent Technologies 400-MR (400/54 Premium Shielded) spectrometer (400 MHz). All spectra were measured at room temperature (22–24 °C). Chemical shifts for the specific NMR spectra were reported relative to the residual solvent peak [in ppm; $CDCl_3$: δH = 7.26; $CDCl_3$: δC = 77.16]. The multiplicities of the signals are denoted by s (singlet), d (doublet), t (triplet), q (quartet), m (multiplet), br s (broad signal), app (apparent). All ^{13}C-NMR spectra are ^{1}H-broadband decoupled.

High-resolution mass spectrometric measurements were performed using a Thermo scientific LTQ OrbitrapXL spectrometer with electrospray ionization. The molecular ion (M^+, $[M + H]^+$ and $[M–X]^+$) is given in m/z-units.

UV-vis spectra were recorded with an Agilent 8543 spectrophotometer. The Agilent 8453 UV-Visible spectrometer was equipped with a custom-built (Prizmatix/Mountain Photonics) multi-wavelength fiber coupled LED-system (FC6-LED-WL) including the following LEDs: 365A, 390B, 420Z, 445B, 535R, 630CA. A detailed description of the setup was published earlier by our group (see Figure S1 in reference 1).[1] A Quantum Northwest TC1 temperature controller was used to maintain the temperature at 20 °C during photochemical studies.

Dynamic light scattering (DLS) experiments were performed with a Zetasizer Ultra Red (Malvern Panalytical, ZSU3305).

A Tecnai T20 cryo-electron microscope with 200 keV was used to take the Transmission electron microscopy (TEM) and scanning transmission electron (STEM) images. Energy-dispersive X-ray spectroscopy (EDX) was performed with a silicon drift energy dispersive X-ray (SDD EDX) detector X-max from Oxford Instruments. The elemental ratio was calculated *via* INCA software.

X-ray photoelectron spectroscopy (XPS) was performed using a Surface Science Instruments SSX-100 ESCA spectrometer, equipped with a monochromatic Al Kα X-ray source (hv =1486.6 eV). The pressure in the measurement chamber was maintained below $5 * x10^{-9}$ mbar during data acquisition. The photoelectron take-off angle was 37° with respect to the surface normal. The diameter of the analyzed area was 1000 µm; the energy resolution was 1.26 eV (or 1.67 eV for a broad survey scan). A more detailed description can be found in **Section 13**.

3. Experimental procedures

3.1 Synthesis of iron oxide nanoparticles with oleic acid

Oleic acid (2.20 g (90%), 7.01 mmol, 2.31 eq.), ethanol (12 mL) and iron pentacarbonyl (0.4 mL, 3.04 mmol, 1 eq.) were added to a 100 mL two-neck round bottom flask and the mixture heated at reflux (150°C Allihn condenser) for 1h at a stirring speed of 660 rpm (**Figure S1**). After 1h the mixture had turned from yellow to orange/brown (**Figure S2 (A)**). The mixture was initially cooled using an ice bath and then with a 20°C water bath to *room temperature* (20°C) **(B)**, while filling the decreasing volume of the headspace with nitrogen. To the cooled mixture, trimethyl amine N-oxide dihydrate (1 g (98%), 8.82 mmol, 2.90 eq.) was added while flushing the system with nitrogen, which resulted in bubbling and a dark solution **(C)**. The mixture was then heated to 130°C for 2h under nitrogen atmosphere resulting in a color change to yellow **(E)**, where overpressure of gas (CO) was allowed to leave the system in the first few minutes **(D)**. After 2h the temperature was increased to 150°C and kept for 1h. The reaction was stopped by cooling with an ice bath and subsequently with a water bath until but *not further* than 20°C (whole cooling process: 1 min, **F**), while flushing the headspace with nitrogen. The obtained iron oxide nanoparticles were decanted into a beaker (500 mL, **G**) and settled from ethanol (200 mL). While rinsing the round bottom flask with the ethanol, the magnetic nanoparticle droplets were already formed inside the flask **(H)**. The magnetic nanoparticles were pulled out of solution (212 mL) by a magnet under the beaker for 1h (**Figure S3 I, J**). The ethanol was decanted off and the particles were washed once with ethanol (50 mL) **(K, L)** and then dried *via* constant air flow. Next, the particles were suspended in dichloromethane (10 mL) or other, more sustainable solvent alternatives (*see below*) and stored under nitrogen atmosphere and in darkness at 5 °C. The particles were stable in DCM, 2-MeTHF, THF or MIBK for at least 6 months without aging, agglomeration or changes in size as shown in **Figure S3** and **Table S1**. The approximate yield was 7-34% corresponding to 100-500 mg varying per batch.[2]

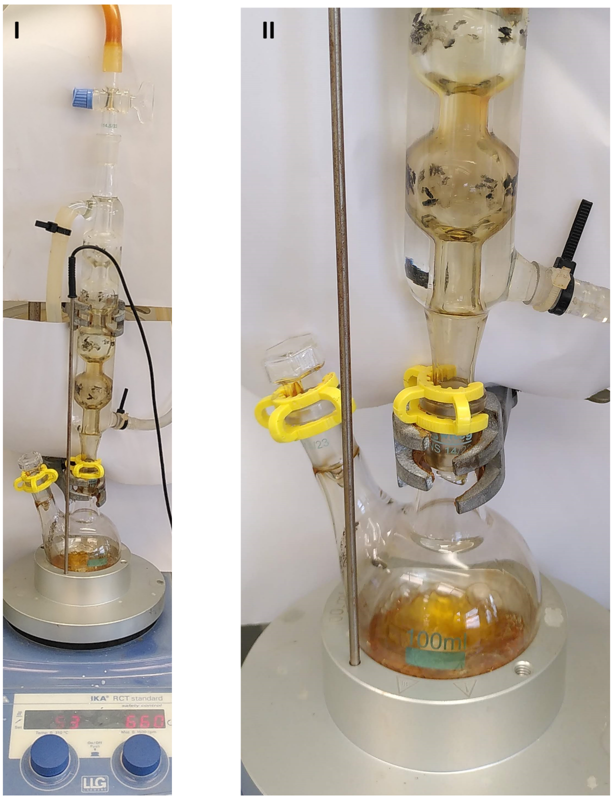

Figure S1: Synthesis equipment used for the FeO_x nanoparticle synthesis. Two-neck round bottom flask equipped with an Allihn condenser and heated *via* metal heating mantle.[2]

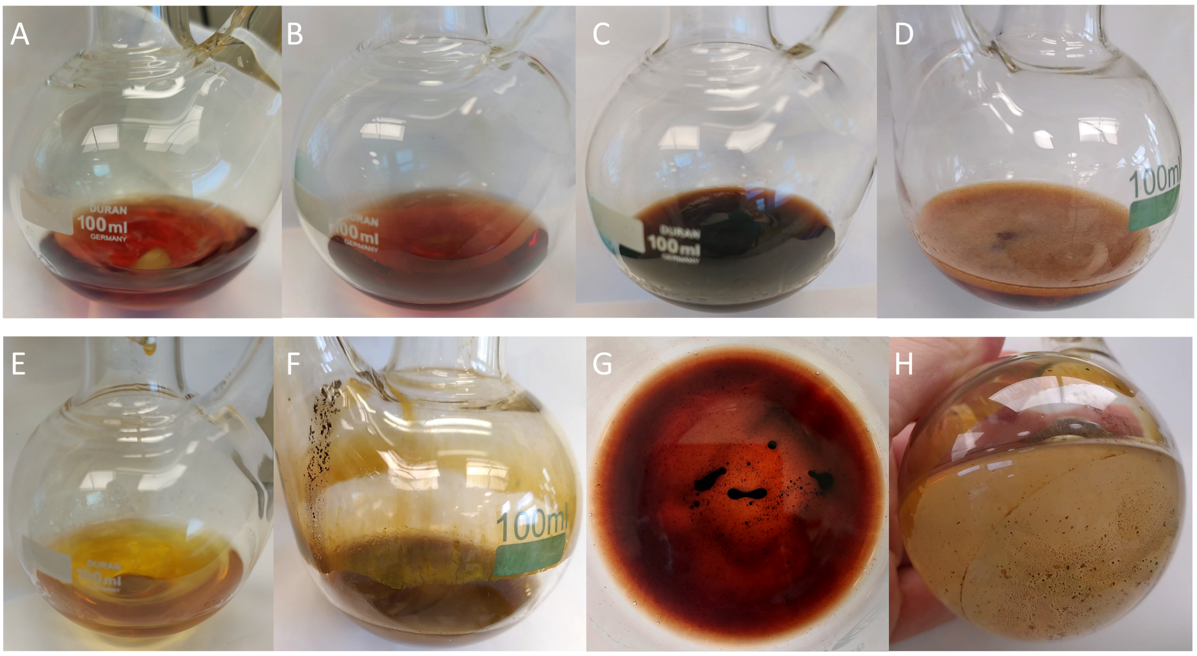

Figure S2: Photographs of FeO_x NPs (batch 141) with oleic acid surfactant 2:1. A) t=50 min, B) t=60 min cooled, C) cooled at t=60 min Me_3N added, D) heating up at t=63 min and gas formation E) t=100 min, F) cooled solution after synthesis, G) precipitation on magnet, purely decanted, H) washing and rinsing of round bottom flask for droplet formation.[2]

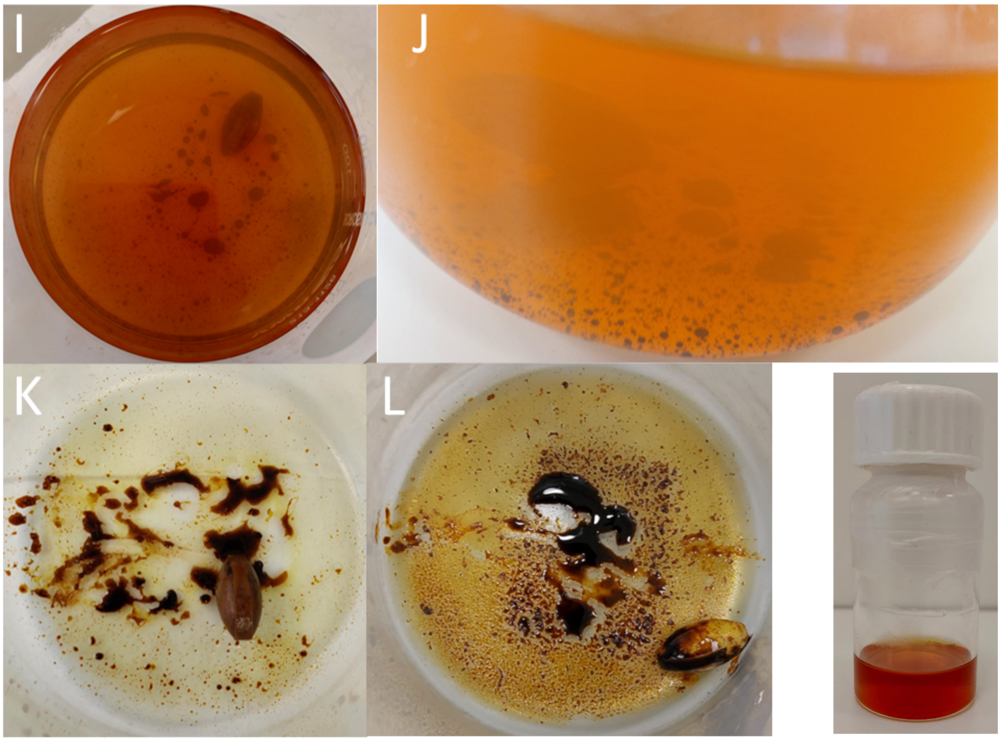

Figure S3: Precipitation on top of a magnet of FeO_x NPs (batch 141) with oleic acid 2:1 as surfactant. I,J) 1h washing with 200 mL EtOH and droplet formation, K,L) washed FeO_x NPs with ethanol (50 mL). Photograph of FeO_x NPs (batch 153) with oleic acid 2:1 in DCM after 12h - the dispersion is stable, and no precipitates were identified (bottom right).[2]

3.2 Synthesis of iron oxide nanoparticles with oleic acid: troubleshooting

The synthesis was found to be consistent (>240 batches) and could successfully be reproduced by four different researchers in different labs at different locations (a bachelor student at Linnaeusborg (University of Groningen), a master student at Linnaeusborg (University of Groningen), and two PhD candidates at Nijenborgh & Linnaeusborg (University of Groningen) and at University of Amsterdam). Especially for the synthesis of heterogeneous catalyst materials, reproducibility is crucial and often an overlooked aspect.[2] Successful synthesis was independent of iron(0) pentacarbonyl suppliers (Sigma Aldrich, Acros Organics) with different Lot-numbers, coming from different continents.[2]

Unsuccessful syntheses of a few batches led to extensive troubleshooting. The importance of dry conditions was discovered when once 96% ethanol was used instead of the usual 100% ethanol: big pieces were floating around in the flask after addition of trimethylamine N-oxide dihydrate and heating to reflux, where usually with 100% ethanol a completely dissolved and homogeneous solution is obtained. A similar phenomenon was observed when water was once utilized as 1:1 cosolvent with ethanol, suggesting that water in the synthesis leads to undesired precipitation of nanoparticles out of solution. Wet nitrogen gas from the Schlenk-line also led to unsuccessful nanoparticle synthesis; here the nanoparticles did not magnetically precipitate from the solution during workup. Over time stirring bars became yellow/brown after repeatedly being used for synthesis and cleaning using hydrochloric acid solution (37%), which led to particles not magnetically precipitating during workup. By using new stirring bars, we were able to overcome this problem. Over time iron(0) pentacarbonyl was found to precipitate as a solid in the normally yellow liquid, which led to differently looking pieces magnetically precipitating in the workup. We suspect that precipitation was caused by the septum on the bottle being punctured too often, therefore not sufficiently sealing the nitrogen atmosphere in the bottle anymore. Iron(0) pentacarbonyl is a pyrophoric compound, meaning that it could react with air to burn to iron(III) oxide.[8] By switching to a new bottle of iron(0) pentacarbonyl these problems could be avoided. When the trimethylamine N-oxide dihydrate was too dry (dry powder instead of hygroscopic white solid) inconsistent syntheses were observed. A ‘wet’ hygroscopic solid is recommended over a dry white powder. During the cooling steps of the reaction mixture nitrogen has to be flushed into the decreasing volume of the headspace. Furthermore, the cooling should be performed rapidly with an ice bath for up to 1 min, but not longer and the temperature should not drop below 20°C; this can be monitored by replacing the initial ice cooling with an additional water bath. Letting the solution drop below 16°C leads to precipitation of frozen oleic acid. These crystals will trap the 2 nm FeO_x onto 400-600 nm crystals of oleic acid, which is not desired. Extensive cooling also leads to gel/droplet/oil formation at the bottom of the round bottom flask, resulting in tedious workup and transfer to the beaker.[2]

Below a list of requirements is given as a guide, which should be consulted if there are difficulties in synthesis.

List of requirements for successful nanoparticle synthesis[2]

- 100% ethanol as solvent
- clean and dry glassware (100 mL 2-neck flask, Allihn condenser, adapter to Schlenk-line)
- clean stirring bar (egg shaped, 2 cm)
- clean Schlenk-line tubing
- dry nitrogen (make sure phosphorus pentoxide is still dry by moisture indicator)
- clean oil in Schlenk-line
- 660 rpm stirring speed
- sufficient reflux
- 500 mL beaker during workup on magnet
- cooling with the ice bath not below 20 °C to avoid precipitation of oleic acid (16 °C)
- hygroscopic trimethylamine N-oxide dihydrate

3.3 Sustainable storage of iron oxide nanoparticles with oleic acid, U.S. Environmental Protection Agency (EPA) ban on methylene chloride (dichloromethane, DCM).

Usually, the particles were suspended in dichloromethane (10 mL) and stored in nitrogen atmosphere and darkness at 5°C, where they were stable for >6 months. Recently though (30th April 2024), the United States Environmental Protection Agency (EPA) finalized a ban on most uses of methylene chloride (dichloromethane, DCM). Hence, for our catalyst system to stay relevant and especially align with its goal for sustainable production of H2O2 we investigated greener solvent alternatives.

Experimental procedure:

Several stable and active batches of FeO_x NPs stored in DCM were taken and combined. From the solution obtained the NPs were taken out *via* syringe and put into 8 different vials (4 mL of DCM and NPs per vial). The DCM was evaporated completely. Afterwards 4 mL of the respective greener alternative solvent was added to each vial, thereby maintaining the same concentration which was obtained through NP synthesis and storage in DCM. The NPs were redispersed through sonication and vortex shaking. The obtained NPs dispersions were stored in nitrogen atmosphere and darkness at 5°C, where they were checked for stability and precipitation on a regular basis (daily for a duration of 56 d).

Highly hazardous	Hazardous		Problematic		Recommended
Diethyl ether	Diisopropyl ether	MTBE	Acetone	MeOH	Water (H_2O)
Benzene	1,4-dioxane	THF	2-MeTHF	*t*-BuOH	EtOH
Pyridine	Dimethoxyethane	Formic acid	Heptane	Methyl ethyl ketone (MEK)	*i*-PrOH
Sulfolane	Pentane	Cyclohexane	Me-cyclohexane	MeOAc	*n*-BuOH
Chloroform	Hexane	Pyridine	*tert*-butyl acetate	AcOH	EtOAc
CCl_4	DMF		Toluene	Ac_2O	*i*-PrOAc
Dichloroethane	DMAc		Xylenes	Dimethyl carbonate (DMC)	*n*-BuOAc
Dichloromethane	NMP		Chlorobenzene	Cyclopentyl methyl ether (CPME)	Anisole
Nitromethane	Methoxy-ethanol		Acetonitrile	γ-Valerolactone (GVL)	Methyl isobutyl ketone (MIBK)
	Et_3N		DMPU	Hydroxymethylfurfural (HMF)	Cyclohexanone
			DMSO		Ethylene glycol
			Cyrene		Benzyl alcohol

Figure 1: Ranking of solvents with respect to their sustainability.[3–6]

Table S1: Stability test of nanoparticle dispersions in greener solvent alternatives following the ban on DCM. Stability was investigated daily for a duration of 56 d (4 mL solution). Size differences among solvents can be correlated to the solvation shell and measurement *via* dynamic light scattering and are not having an impact on photochemical activity. A Zetasizer Ultra Red (ZSU3305) from Malvern Panalytical was used for dynamic light scattering experiments. All samples (1 mL with a concentration of 1 mg mL^{-1}) were measured at 298.15 K. The particle size was measured *via* DLS (**Table S2**).

Ranking	Solvent alternative	Stability	Comments	Photo
1	2-MeTHF	Dispersion is stable and no precipitates were identified	Recommended	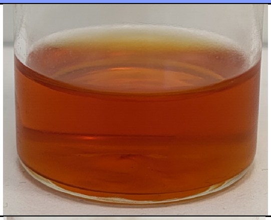
2	THF	Dispersion is stable and no precipitates were identified	-	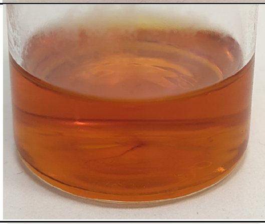
3	MIBK	Dispersion is stable and no precipitates were identified	Slightly less solubility than #1 and #2	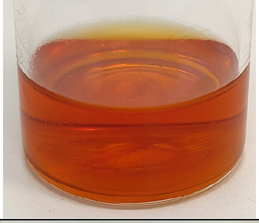
4	MTBE	Not stable	-	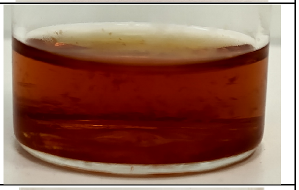
5	MEK	Not stable	-	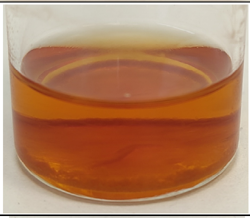
6	EtOAc	Not stable	-	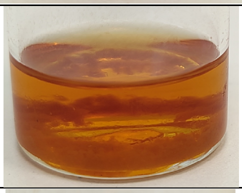
7	Pentane	Not stable	-	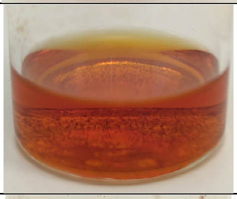
8	DMC	Not stable	-	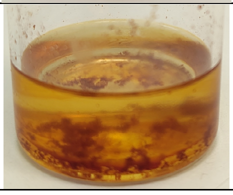

4. Catalyst properties

4.1 DLS results and size in different solvents

A Zetasizer Ultra Red (ZSU3305) from Malvern Panalytical was used for dynamic light scattering experiments. All samples (1 mL with a concentration of 1 mg mL^{-1}) were measured at 298.15 K.

Table S2: Particle size and diffusion coefficient of FeO_x NP scope with concentration 1 mg mL^{-1}, obtained *via* DLS measurements.

Surfactant (Batch #)	Measured in solvent	Particle size (nm) by number %	Diffusion coefficient ($\mu m^2\ s^{-1}$)
Oleic acid (173), Acros organics	DCM	1.88±0.37	8.13±1.49
Oleic acid (174), Sigma aldrich	DCM	1.97±0.31	7.27±1.29
Oleic acid (131)	THF	3.82±0.42	1.20±0.67
Oleic acid (B9, Dec. 2023)	DCM	2.52±0.15	-
Oleic acid (B9, Jun. 2024)	DCM	2.56±0.78	-
Oleic acid (B4-B6, Dec. 2023)	DCM	2.46±0.65	-
Oleic acid (B4-B6, Jun. 2024)	2-MeTHF	9.24±4.89	11.67±0.02
Oleic acid (B4-B6, Jun. 2024)	THF	11.51±6.81	10.96±0.54
Oleic acid (B4-B6, Jun. 2024)	MIBK	10.49±5.06	9.22±0.09

4.2 Transmission electron microscopy (TEM), scanning transmission electron microscopy (STEM) and energy-dispersive X-ray spectroscopy (EDX) of FeO_x NPs

The TEM, STEM, and EDX data shown are representative for FeO_x nanoparticles synthesized *via* our established, highly reproducible procedure (see EES Catal., 2024, 2, 262–275).[2]

TEM characterization:

A PHILIPS CM 120 Cryo electron microscope with 120 keV was used to take the TEM images. The sample grid was prepared by dropping 5 µL of the solution (1 mg/mL in THF or DCM) onto an ultrathin carbon film coated copper grid (or graphene grid in the case of oleic acid and linoleic acid). After 30 sec of drying the grid was washed with 5 µL ethanol (EtOH) and any surplus solvent was dried on a filter paper. In the case of oleic acid and linoleic acid (1 mg/mL THF), the graphene grids were placed in a desiccator at full vacuum over-night.

STEM characterization:

A Tecnai T20 cryo-electron microscope with 200 keV was used to take the Transmission electron microscopy (TEM) and scanning transmission electron (STEM) images. EDX analysis was performed with a SDD EDX detector from Oxford xmax instruments, and the elemental ratio was calculated *via* INCA software.

4.2.1 FeO_x NP with oleic acid (*cis*)

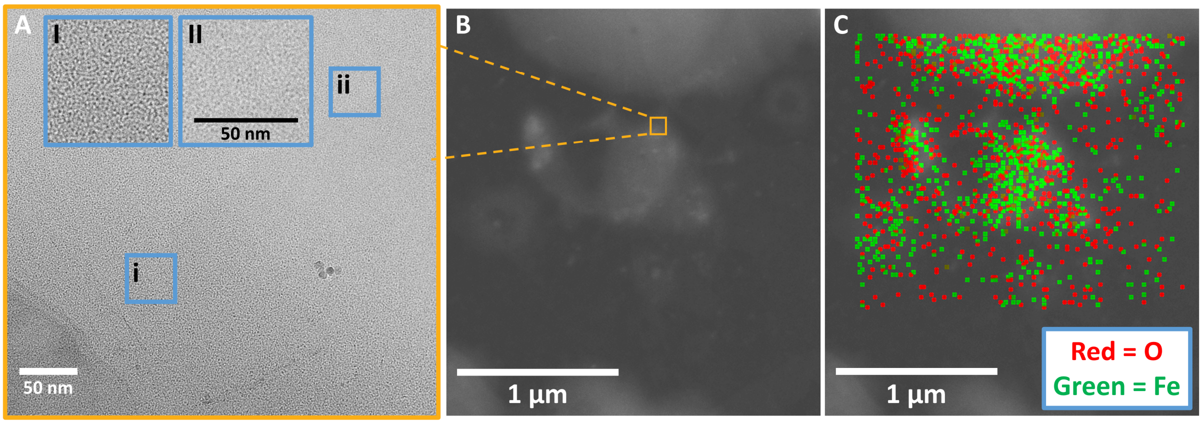

Figure S4: A) Transmission electron microscopy of FeO_x (batch 131, oleic acid 2:1, Acros Organics, 1 mg mL^{-1} in THF), at a magnification of 100000x (inlet: zoomed); particle size by DLS 1.94±0.34 nm. B) Scanning transmission electron microscopy of FeO_x (batch 131, oleic acid 2:1, 1 mg mL^{-1} in THF), inlet: zoom towards A. C) EDX of FeO_x (batch 131, oleic acid 2:1, 1 mg mL^{-1} in THF), drying spots of solvents contain more FeO_x NP and concentration decreases towards the edges of the droplets; oxygen is depicted in red – iron in green.[2]

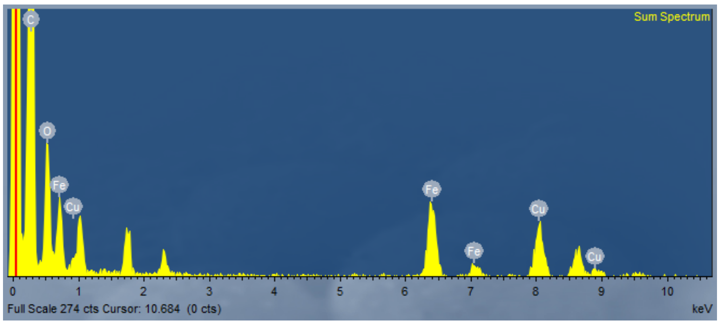

Figure S5: Full spectrum of the elemental analysis (EDX) of FeO_x (batch 131, oleic acid 2:1, Acros Organics, 1 mg mL^{-1} in THF).[2]

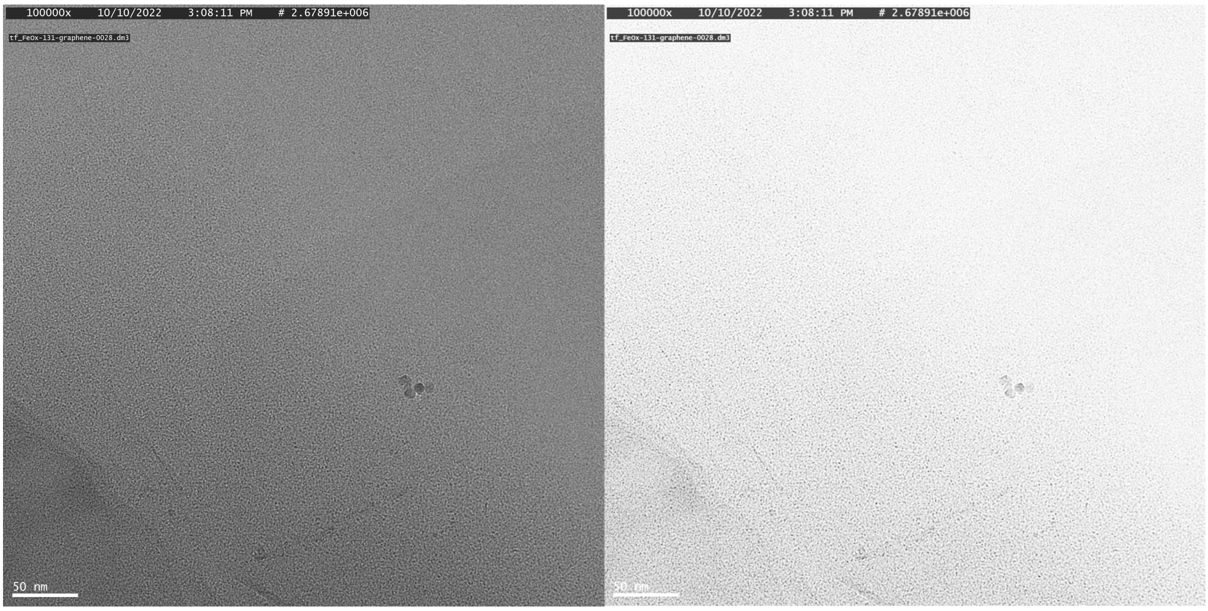

Figure S6: Transmission electron microscopy of FeO_x (batch 131, oleic acid 2:1, Acros Organics, 1 mg mL^{-1} in THF), at a magnification of 100000x; particle size by DLS 1.94±0.34 nm, higher contrast for better visibility (right).[2]

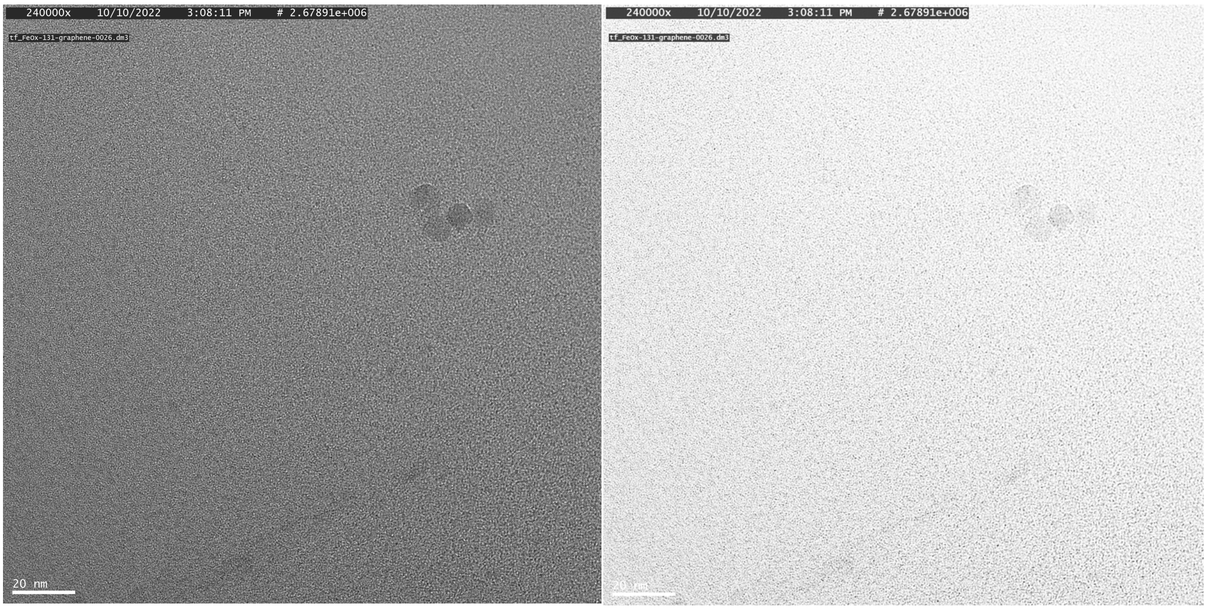

Figure S7: Transmission electron microscopy of FeO_x (batch 131, oleic acid 2:1, Acros Organics, 1 mg mL^{-1} in THF), at a magnification of 240000x; particle size by DLS 1.94±0.34 nm, higher contrast for better visibility (right).[2]

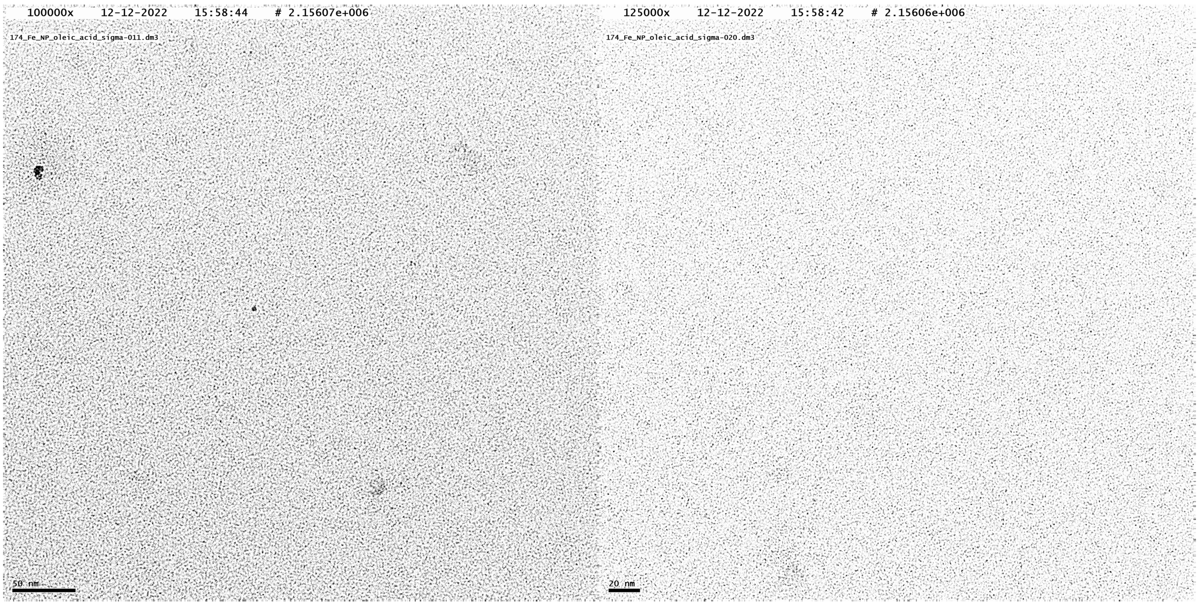

Figure S8: left) Transmission electron microscopy of FeO_x (batch 174, oleic acid 2:1, Sigma Aldrich, 1 mg mL^{-1} in THF), at a magnification of 100000x; particle size by DLS 1.94±0.34 nm, higher contrast for better visibility. right) Transmission electron microscopy of FeO_x (batch 174, oleic acid 2:1, Sigma Aldrich, 1 mg mL^{-1} in THF), at a magnification of 125000x; particle size by DLS 1.94±0.34 nm, higher contrast for better visibility.[2]

4.3 Detailed calculation of FeO_x species:

In our previous publication[2] (https://doi.org/10.1039/D3EY00256J) we evaluated the specific properties of the FeO_x NPs:

1. Through **X-Ray powder diffraction (XRD)** we established that the FeO_x photocatalyst resembles roughly Fe_3O_4 (magnetite), corresponding to x ≈ 1.33.

2. We further performed **elemental analysis as bulk technique** which revealed the core of our nanoparticles is composed of a mixed iron oxide phase, as denoted in the formulation: $(C_2H_5OH)_5/(C_{18}H_{34}O_2)_5@Fe_3O_4/FeO$.

The mixed iron oxide core of the nanoparticle formulation contains both Fe_3O_4 (a mixed-valence iron oxide composed of Fe^{2+} and Fe^{3+}) and FeO (containing only Fe^{2+}).

- FeO → contains Fe^{2+} only.
- Fe_3O_4 → is a mixed-valence compound, formally written as $FeO{\cdot}Fe_2O_3$, or containing Fe^{2+} and Fe^{3+} ions in a 1:2 ratio.

Fe_3O_4 has three iron atoms per formula unit—one Fe^{2+} and two Fe^{3+}—giving an average oxidation state of:

$$\frac{(+2)+2(+3)}{3}=\frac{8}{3}\approx +2.67$$

FeO contributes iron exclusively in the +2 oxidation state.

Assuming a 1:1 molar ratio between Fe_3O_4 and FeO (which aligns with the overall formula representation from the elemental analysis), the total iron oxidation state across the mixture becomes:

$$\frac{(3x\ Fe\ from\ Fe_3O_4\cdot\ +2.67)+(1x\ Fe\ from\ FeO\ \cdot\ +2)}{4\ total\ Fe\ atoms}=\frac{8+2}{4}=+2.5$$

Since each oxygen atom carries a -2 charge, the corresponding Fe:O ratio in the average formula FeO_x must satisfy charge neutrality. Therefore:

$$x=\frac{Average\ oxidation\ state\ of\ Fe}{2}=\frac{2.5}{2}=1.25$$

According to the elemental analysis the iron oxide composition in the nanoparticle can be reasonably represented as **$FeO_{1.25}$ (which resembles magnetite roughly as $Fe_3O_{3.75}$)**, reflecting an average oxidation state of +2.5 across the iron species.

3. To provide a surface-sensitive technique for of the oxidation state of iron within this phase, we performed **X-ray photoelectron spectroscopy (XPS) analysis** on the pure nanoparticles as well as the photoelectrodes used in the current system. The results revealed the presence of:

NPs

- Fe^{2+} = 83%
- Fe^{3+} = 17%

Photoelectrode:

- Fe^{2+} = 87.2%
- Fe^{3+} = 12.8%

From these values, we calculated the average oxidation state of Fe in the as follows:

NPs

$$Avg. oxidation\ state = (0.83\ \cdot 2) + (0.17\ \cdot 3) = 2.17$$

Photoelectrode

$$Avg. oxidation\ state = (0.872\ \cdot 2) + (0.128\ \cdot 3) = 2.128$$

Since each oxygen contributes -2 to the charge balance, the corresponding stoichiometry FeO_x is calculated by dividing the average Fe oxidation state by 2:

NPs

$$x = \frac{Average\ Fe\ oxidation\ state}{2} = \frac{2.17}{2} = 1.085$$

Photoelectrode

$$x = \frac{Average\ Fe\ oxidation\ state}{2} = \frac{2.128}{2} = 1.064$$

Thus, the iron oxide phase at the surface can be represented more precisely as **$FeO_{1.06}$** to **$FeO_{1.085}$** which reflects the predominantly Fe^{2+}-rich environment of the nanoparticles.

Furthermore, using the known Fe^{2+} and Fe^{3+} content of Fe_3O_4 (1 Fe^{2+} and 2 Fe^{3+} per formula unit) and FeO (1 Fe^{2+}), we back-calculated the relative molar ratio of Fe_3O_4 to FeO at the surface to produce the observed Fe oxidation state distribution.

Assumed:

- a = moles of Fe_3O_4
- b = moles of FeO

Fe_3O_4 contributes 1 Fe^{2+} and 2 Fe^{3+}
FeO contributes 1 Fe^{2+}

Then:

- Fe^{2+} total = a+b
- Fe^{3+} total = 2a
- Total Fe = 3a+b

Given:

$$Fe^{2+}\ fraction = \frac{a+b}{3a+b} = 0.872$$

$$Fe^{3+} fraction = \frac{2a}{3a+b} = 0.128$$

Solve using the second equation:

$$1.616a = 0.128b$$

$$\frac{a}{b} = \frac{0.128}{1.616} = 0.0792$$

Solving for this ratio yielded:

$$\frac{Fe_3O_4}{FeO} \approx 0.079 : 1\ \approx 1 : 12.6$$

This confirms that the iron oxide surface is mostly composed of FeO (92.6%) with only a minor Fe_3O_4 component (~7.4%).

4.3.1 **Summary and conclusion:**

- XPS analysis of the nanoparticles gives an Fe^{2+}:Fe^{3+} ratio of 83:17 (*published in previous study*), corresponding to x ≈ 1.085.[2]
- XPS analysis of the photoelectrode (prepared from the same material) yields an Fe^{2+}:Fe^{3+} ratio of 87.2:12.8, corresponding to x ≈ 1.06
- Bulk elemental analysis indicates a higher average Fe oxidation state, corresponding to x ≈ 1.25

These differences are consistent with the nature of the techniques used. XPS is inherently surface-sensitive, probing only the top few nanometers of the material. In contrast, bulk elemental analysis reflects the average composition of the entire sample volume, including any Fe^{3+}-rich regions in the core.

Such variation is expected and well-documented in nanoscale iron oxide systems, where surface Fe^{2+} enrichment can occur due to synthesis conditions, particle size effects, and surface ligand interactions. These factors often lead to a lower oxidation state at the surface than in the core.

We therefore represent the material as mixed-valence FeO_x (x ≈ 1.06–1.25). This range captures the mixed-valence nature of the iron oxide and reflects the true chemical state distribution across the material **FeO_x (x ≈ 1.06–1.25)**.

4.4 UV-Vis studies on FeO_x NPs suspensions

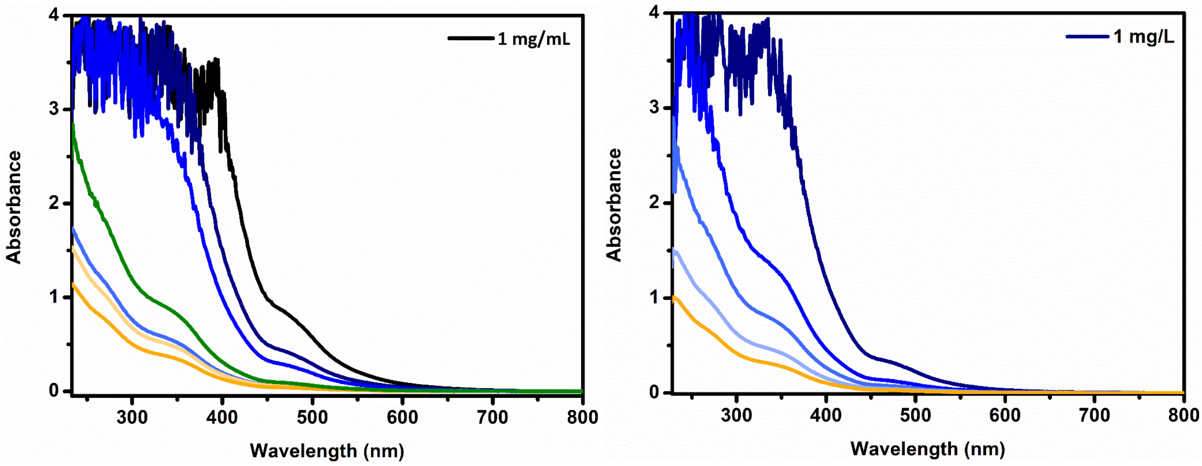

Figure S9: Absorption properties of FeO_x NPs suspensions. UV-Vis spectrum of FeO_x NPs with oleic acid (2:1, Acros Organics, batch 188) in DCM, dilutions from 1 mg mL^{-1} onwards (left). UV-Vis spectrum of FeO_x NPs with oleic acid (2:1, Sigma Aldrich, batch 189) in DCM, dilutions from 1 mg mL^{-1} onwards (right).[2]

5. Irradiation studies

5.1 Batch photocatalytic oxygen reduction

Photocatalytic oxygen reduction to hydrogen peroxide (H_2O_2) *via* FeO_x NPs was carried out in 10 mL vials, in a block of 6 slots, with irradiation of 500 mW LED from the bottom (**Figure S10**). To allow for an oxygen atmosphere and saturation of the solution, oxygen was bubbled (30 min solvent + 5 min headspace) using a needle. The temperature of the LEDs was controlled at 20°C by a liquid circulator. The photooxygenation was carried out according to the **Experimental procedure.**

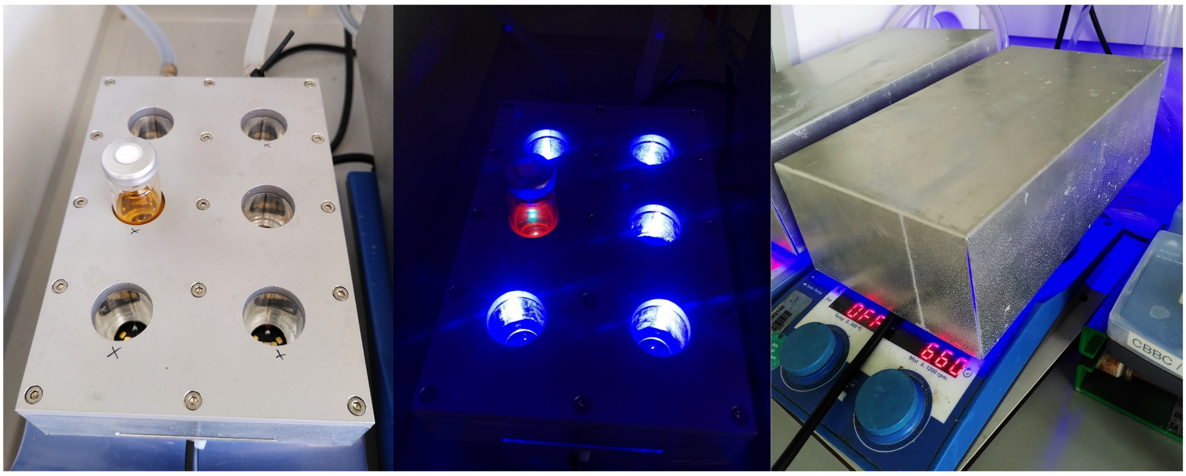

Figure S10: Batch irradiation setup for high-throughput screening.[2]

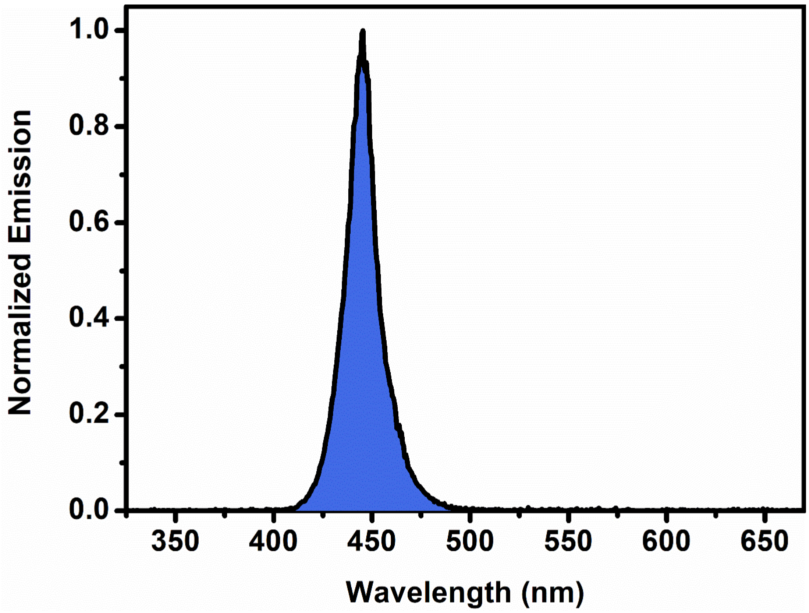

Figure S11: Normalized emission spectrum LED OSRAM Oslon SSL 80 royal blue (500 mW, λ = 445 nm, 180 mW/cm^2) as light source for batch and flow photoreactions.[2]

5.2 Screening and batch irradiation of NPs synthesized in ethanol

Iron oxide nanoparticles suspended in DCM were added to a 10 mL vial to obtain 4 mg after evaporation of DCM. Pre-oxygenated (30 minutes) Milli-Q water (4 mL) was added to the dried nanoparticles to obtain a catalyst loading of 1 mg mL^{-1}. A Teflon stirring bar was added after which the vial was closed by capping. The sample was then extensively vortexed and sonicated for 5 min for better dispersion of the nanoparticles. Finally, 5 min of oxygen bubbling through the solution was conducted to ensure an oxygen atmosphere in the vial. Irradiation studies were conducted for 5h at 20°C by 445 nm irradiation in triplicate. The photoreactions were carried out in a block of 6 slots, with irradiation of 500 mW LED (180 mW/cm^2) from the bottom. Blanks in darkness were also performed as triplicate measurements for comparison.

Catalyst recycling was performed by drying the catalyst after each irradiation reaction. The dried catalyst (~4mg) was then resuspended in DCM (0.5 mL), and stored in nitrogen atmosphere and darkness at 5°C. The method as described above could then be followed after evaporation of DCM. These catalyst recycling reactions were conducted until catalyst activity was depleted.

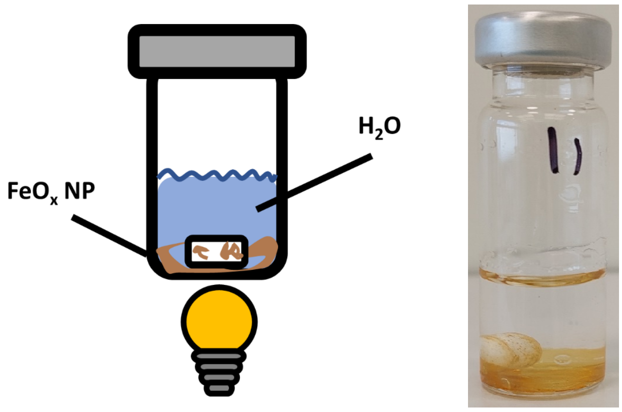

Figure S12: Left) Schematic representation of a prepared sample for irradiation studies. Right) FeO_x NPs with oleic acid (2:1) surfactant sticking to glass (batch 126).[2]

6. Quantification of hydrogen peroxide

6.1 Peroxide test strips

Peroxide test strips contain an organic redox indicator. Upon contact with peroxides the peroxide test strips produce a blue oxidation product. The peroxide concentration is measured **semiquantitatively** by visual comparison of the reaction zone of the test strip with the fields of a color scale. For accurate measuring the pH of the samples should be within the range 2-12.

Measurements were performed by immersing the test strips for one second in the samples. Excess liquid was allowed to run off and after approximately 10-15 sec a **semiquantitative** comparison was made using the color scale.

Peroxide test strips (**Figure S13**) confirm the formation of hydrogen peroxide in Milli-Q water (pH ≈ 7) after 5 h of irradiation with 445 nm light, using the same batch of FeO_x nanoparticles employed in the preparation of the photocathodes for this study. The observed blue coloration on the test strips corresponds to the characteristic response of the peroxide indicator, demonstrating the catalyst's intrinsic ability to generate H_2O_2 under illumination in the absence of additives.

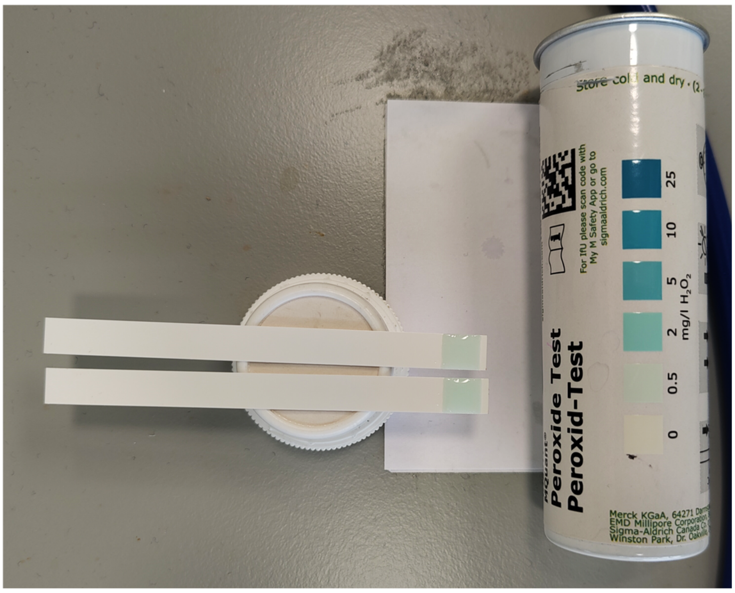

Figure S13: Semiquantitative peroxide test strip analysis of a sample irradiated with 445 nm light for 5 h in Milli-Q water (pH ≈ 7) using the same batch of FeO_x nanoparticles employed for preparing the photocathodes in the present PEC study. A clear blue coloration indicates the presence of H_2O_2, confirming the intrinsic peroxide-forming activity of the catalyst. While quantification in the bias-free PEC device was not possible due to likely Fenton degradation on FeO_x surfaces, these control data demonstrate that the catalyst is capable of producing H_2O_2 under illumination.

6.2 Iodometric titration

Reaction S1: Iodide oxidation by hydrogen peroxide.

$$H_2O_2 + H_2SO_4 + 2\,KI \rightarrow I_2 + K_2SO_4 + 2\,H_2O$$

Reaction S2: Iodometric titration.

$$I_2 + 2\,Na_2S_2O_3 \rightarrow Na_2S_4O_6 + 2\,NaI$$

It was opted for iodometric titration, because titration by potassium permanganate ($KMnO_4$) led to interference with organic matter such as the surfactants of the FeO_x photocatalyst.

6.2.1 Quantification procedure

Iodometric titration was utilized to accurately quantify the hydrogen peroxide amounts produced *via* the ORR. After uncapping the vials, they were first **semiquantitatively** analyzed *via* peroxide test strips. Subsequently, the Milli-Q water was decanted in a 20 mL vial while a magnet was held to the bottom of the initial 10 mL vial to ensure catalyst to stay in the vial. The inside of the 10 mL vial was washed once with Milli-Q water (1 mL), which was also decanted in the 20 mL vial for analysis.

Syringe filters (0.2 µm, Sartorius) were used in case solids were dispersed in the sample (*e.g.* iron oxides as additive) or when a suspension was obtained after reaction. The samples were then decanted into a syringe (5 mL) with a filter (0.2 µm) instead of decantation directly into the 20 mL vial.

For analysis potassium iodide solution (2 mL), sulfuric acid solution (1 mL) and ammonium molybdate solution (5 drops) were added to the sample. The vial was then immediately capped and stored in darkness for (exactly) 5 min. After these 5 min in darkness the solution had turned from colorless to slightly yellow. Upon addition of starch indicator, a blue/purple color was obtained, which was titrated away with 0.0001 M sodium thiosulfate solution.

The amount of potassium iodide added to each sample should be in excess. 2 mL KI (2wt%) = 236 µmol, of which 118 µmol are available for reaction with H_2O_2 (**Reaction S1**). This amount is a few orders of magnitude higher (81 – 844 times) than typical produced amounts of hydrogen peroxide (0.14 - 1.45±0.07 µmol).

7. List of photochemical H_2O_2 production values

Table S3: List of production values for the photochemical production of H_2O_2 *via* FeO_x NPs in batch.[2]

Entry	Condition	Peroxide Teststrip [mg L^{-1}]	Produced H_2O_2 [µmol L^{-1}]	Produced H_2O_2 [mmol g^{-1} L^{-1}]	Productivity H_2O_2 [mmol g^{-1} L^{-1} h^{-1}]	Normalized production H_2O_2 [mmol g^{-1} L^{-1}]
**1**	FeO_x NP Standard (4 mg, darkness)	0	0	0	0	0
**2**	FeO_x NP Standard (4 mg)	0.5 to 2	34±2	9.4±1.3	1.7±0.3	9.4±1.3
**Kinetics FeO_x NPs with oleic acid surfactant**						
**25**	1 h	0.5	10±2	2.6±1.4	2.6±1.4	2.6±1.4
**26**	2.5 h	0.5	13±2	3.9±1.0	1.6±0.4	3.9±1.0
**27**	5 h	0.5 to 2	29±2	9.0±0.4	1.8±0.1	9.0±0.4
**28**	20 h	2	36±1	14.2±1.1	0.7±0.1	14.2±1.1
**29**	67 h	2 to 5	63±13	12.8±2.5	0.2±0.1	12.8±2.5

8. Preparation of electrodes

8.1 Cleaning of FTO-coated glass

Fluorine-doped tin oxide (FTO) coated glass slides (Sigma Aldrich, 30 x 30 cm, 2.3 mm thickness, 13 Ω/sq) was cut in 10 × 10 cm pieces which were cleaned in pairs in a clean glass TLC chamber with their conductive side (marked with glass etching pen) facing each other in a V-shape. The TLC chamber was filled with Milli-Q ultra-pure water (18.2 MΩ·cm) and a teaspoon of Deconex Forte 24, then the chamber was subjected to sonication for 30 min. The Milli-Q-Deconex solution was replaced with pure Milli-Q and then with absolute EtOH, both cleaning steps included 30 min sonication. Afterwards, the plates were left to air dry with the conductive side facing upwards. Once dry, the plates were directly used for the preparation of the electrodes.

8.2 Cutting of FTO-coated glass

The cleaned FTO-coated glass was used for the preparation of the FeO_x NP and Ti-doped α-Fe_2O_3 photoelectrodes used in cyclic voltammetry (CV) measurements and for the photoelectrochemical experiments. The FTO glass was cut into pieces of 1 x 1.2 cm^2 for CV and 1.8 x 1.8 cm^2 for the (paired) photochemical experiments.

8.3 Preparation of Ti-doped α-Fe_2O_3 nanoparticle electrodes

The Ti-doped α-Fe_2O_3 nanoparticle electrodes were prepared according to an adjusted literature procedure, with optimal number of nanoparticle layers and Ti-concentration.[7] Fluorine-doped tin oxide (FTO) coated glass was pre-cleaned according to *"Cleaning of FTO-coated glass."* A 20 mL ethanol solution with 10 mM $FeCl_3$ concentration, was prepared by dissolving $FeCl_3 \cdot 6H_2O$ (54 mg, 99 %, Fisher Scientific) in 20 mL ethanol, adding 6.8 mL titanium butoxide (10% nominal Ti:Fe atomic ratio) and 70 µL concentrated HCl (37.2 wt %, Aldrich). The above solution (72 µL per cm^2 FTO, divided by 14 layers) was drop-deposited onto the pre-cleaned and cut FTO-coated glass. The dried FTO-coated glass was subsequently heated on a hotplate in air at 350 °C for 5 min to anneal. This deposition-annealing procedure is denoted as one "DA cycle". The film thickness was controlled by the number of DA cycles (14 cycles) as well as the $FeCl_3$ precursor concentration (10 mM). The as-prepared films were then further annealed in air at 550 °C for 4 h, then left to cool in the furnace until temperature reached 400°C. The electrodes were then removed from the furnace first to a 200 °C hotplate to avoid breaking, and then they were allowed to cool to room temperature. The resulting electrodes are depicted in **Figure S14**.

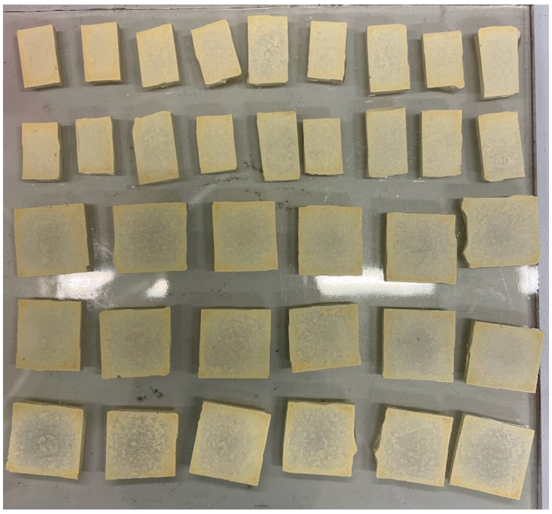

Figure S14: A picture of the Ti-doped α-Fe_2O_3 photoelectrodes.

8.4 Preparation of FeO_x nanoparticles (FeO_x NP) on FTO electrodes

The FeO_x NP electrodes were prepared according to previous literature procedure.[2] 384 µL FeO_x solution (in DCM, 13 mg/mL) was added to isopropanol (750 µL, HPLC grade) and DCM was evaporated at reduced pressure at 20 °C, resulting in an orange-brown suspension. While sonicating, Milli-Q and Nafion (50 µL, D-521 dispersion, 5% w/w in water and 1-propanol, >0.92 meq/g exchange capacity, Alfa Aesar) were added, and the mixture was sonicated for another 10 min. The FTO-coated glass was positioned with the conductive side facing upwards, and a 1 cm^2 area of the cut FTO-coated glass was left available, while the rest was covered with Scotch Magic Tape (**Figure S15**). The resulting suspension was drop-casted immediately after sonication on the exposed part of the FTO plate with a Gilson micropipette (15 µL per cm^2 FTO). The electrodes were left to airdry (for at least 1 h), after which the tape was removed using plastic tweezers to avoid scratching, resulting in the FeO_x@FTO electrode.

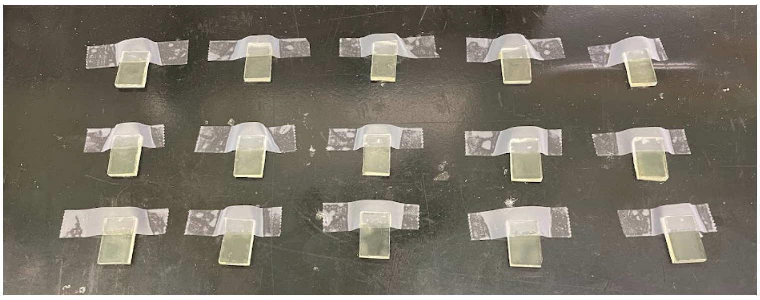

Figure S15: A picture of FeO_x@FTO electrodes airdrying before removal of the Scotch Magic Tape.

8.5 Preparation of Pt | FTO counter electrodes for photoelectrochemistry

The Pt | FTO electrodes were prepared according to the methods of Lin et. al and Bruggeman et al., *via* electrodeposition on clean FTO.[8,9] During electrodeposition, FTO was used as working electrode, Ag/AgCl (in 3 M KCl) as reference electrode (eDAQ, ET069) and Pt mesh (99.99%, 25 × 25 mm, Sigma Aldrich) as counter electrode. The electrodes were immersed in a solution of $PtCl_4$ (10 mM), 3-(2-aminoethylamino)propyl-methyldimethoxysilane (0.47 mM) and HCl (50 mM) in milli-Q, and a current of +25 mA was applied for 30 s. The electrodes turned dark metallic grey after electrodeposition and were rinsed with absolute ethanol and airdried. They were stored in a closed plastic box in ambient conditions.

9. Properties of photoelectrodes

9.1 Solid state UV-Vis spectroscopy on photoelectrodes and references

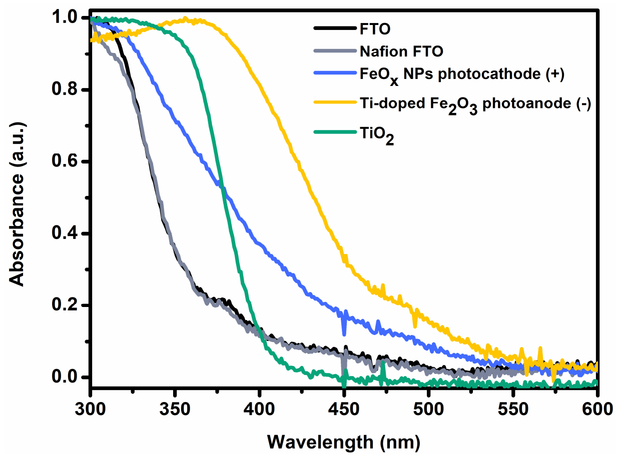

Figure S16: Absorption properties of the photoelectrodes. Solid-state UV-Vis spectroscopy of the FeO_x NPs photocathode (+) and Ti@α-Fe_2O_3 photoanode (-) in comparison to electrodes with a pure fluorine-doped tin oxide (FTO) coated glass electrode and Nafion-coated FTO. Both iron-based photoelectrodes exhibited light absorption up to 600 nm. The additional absorption band at 380 nm observed in the Ti-doped α-Fe_2O_3 photoanode is attributed to TiO_2.

9.2 Scanning electron microscopy (SEM) and energy-dispersive X-ray spectroscopy (EDX) of photoelectrodes

SEM-Energy Dispersive X-Ray Spectroscopy (EDX) elemental mapping

All samples were covered with a 20 nm carbon layer using a Leica EM ACE600 double sputter coater. Silver paint was then applied to the edges of the sample substrates to prevent charging. SEM images were captured with a Zeiss Supra 55 Scanning Electron Microscope in secondary electron detection mode, using an accelerating voltage of 3kV, a working distance of 7 mm and a 30 µm aperture. EDX elemental analysis was conducted on the same electron microscope, equipped with a 150 mm^2 Silicon Drift Detector from Oxford Instruments, at an accelerating voltage of 15 kV and a working distance of 7 mm.

10. Electrochemistry

10.1 Electrolyte solutions

For pH-dependent cyclic voltammetry measurements in an aqueous system, buffer solutions with different pH values were made using Milli-Q water. 200 mL of 50 mM NaH_2PO_4 (sodium phosphate monobasic) and 200 mL of 50 mM Na_2HPO_4 (sodium phosphate dibasic) were prepared, with respective pH values of 4.5 and 8.8. A buffer solution with pH value of 7.0 was obtained by mixing a 1:3 ratio of 50 mM NaH_2PO_4 : 50 mM Na_2HPO_4 buffer solutions, respectively. The pH 4.5 buffer solution was used as both anolyte and catholyte for the paired photoelectrochemical experiments. All pH values were measured using a SI Analytics Handylab 100 pH meter equipped with a SI Analytics pH electrode BlueLine 14 pH.

11. Cyclic voltammetry

Cyclic voltammetry measurements were performed in an undivided heart-shaped cell under N_2 or O_2 atmosphere in 3 mL electrolyte solutions which were purged for 30 min with the respective atmosphere. Since the setup was not air-tight, the measurements were carried out under gentle N_2 or O_2 flow. The cyclic voltammograms were recorded on an Autolab PGSTAT101 potentiostat (Metrohm) equipped with NOVA 2.0 software. In various measurements, the working electrode (WE) was either FTO, Nafion@FTO, the FeO_x NP catalyst on FTO or or the Ti-doped Fe_2O_3 nanoparticles on FTO. The counter electrode (CE) used was a Pt wire (diameter 0.5 mm) and a leakless Ag/AgCl (3 M KCl) was used as the reference electrode (RE; eDAQ, ET069).

The CV measurements were performed using an automated sequence setup in the NOVA 2.0 software, to maximize reproducibility. The CV measurements were performed with different scan rates and running three cycles for each scan rate. The measurements were carried out in the following specific order: the scan rates were varied from 25, 50, 75 mV/s, for three cycles in each step. During the processing of the CV data scan rate 25 mV/s and the second cycle were used, unless noted otherwise. The potential window was set at –0.2 V to 1.2 V for the Ti-doped α-Fe_2O_3 electrodes and from –0.2 V to 0.5 V for the FeO_x NP electrodes. All the CV measurements were started at 0.3 V, scanning towards anodic potential.

All CV measurements were iR drop compensated by 85%. We opted to iR compensate to correct for the voltage loss (iR drop) which is due to the resistance between the working and reference electrodes, caused by the electrolyte solution, where R is the resistance of the electrolyte solution. The uncompensated resistance of the cell was measured with the instrument built-in "current interruption (CI)" mode. The potential applied was set to -200 mV, current range to 10 µA, duration of interrupt of 2 ms, start of linear regression at 0s and end of linear regression at 500 µs, start of exponential regression at 0 s and end of exponential regression at 2 ms. The Ru linear value and the Ru exponential values were always similar, so the Ru linear value was selected as the uncompensated resistance. The iR drop was manually compensated by 85%, to ensure standardization in measurements and to avoid potentiostat oscillations and excessive correction. As partial compensation percentages can be empirical and not based on theoretical results, future studies will consider more theoretically solid methodologies for iR drop compensation.[10–12]

CV measurements were performed in the dark, under blue light (455 nm) and solar (1 sun) irradiation. For the experiments performed in the dark, the fumehood stash was covered with aluminum foil to ensure reliable control. For the experiments conducted under blue light irradiation, the following setup was employed (**Figure S17**), where the two LEDs were placed on both sides of the heart-shaped cell at 15 cm distance on each side at the same height as the cell:

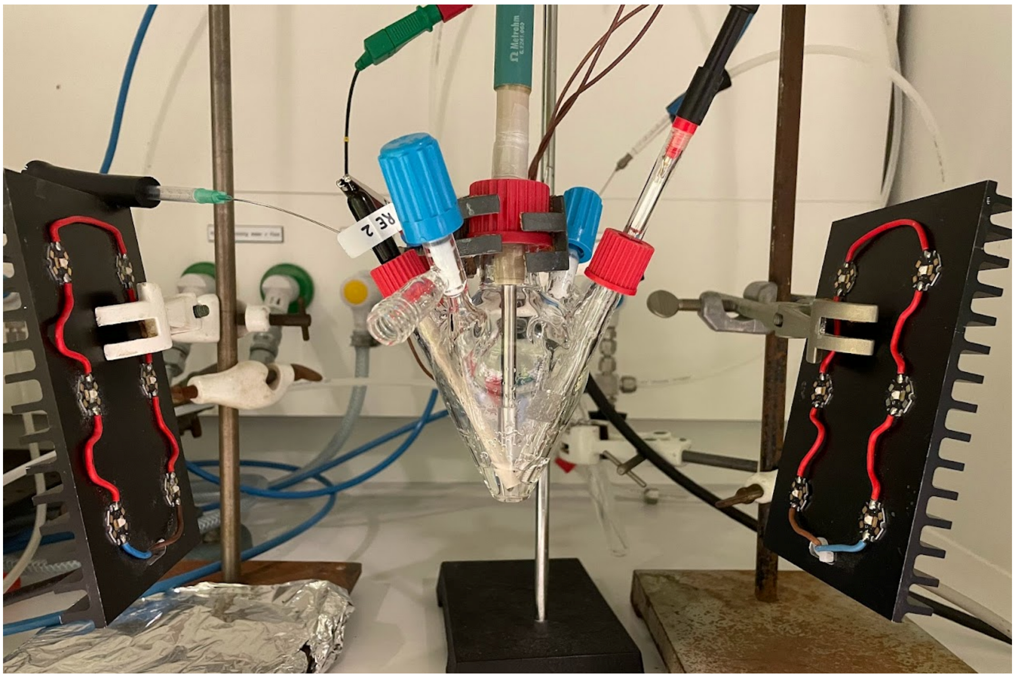

Figure S17: Irradiation setup with blue light for CV measurements.

CV measurements under solar simulator irradiation were performed in a similar fashion, however, the distance to the electrochemical cell was determined using a calibrated reference silicon solar cell and the solar simulator was placed at a distance that assured an irradiation of 1 sun (AM 1.5G) to the cell. The irradiation was only conducted from one side, where we ensured that the working electrode was fully illuminated (**Figure S18**).

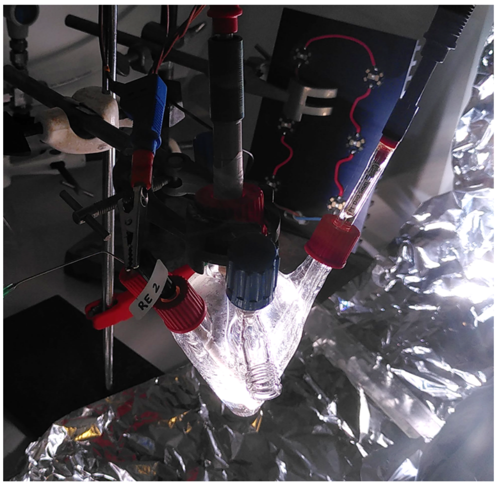

Figure S18: Heart-shaped electrochemical cell irradiated with 1 sun.

11.1 FeO_x photocathode.

All CV measurements were performed as detailed above, and all buffer solutions were prepared as previously explained.

11.1.1 Blanks with Nafion (at pH 4.5)

Electrodes for the blank experiments were prepared by drop-casting Nafion solution in the absence of FeO_x NP. All blanks were performed in pH 4.5. All blanks (grey lines in **Figure S19**) show small interaction between the Nafion polymer and the atmosphere/illumination setup for dark O_2 atmosphere (panel A) and blue O_2 atmosphere panel B) experiments, however, this interaction is less observed under solar illumination (panel C).

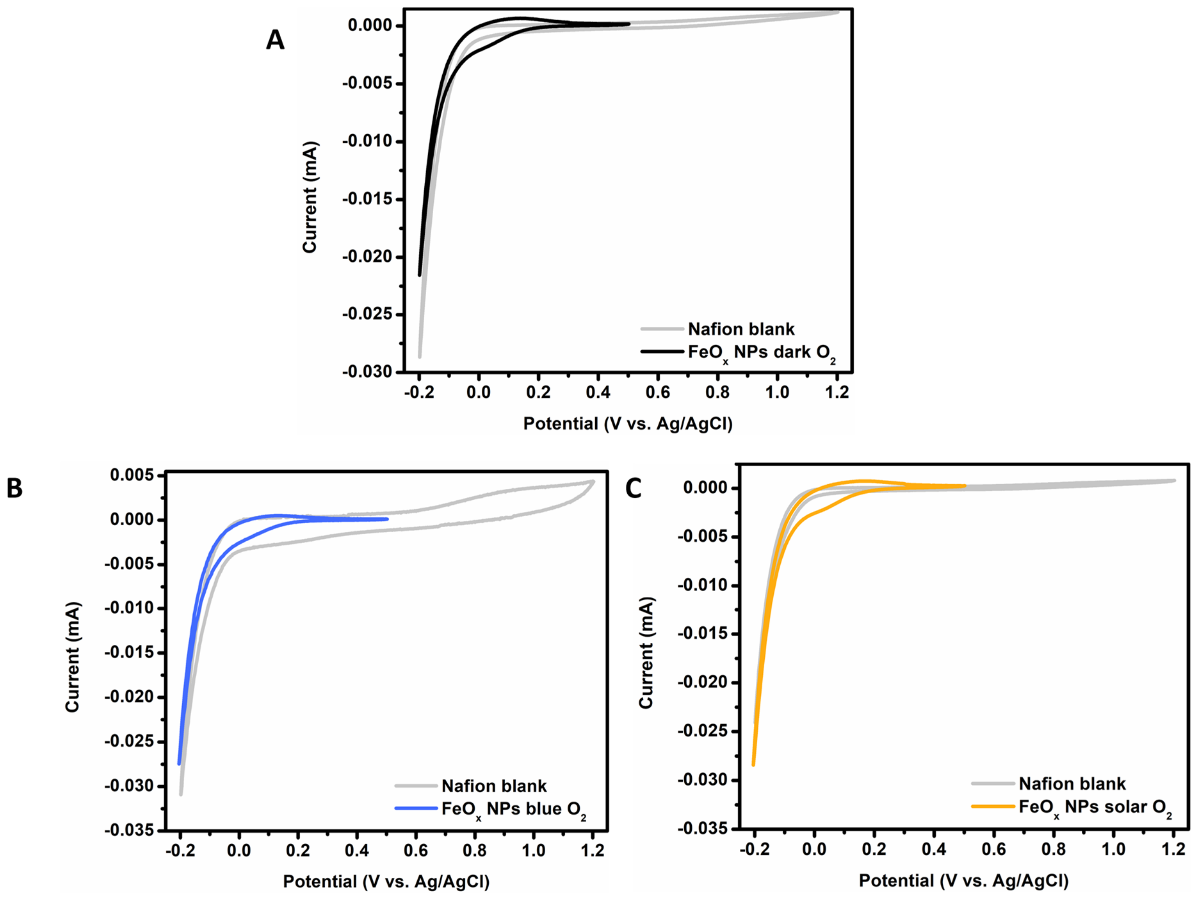

Figure S19: An overview of the blank experiments for the FeO_x photocathode (+), determined via cyclic voltammetry studies in a three-electrode setup in an undivided heart-shaped cell, using Nafion@FTO as the WE, Ag/AgCl (in 3 M KCl) as the RE, and Pt wire (d = 0.5 mm) as the CE. For all CV measurements shown we applied 0.85 x iR drop compensation. The second scans are shown, starting at 0.3 V, scanning towards anodic potential with a scan rate of 25 mV/s. Scanning range for Nafion@FTO was -0.2 V to 1.2 V. (A) Control experiment at pH 4.5 under O_2 atmosphere, in the dark. (B) Control experiment at pH 4.5 under O_2 atmosphere, under blue light irradiation. (C) Control experiment at pH 4.5 under O_2 atmosphere, under solar (1 sun) illumination.

11.1.2 Dark N_2, pH dependency

CV measurements showing pH dependency of the FeO_x@FTO photoelectrodes, demonstrate the enhanced performance of the catalyst at acidic pH even in the absence of O_2 atmosphere and irradiation (**Figure S20** green line).

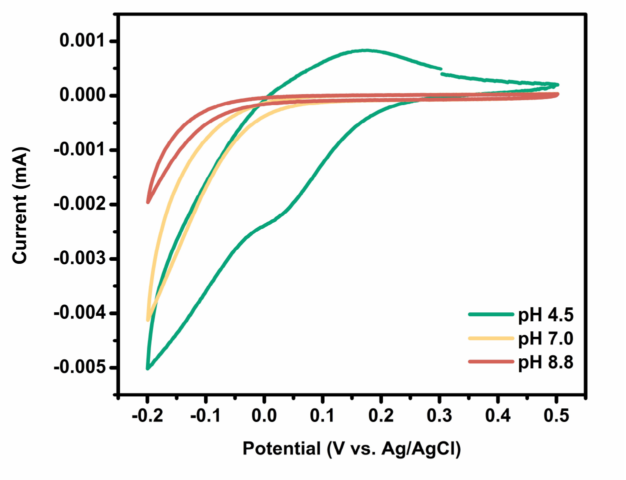

Figure S20: Cyclic voltammograms of the FeO_x NP photoelectrode under an N_2 atmosphere, in darkness, while varying the pH of the electrolyte from pH 4.5 (green) to pH 7.0 (yellow) to pH 8.8 (red). Cyclic voltammetry studies were conducted in a three-electrode setup in an undivided heart-shaped cell, using FeO_x @FTO as the WE, Ag/AgCl (in 3 M KCl) as the RE, and Pt wire (d = 0.5 mm) as the CE. For all CV measurements shown we applied 0.85 x iR drop compensation. The second scans are shown, starting at 0.3 V, scanning towards anodic potential with a scan rate of 25 mV/s. Scanning range -0.2 V to 0.5 V.

11.1.3 Dark N_2, pH 4.5 different scan rates

A lower scan rate (25 mV/s) shows more features of the reductive behavior of the FeO_x@FTO photoelectrode (*e.g* at – 0.02 V), demonstrating that performing and processing the CV measurements at 25 mV/s is more accurate than at higher scan rates.

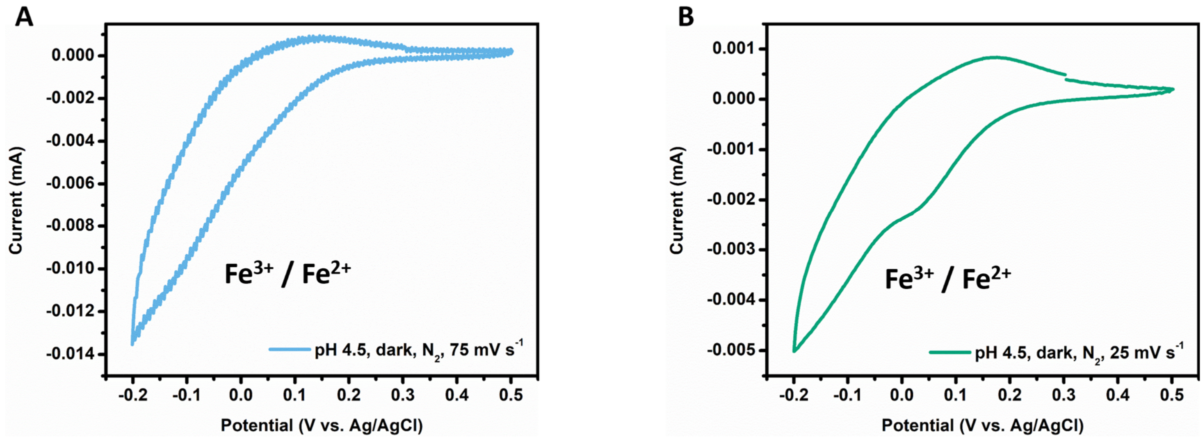

Figure S21: Cyclic voltammograms of the FeO_x NP photoelectrode under an N_2 atmosphere, in darkness, with scan rate (A) 75 mV/s or (B) 25 mV/s. Cyclic voltammetry studies were conducted in a three-electrode setup in an undivided heart-shaped cell, using FeO_x @FTO as the WE, Ag/AgCl (in 3 M KCl) as the RE, and Pt wire (d = 0.5 mm) as the CE. For all CV measurements shown we applied 0.85 x iR drop compensation. The second scans are shown, starting at 0.3 V, scanning towards anodic potential with respective scan rates. Scanning range -0.2 V to 0.5 V.

11.1.4 pH 4.5 dark, short O_2 atmosphere

Catalyst shows small activity in the absence of O_2 (**Figure S22 B**, light blue) with photocurrents of 0.014 mA/cm^2, once the electrolyte solution is briefly sparged with O_2, the catalytic activity increases with photocurrents of 0.027 mA/ cm^2. Furthermore, over several cycles in the presence of O_2 atmosphere, the catalyst stabilizes, and the electrochemical features stay constant (**Figure S22 A**).

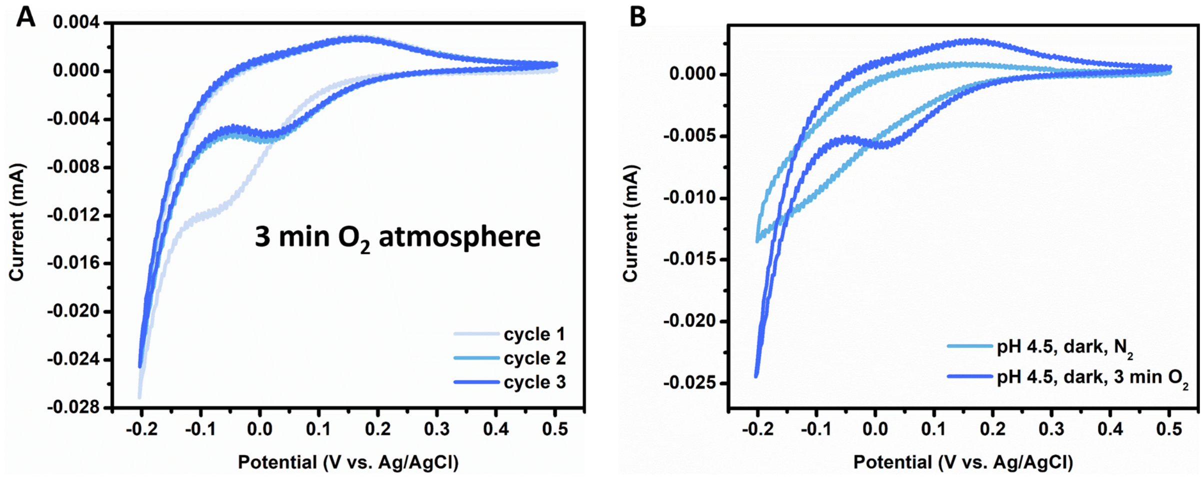

Figure S22: Cyclic voltammograms of the FeO_x NP photoelectrode under an N_2 atmosphere, in darkness, and then under short sparge (3 min) with O_2 atmosphere. Cyclic voltammetry studies were conducted in a three-electrode setup in an undivided heart-shaped cell, using FeO_x @FTO as the WE, Ag/AgCl (in 3 M KCl) as the RE, and Pt wire (d = 0.5 mm) as the CE. For all CV measurements shown we applied 0.85 x iR drop compensation. The scans are shown, starting at 0.3 V, scanning towards anodic potential with a scan rate of 25 mV/s (A) All three cycles are shown, demonstrating catalyst stabilization over several cycles. (B) Comparison of catalyst activity in N_2 atmosphere (light blue) vs. short exposure to O_2 atmosphere (dark blue), with only first scan shown.

11.2 Ti-doped α-Fe_2O_3 photoanode

11.2.1 Blanks with FTO

Electrodes for the blank experiments were prepared by cleaning and cutting FTO glass according to procedures mentioned previously. All blanks were performed in N_2 atmosphere, in darkness. All blanks (grey lines in **Figure S23**) show little interaction between the FTO glass and the different pH solutions, especially in the scanning range of interest, between -0.2 V and 1.2 V, range in which the CV experiments with Ti-doped α-Fe_2O_3 photoelectrode were conducted. The smallest interaction is observed at pH 4.5 (panel A), which is the pH chosen for the paired photoelectrochemical experiments.

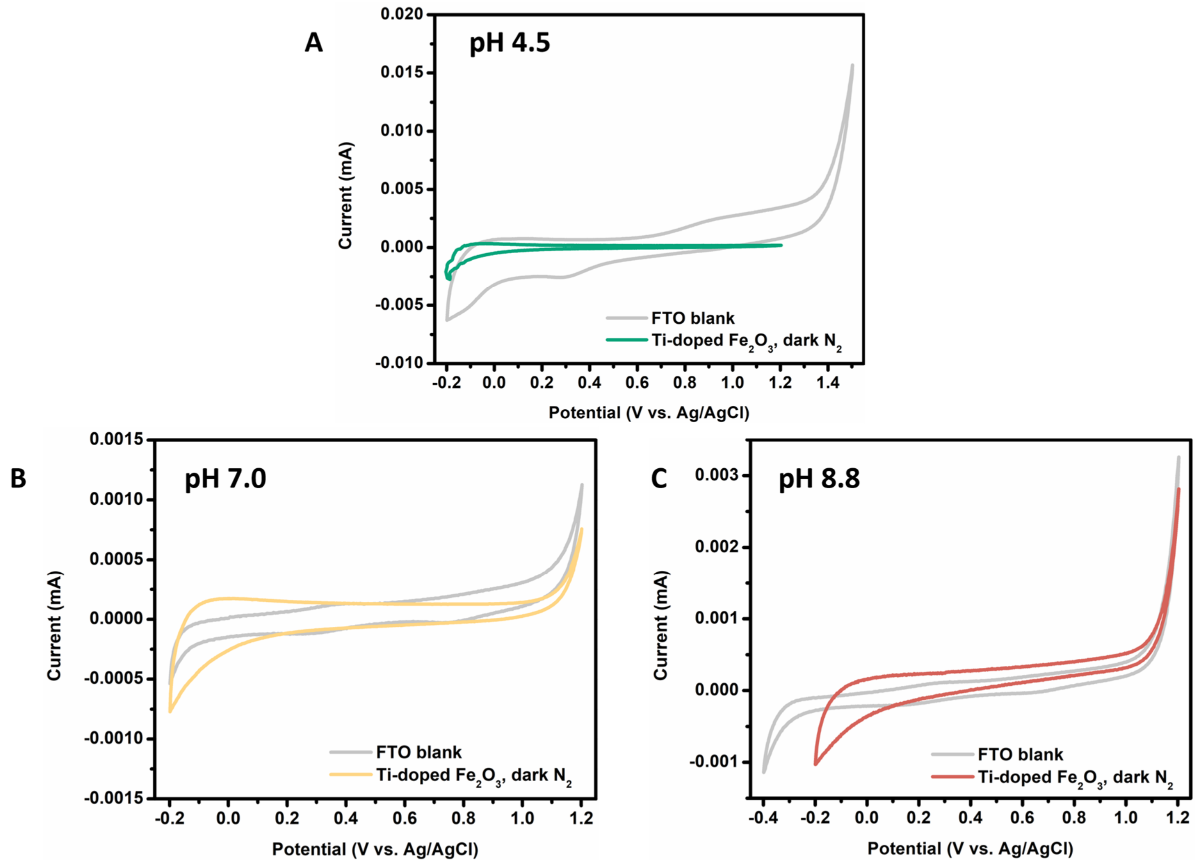

Figure S23: An overview of the blank experiments for the Ti-doped α-Fe_2O_3 photoanode (-), determined via cyclic voltammetry studies in a three-electrode setup in an undivided heart-shaped cell, using FTO glass as the WE, Ag/AgCl (in 3 M KCl) as the RE, and Pt wire (d = 0.5 mm) as the CE. For all CV measurements shown we applied 0.85 x iR drop compensation. The second scans are shown, starting at 0.3 V, scanning towards anodic potential with a scan rate of 25 mV/s. Scanning range for FTO glass blank was -0.2 V to 1.2 V. All experiments were performed in the dark, under N_2 atmosphere (A) Control experiment at pH 4.5. (B) Control experiment at pH 7.0. (C) Control experiment at pH 8.8.

11.2.2 Irradiation with blue LED

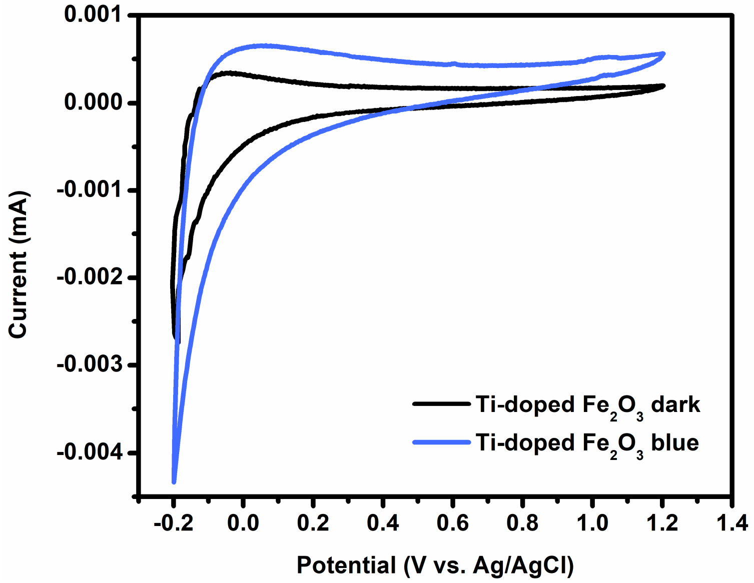

Figure S24: Comparison with darkness for enhanced WOR through blue LED, N_2, pH 4.5.

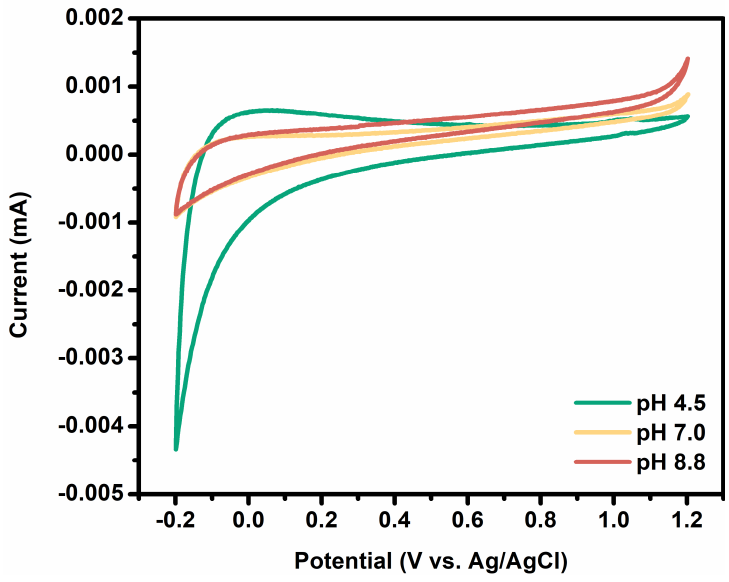

Figure S25: Cyclic voltammograms of the Ti-doped α-Fe_2O_3 photoelectrode in an N_2 atmosphere, under blue LED illumination, while varying the pH of the electrolyte from pH 4.5 (green) to pH 7.0 (yellow) to pH 8.8 (red).

11.2.3 Solar N_2, pH dependency

CV measurements showing pH dependency of the Ti-doped α-Fe_2O_3 photoelectrode demonstrate the enhanced performance of the catalyst at acidic pH even in the presence of solar (1 sun) illumination (**Figure S26** green line). While irradiation during CV measurements can introduce noise, this effect can be minimized depending on the light source and its distance to the cell (with the solar simulator performing better than blue LEDs in our case). Thus, due to irradiation taking place at the same time as the CV measurement, some noise could be observed (green line between -0.2 V and 0.3 V).

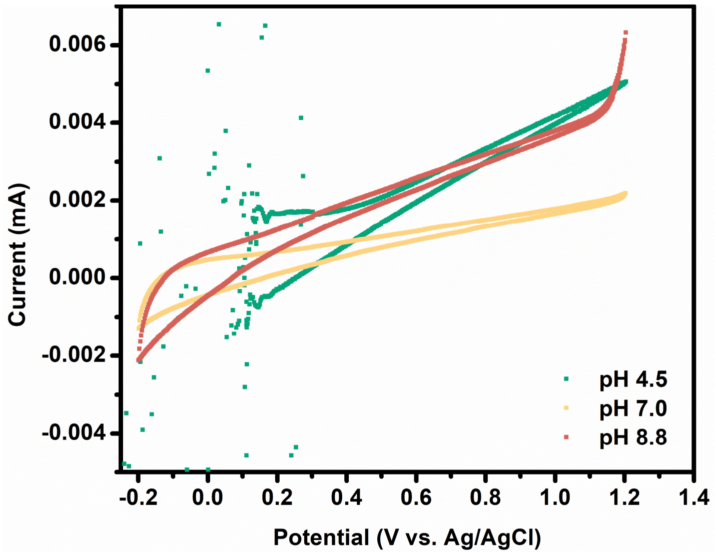

Figure S26: Cyclic voltammograms of the Ti-doped α-Fe_2O_3 photoelectrode in an N_2 atmosphere, under solar (1 sun) illumination, while varying the pH of the electrolyte from pH 4.5 (green) to pH 7.0 (yellow) to pH 8.8 (red), exhibiting noise due to illumination. Cyclic voltammetry studies were conducted in a three-electrode setup in an undivided heart-shaped cell, using Ti-doped α-Fe_2O_3 electrode as the WE, Ag/AgCl (in 3 M KCl) as the RE, and Pt wire (d = 0.5 mm) as the CE. For all CV measurements shown we applied 0.85 x iR drop compensation. The second scans are shown, starting at 0.3 V, scanning towards anodic potential with a scan rate of 25 mV/s. Scanning range -0.2 V to 1.4 V.

12. Photoelectrochemistry

12.1 General setup

A custom-made photoelectrochemical cell[9,13] was used for the paired photoelectrochemical measurements (see **Figure S27**) and it was placed on an adjustable platform to control height and distance to irradiation equipment. Electrical connection is made *via* a 3-mm thick conductive shielding gasket comprising of a polyurethane sponge core (elastomer) covered with a Ni/Cu-layer conductive fabric, that connects the FTO side of the glass plate with the steel plate of the photoelectrochemical cell that connects to the potentiostat.

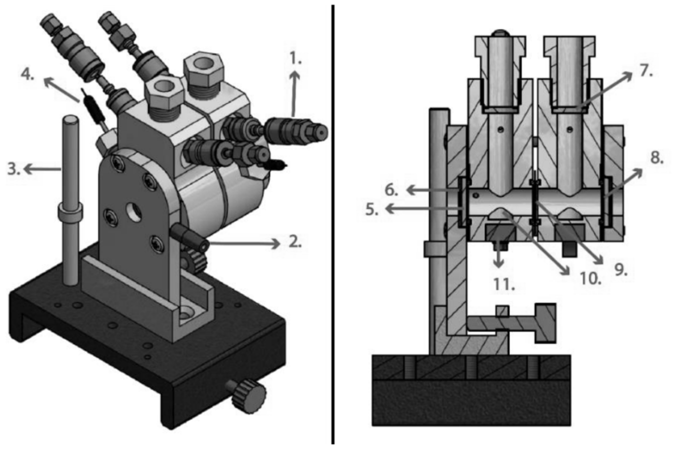

Figure S27: Schematic drawing of the dye-sensitized photoelectrochemical cell (DSPEC) device, where (1) Swagelok attachment for gas detection (not used in this study), (2) contact for connection to the potentiostat, (3) mount for photodiode sensor for light intensity detection (not used in this study), (4) reference electrode (only used in three-electrode setup, not in bias-free systems), (5) photoelectrode 1, (6) mask (0.63 cm^2), (7) septum, (8) (photo)electrode 2, (9) Nafion membrane, (10) and (11) cavities for stirring bar (not used in this study).

Paired photoelectrochemical experiments were conducted in a divided photoelectrochemical cell (PEC) with 3 mL of 50 mM NaH_2PO_4 electrolyte (pH 4.5) prepared as described above, in each compartment (see top view in **Figure S28**). A three-electrode system was employed where either Ti-doped α-Fe_2O_3 nanoparticle electrodes or FeO_x NP on FTO electrodes were used as working electrode, electrodeposited Pt | FTO was used as counter electrode and an Ag/AgCl (3 M KCl) was used as the reference electrode. The two compartments were separated by a Nafion proton exchanged membrane which was soaked in pH 7.5 buffer prior to measurements.

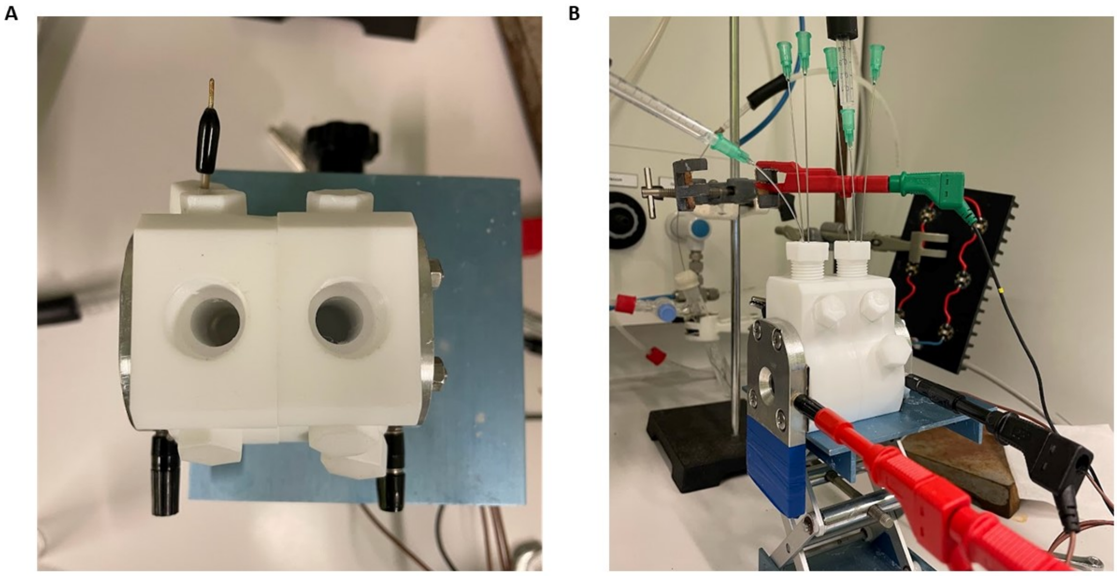

Figure S28: A. Top view photoelectrochemical cell (PEC) with two compartments. **B.** Connected PEC with N_2 and O_2 gases purging PEC compartments

Based on CV measurements, photoelectrochemical experiments were performed under irradiation of the solar simulator and the cell was placed at a 1.0 sun distance from the irradiation equipment (**Figure S33**).

12.2 Electrode assembly for photoelectrochemical cell

The Ti-doped α-Fe_2O_3 nanoparticle electrodes, FeO_x NP on FTO electrodes and Pt | FTO electrodes were cut into 1.8 x 1.8 cm^2 pieces. However, since the semiconducting materials are poorly conducting when not irradiated, and the electrical connection is made on the same to permit both conductivity and to fit the opening of the custom-made photoelectrochemical cell, a part of the Ti-doped α-Fe_2O_3 and FeO_x NP was scratched off using a scalpel, resulting in a circular spot of catalytically active material with a diameter of approximately 1 cm (**Figure S29**).

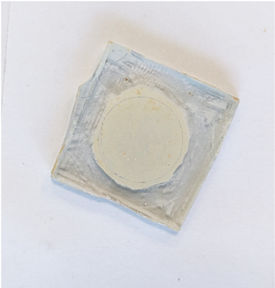

Figure S29: Example of Ti-doped α-Fe_2O_3 electrode, doctor-bladed to fit photoelectrochemical cell opening of 0.79 cm^2.

12.3 Performance photoanodes

The photoelectrochemical performance of Ti-doped α-Fe_2O_3 nanoparticle photoanodes was assessed with a three-electrode system as described above at pH 4.5 under N_2 atmosphere, with Pt wire as CE and Ag/AgCl as RE. Fresh photoanodes were used between each measurement unless otherwise stated, whereas the Pt electrode was recycled between measurements.

Chopped light voltammetry and amperometry were measured for the Ti-doped α-Fe_2O_3 as the working electrode and Pt | FTO as the counter electrode. Chopped light voltammetry data was measured from -0.2 V to 1.2 V versus Ag/AgCl. Both the working and the counter electrode were placed in pH 4.5 electrolyte solution prepared as described, where the working electrode chamber was sparged with N_2 for 10 minutes. The samples were measured in duplicate, with a scanning speed of 5 mV/s and an illumination time of 5 s was applied, alternated with 5 s of darkness. Response of the Ti-doped α-Fe_2O_3 working electrode can be found in Fig. 4C (main text).

Chopped light amperometry data was measured at 1.2 V versus Ag/AgCl in in pH 4.5 electrolyte solution prepared as described. The samples were measured in duplicate with a scanning speed of 5 mV/s and an illumination time of 10 s was applied, alternated with 10 s of darkness. The catalyst showed enhanced photocurrents of 0.02 mA/cm^2 during periods of illumination. The resulting chopped-light amperogram of the Ti-doped α-Fe_2O_3 working electrode can be found in **Figure S30**.

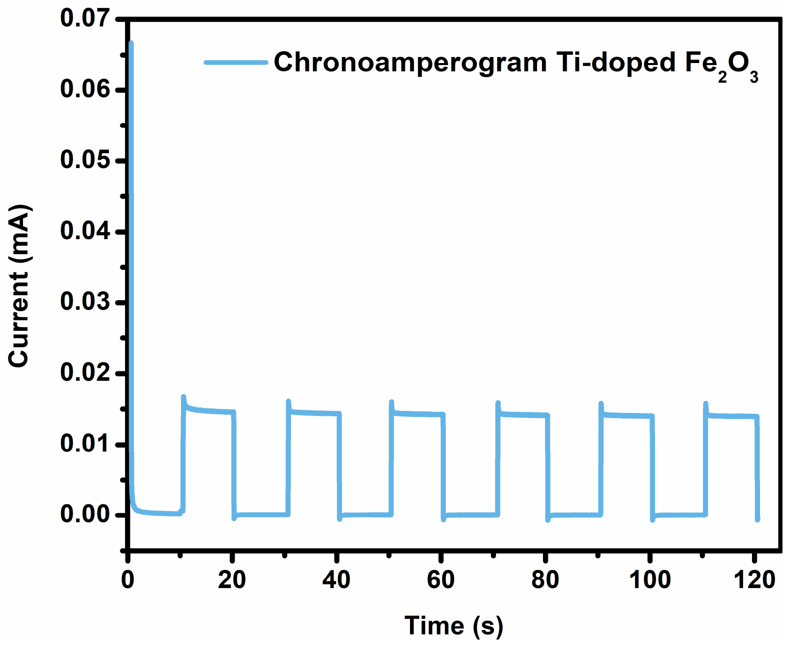

Figure S30: Chopped-light amperometry of Ti-doped α-Fe_2O_3 working electrode irradiated with a solar simulator at an illumination intensity of 1 sun for 10 s intervals, alternated with 10 s of darkness.

12.4 Performance photocathodes

The photoelectrochemical performance of FeO_x nanoparticle photocathodes was assessed with a three-electrode system as described above at pH 4.5 under O_2 atmosphere, with Pt wire as CE and Ag/AgCl as RE. Fresh photocathodes were used between each measurement unless otherwise stated, whereas the Pt electrode was recycled between measurements. The irradiation was done with a solar simulator at an illumination intensity of 1 sun.

Chopped-light voltammetry was measured for the FeO_x as the working electrode, using a Ag/AgCl (3 M KCl) reference electrode and a Pt | FTO counter electrode. Both the working and the counter electrode were in pH 4.5 electrolyte solution prepared as described earlier, where the working electrode chamber was sparged with O_2 for 10 min. The chopped-light voltammetry response was measured from –0.2 V to 0.5 V versus Ag/AgCl. The resulting chopped-light linear sweep voltammogram of the FeO_x NP working electrode is depicted in **Figure S31** and shows a photocatalytic response between +0.5 V and +0.1 V. The samples were measured in duplicate, with a scanning speed of 5 mV/s and an illumination time of 5 s was applied, alternated with 5 s of darkness.

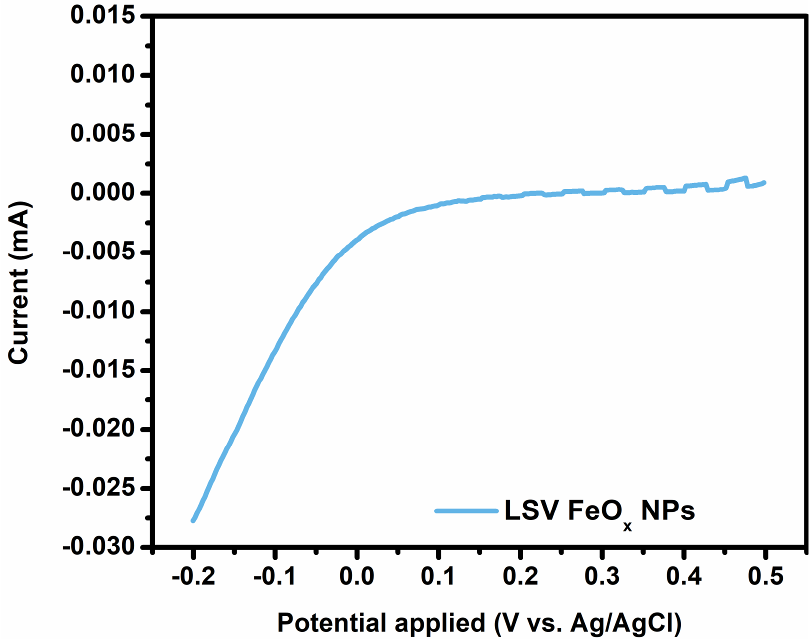

Figure S31: Chopped linear sweep voltammetry with 5 s light on/off cycles on FeO_x@FTO WE, equipped with a FTO | Pt CE and a Ag/AgCl (in 3 M KCl) RE. The sweep was carried at 5 mV/s from -0.2 V to 0.5 V in Milli-Q buffer at pH 4.5 (50 mM NaH_2PO_4). Illumination was done with solar simulator (1 sun). Below 0.1 V vs. Ag/AgCl, the absolute current is very low due to the low apparent quantum yield ($AQY_{445\ nm}$ = 0.11%) of the transparent FeO_x NP film at this loading, making light/dark contrast difficult to resolve. The FeO_x photocathode is n-type; the apparent rise in current at more positive potentials reflects a decrease in cathodic (negative) photocurrent magnitude as the overpotential for O_2 reduction diminishes, with small residual anodic currents from background and capacitive processes.

12.5 LSV integration of photocathode and photoanode

12.5.1 Methodology and Data

Step 1: Data Preparation

- Imported raw LSV datasets for:
  - **FeO_x NP photocathode:** potentials *V* (V vs. Ag/AgCl), currents *I* (mA)
  - **Ti-doped Fe_2O_3 photoanode:** potentials *V* (V vs. Ag/AgCl), currents *I* (mA)
- Both LSV data sets were recorded with positive values for currents, thus no further data modification was needed.

Step 2a: Determining the Common Potential Range

- Identified the overlapping potential range available in both datasets, ensuring consistent comparison and interpolation:
  - Start potential: **0.5 V**
  - End potential: **–0.2 V**

Step 2b: Determining the Common Potential Range – Light ON data

- ***Note:*** due to the chopped light experiment being performed by the researchers manually, there were small errors in the accurate determination of 5 seconds. Because of this, the selection of Light ON data was challenging to be separated accurately by using formulas and time stamp marks. Thus, to be able to perform the calculations below, the researchers filtered the data manually for Light ON periods. Because of the challenge to accurately estimate periods of illumination and darkness below 0.2 V, the researchers decided on the following start and end potential:
  - Start potential: **0.5 V**
  - End potential: **0.2 V**
- Established the number of interpolation points as **61**
- Calculated the potential increment ΔV as:

$$\Delta V = \frac{V_{\text{start}} - V_{\text{end}}}{N_{\text{points}} - 1} = 0.005046\,V$$

- Created a common potential axis in Excel beginning at 0.5 V, with increments of ΔV down to 0.2 V.

Step 3: Interpolating Currents at the Common Potentials

- Interpolated photocathode and photoanode currents at each point in the common potential axis using **linear interpolation** implemented by Excel's `FORECAST.LINEAR()` function:

For example, for photocathode current interpolation at common potential V_c:

```
I_photocathode(Vc) = FORECAST.LINEAR(Vc; Range Photocathode Currents; Range Photocathode Potentials)
```

- Applied the same to Ti-doped FeOx photoanode data:

```
I_photoanode(Vc) = FORECAST.LINEAR(Vc; Range Photoanode Currents; Range Photoanode Potentials)
```

Step 4: Calculating the Current Difference at Each Potential

- Determined the current difference between electrodes for each interpolated potential point:

$$\Delta I(V_c) = I_{\text{photocathode}}(V_c) - I_{\text{photoanode}}(V_c)$$

- In Excel, this corresponds to: `= Photocathode_Current - Photoanode_Current`

- Searched the column of ΔI for the sign change from negative to positive in our case, indicating the potential interval $[V_1, V_2]$ containing the bias-free intersection.

Step 5: Finding the Intersection Potential $V_{\text{intersection}}$

- Used linear interpolation between potential points V_1 and V_2 with corresponding current differences $D_1 = \Delta I\ (V_1)$ and $D_2 = \Delta I\ (V_2)$ to find the zero-crossing potential:

$$V_{\text{intersection}} = V_1 - D_1 \times \frac{V_2 - V_1}{D_2 - D_1}$$

- Excel formula example, assuming values in rows i and $i + 1$: `= Ai - Gi * (A{i+1} - Ai) / (G{i+1} - Gi)`

- **Calculated intersection potential:** $V_{\text{intersection}} = 0.208672355$ V

Step 6: Interpolating Operating Current at $V_{\text{intersection}}$

- For each electrode, found the operating current at the intersection potential by linear interpolation between current values I_1 and I_2 at potentials V_1 and V_2:

$$I_{\text{intersection}} = I_1 + (V_{\text{intersection}} - V_1) \times \frac{I_2 - I_1}{V_2 - V_1}$$

- Excel formula example:

```
= I1 + (Vintersection - V1) * (I2 - I1) / (V2 - V1)
```

- **Calculated operating currents:**

Electrode	$I_{\text{intersection}}$ (mA)
FeO_x NP Photocathode	-1.21×10^{-4}
Ti-doped Fe_2O_3 Photoanode	-1.21×10^{-4}

Step 7: Confirmation of Interpolation Accuracy

- The interpolated intersection currents for the photocathode and photoanode differ by:

$$\left|I_{\text{photocathode}} - I_{\text{photoanode}}\right| = 0 \text{ mA}$$

- The matched photocurrents confirm the accuracy of the calculated intersection potential and operating current.

Table S4: Interpolated linear sweep voltammetry current data for FeO_x NP photocathode and Ti-doped Fe_2O_3 photoanode with respective calculated common potential values and current difference values to visualize the crossing zero point.

Calculated Common Potential (V)	Interpolated Current FeO_x NP Photocathode (mA)	Interpolated Current Ti-doped Fe_2O_3 Photoanode (mA)	Current difference (mA)
0,501708984	0,001171631	0,003937103	-0,002765472
0,496663411	0,001149368	0,003867223	-0,002717855
0,491617839	0,001127104	0,003797343	-0,002670239
0,486572266	0,00110484	0,003727463	-0,002622622
0,481526693	0,001082577	0,003657582	-0,002575006
0,47648112	0,001060313	0,003587702	-0,002527389
0,471435547	0,001038049	0,003517822	-0,002479773
0,466389974	0,001015786	0,003447942	-0,002432156
0,461344401	0,000993522	0,003378062	-0,00238454
0,456298828	0,000971259	0,003308182	-0,002336923
0,451253255	0,000948995	0,003238301	-0,002289306
0,446207682	0,000926731	0,003168421	-0,00224169
0,441162109	0,000904468	0,003098541	-0,002194073
0,436116536	0,000882204	0,003028661	-0,002146457
0,431070964	0,000859941	0,002958781	-0,00209884
0,426025391	0,000837677	0,002888901	-0,002051224
0,420979818	0,000815413	0,002819021	-0,002003607
0,415934245	0,00079315	0,00274914	-0,001955991
0,410888672	0,000770886	0,00267926	-0,001908374
0,405843099	0,000748623	0,00260938	-0,001860758
0,400797526	0,000726359	0,0025395	-0,001813141
0,395751953	0,000704095	0,00246962	-0,001765525
0,39070638	0,000681832	0,00239974	-0,001717908
0,385660807	0,000659568	0,00232986	-0,001670291
0,380615234	0,000637304	0,002259979	-0,001622675
0,375569661	0,000615041	0,002190099	-0,001575058
0,370524089	0,000592777	0,002120219	-0,001527442
0,365478516	0,000570514	0,002050339	-0,001479825
0,360432943	0,00054825	0,001980459	-0,001432209
0,35538737	0,000525986	0,001910579	-0,001384592
0,350341797	0,000503723	0,001840698	-0,001336976
0,345296224	0,000481459	0,001770818	-0,001289359
0,340250651	0,000459196	0,001700938	-0,001241743
0,335205078	0,000436932	0,001631058	-0,001194126
0,330159505	0,000414668	0,001561178	-0,00114651
0,325113932	0,000392405	0,001491298	-0,001098893
0,320068359	0,000370141	0,001421418	-0,001051276
0,315022786	0,000347878	0,001351537	-0,00100366
0,309977214	0,000325614	0,001281657	-0,000956043
0,304931641	0,00030335	0,001211777	-0,000908427

0,299886068	0,000281087	0,001141897	-0,00086081
0,294840495	0,000258823	0,001072017	-0,000813194
0,289794922	0,000236559	0,001002137	-0,000765577
0,284749349	0,000214296	0,000932257	-0,000717961
0,279703776	0,000192032	0,000862376	-0,000670344
0,274658203	0,000169769	0,000792496	-0,000622728
0,26961263	0,000147505	0,000722616	-0,000575111
0,264567057	0,000125241	0,000652736	-0,000527495
0,259521484	0,000102978	0,000582856	-0,000479878
0,254475911	8,07142E-05	0,000512976	-0,000432261
0,249430339	5,84506E-05	0,000443096	-0,000384645
0,244384766	3,6187E-05	0,000373215	-0,000337028
0,239339193	1,39234E-05	0,000303335	-0,000289412
0,23429362	-8,34025E-06	0,000233455	-0,000241795
0,229248047	-3,06039E-05	0,000163575	-0,000194179
0,224202474	-5,28675E-05	9,36948E-05	-0,000146562
0,219156901	-7,51311E-05	2,38146E-05	-9,89457E-05
0,214111328	-9,73947E-05	-4,60655E-05	-5,13292E-05
**0,209065755**	**-0,000119658**	**-0,000115946**	**-3,71264E-06**
**0,204020182**	**-0,000141922**	**-0,000185826**	**4,39039E-05**

12.5.2 Notes about methodology

1. The calculations were only performed on data acquired during illumination periods of the chopped-light LSV experiments.
2. The above calculations were performed on a subset of the data points acquired during the illumination periods in the chopped LSV data. The main reason for this was to avoid human-error in determining the illumination and darkness periods, as these data points were not automatically marked during data acquisition as the chopped experiment was performed manually.
3. The potential range used in the calculations (0.5 V to 0.2 V) is narrower than the common potential window (0.5 V to -0.2 V) between the photocathode and photoanode LSV data sets, to avoid additional human bias in the choice of the data points.
4. The intersection potential ($V_{intersection}$) value was determined between the last two data points in the curated data set. In the future, the researchers would change the methodology for chopped LSV data acquisition, by automating the periods of illumination and darkness, such that data filtering to be more accurate and to avoid human error associated with short time intervals (5 seconds in our case).

12.5.3 Conclusion

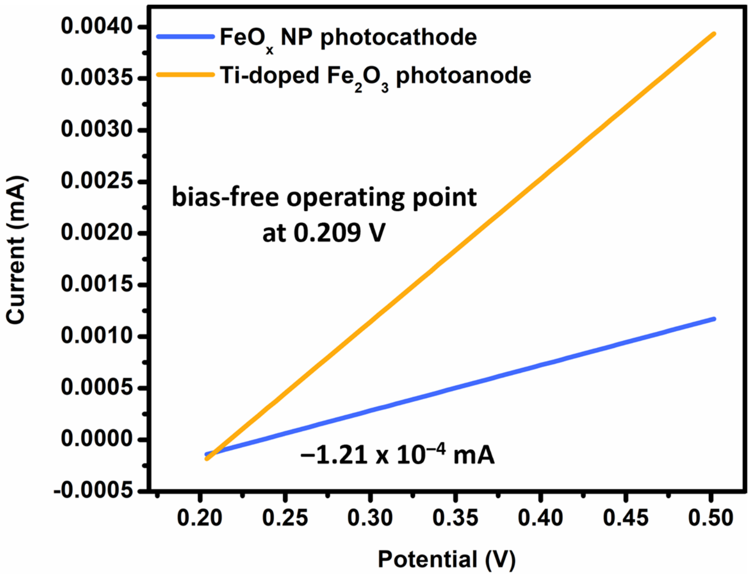

Figure S32: Intersection of interpolated linear sweep voltammetry (LSV) current data of the FeO_x NP photocathode (blue) and Ti-doped Fe_2O_3 photoanode (orange) plotted against a common potential axis calculated from LSV potential data recorded vs. Ag/AgCl as reference electrode.

- The FeOx NP photocathode and Ti-doped Fe_2O_3 photoanode LSV curves intersect at approximately **0.209 V**, delivering matched photocurrents of approximately $\mathbf{-1.21 \times 10^{-4}}$ **mA.**
- This intersection represents a **bias-free operating point**, meaning the tandem photoelectrochemical system generates net photocurrent under illumination without any externally applied voltage.
- The negative sign of the current reflects the cathodic process at the photocathode.

12.6 Paired bias-free photoelectrochemistry experiments

The photoelectrochemical performances of both photoanodes (Ti-doped α-Fe_2O_3 nanoparticle electrodes) and photocathodes (FeO_x NP on FTO electrodes) were studied in a three-electrode system using a 3 mL electrolyte solution of pH 4.5 in each chamber of the photoelectrochemical cell, in the absence of a reference electrode to ensure bias-free conditions. The anolyte solution was purged with N_2 for 10 min, while the catholyte solution was purged with O_2 for 10 min. The N_2 and O_2 respectively were kept at a low flow during the irradiation experiments to maintain the respective atmospheres. The irradiation experiments were conducted from both the photoanode and the photocathode side, with 10 s of illumination alternated with 10 s of darkness using the illumination setup depicted in **Figure S33**.

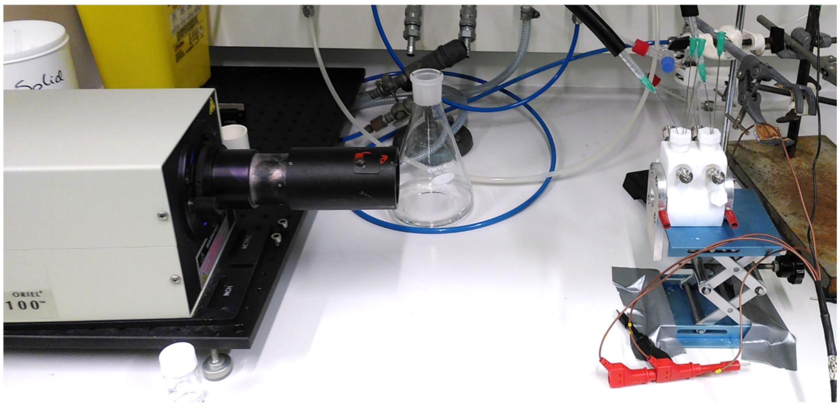

Figure S33: Setup of solar simulator at 1 sun distance from the photoelectrochemical cell (prior to irradiation experiments).

The irradiation experiments were conducted in a bias-free two-electrode setup and chopped light amperometry at 0 V demonstrated a difference in photocurrent depending on the illumination side. When illuminated from the FeO_x NP side, there is very little photocurrent observed (-0.12 μA/cm^2). When the cell was illuminated from the Ti-doped Fe_2O_3 side, we observe an enhanced photocurrent of 0.8 μA/cm^2.

12.7 Detailed thermodynamic analysis and mechanism

12.7.1 pH referencing and RHE conversion

All band edge potentials and redox potentials are referenced to the RHE scale, which accounts for pH via:

$$E_{RHE} = E_{NHE} + 0.059 \cdot pH$$

This ensures that oxidation and reduction potentials can be compared directly for both photoelectrodes in the same electrolyte.

12.7.2 Thermodynamic potentials at pH 4.5

1. Oxygen evolution reaction (OER):

$$H_2O \rightarrow O_2 + 4H^+ + 4e^-, \qquad E^0_{NHE} = +1.23\ V$$

Shifted to pH 4.5:

$$E_{RHE} = 1.23 + (0.059 \cdot 4.5) = +1.50\ V$$

2. Direct two-electron oxygen reduction to H_2O_2 (ORR):

$$O_2 + 2H^+ + 2e^- \rightarrow H_2O_2, \qquad E^0_{NHE} = +0.68\ V$$

Shifted to pH 4.5:

$$E_{RHE} = 0.70 + (0.059 \cdot 4.5) = +0.95\ V$$

3. Indirect stepwise one-electron oxygen reduction (ORR):

$$O_2 + e^- \rightarrow O_2^{\cdot-}, \quad E^0_{NHE} = -0.33\ V \qquad \text{and} \qquad O_2^{\cdot-} + e^- + 2H^+ \rightarrow H_2O_2, \ E^0_{NHE} = +1.44\ V$$

Shifted to pH 4.5:

$$E_{RHE} = -0.33 + (0.059 \cdot 4.5) = -0.06\ V \qquad \text{and} \qquad E_{RHE} = +1.44 + (0.059 \cdot 4.5) = -1.71\ V$$

Note: The intermediate $O_2^{\bullet-}$ is in protonation equilibrium with $HO_2^{\bullet}$.

$$O_2^{\cdot-} + H^+ \rightleftharpoons HO_2^{\cdot}, \quad pK_a \approx 4.8$$

Thus, at pH 4.5, roughly half the intermediate will be in the protonated radical form. However, the thermodynamic potentials given above already account for the proton-coupled nature of the second step and represent the net driving force for H_2O_2 formation from $O_2{\bullet}^-$ under acidic conditions.

12.7.3 Band edge positions at pH 4.5

The CB and VB positions were determined from Mott–Schottky analysis and optical band gaps (α-Fe_2O_3: Wang et al., *Nano Lett.* 2011;[7] FeO_x: Freese et al., *EES Catalysis* 2024[2]), with pH adjustments to match the measurement conditions of this study:

Material	CB (V vs. RHE)	VB (V vs. RHE)	E_g (eV)	Role
Ti-doped α-Fe_2O_3	+0.10	+2.30	2.20	Water oxidation
FeO_x (x ≈ 1.06–1.25)	–0.07	+2.75	2.82	O_2 reduction to H_2O_2

12.7.4 Thermodynamic feasibility

• Water oxidation (photoanode):

The valence band of α-Fe_2O_3 (VB = +2.30 V) is substantially more positive than the O_2/H_2O redox potential (+1.50 V), providing a strong thermodynamic driving force for the oxygen evolution reaction (OER).

• Indirect $1e^-$ oxygen reduction (photocathode — main mechanism):

The conduction band of FeO_x (CB = −0.07 V) is 10 mV more negative than the $O_2/O_2^{\bullet-}$ potential (−0.06 V), corresponding to a nominal driving force of $\Delta E \approx +0.01$ V ($|\Delta G| \approx 1$ kJ mol^{-1}). While this driving force is small, it is thermodynamically favorable, and—as discussed in our previous publication—this pathway is proposed as the primary oxygen reduction route for our FeO_x NP system.

• Direct $2e^-$ oxygen reduction (photocathode — secondary pathway):

The FeO_x conduction band (−0.07 V) lies 1.02 V more negative than the O_2/H_2O_2 potential (+0.95 V), indicating a large driving force for the $2e^-$ pathway. Nevertheless, experimental evidence suggests this process contributes less significantly than the $1e^-$ route.

12.7.5 Complete electron/charge flow in the α-Fe_2O_3/FeO_x Z-scheme at pH 4.5 (vs. RHE)

1. **Photoexcitation of Ti-doped α-Fe_2O_3 (photoanode):**

   Absorption of a photon promotes an electron from the valence band (VB, +2.30 V) to the conduction band (CB, +0.10 V), leaving a photogenerated hole in the VB.

2. **Photoexcitation of FeO_x nanoparticles (photocathode):**

   Absorption of a photon promotes an electron from the VB (+2.75 V) to the CB (–0.07 V), leaving a photogenerated hole in the VB.

3. **Z-scheme interfacial recombination:**

   Electrons in the CB of α-Fe_2O_3 recombine with holes in the VB of FeO_x across the electrical contact/junction, leaving behind oxidative holes in α-Fe_2O_3 and reductive electrons in FeO_x.

4. **Oxidation half-reaction (photoanode):**

   Holes in α-Fe_2O_3 (VB, +2.30 V) oxidize water to O_2 at +1.50 V:

   $$H_2O \rightarrow O_2 + 4H^+ + 4e^-, \qquad E^0_{NHE} = +1.50\,V$$

5. **Photocathode reduction half-reaction, one-electron pathway (main):**

   Electrons in FeO_x (CB, –0.07 V) reduce dissolved O_2 to superoxide:

   $$O_2 + e^- \rightarrow O_2^{\cdot -}, \quad E^0_{RHE} = -0.06\,V$$

   The $O_2^{\bullet -}$ intermediate, in equilibrium with its protonated form $HO_2^{\bullet}$ ($pK_a \approx 4.8$), undergoes further reduction and protonation to form H_2O_2:

   $$O_2^{\cdot -} + e^- + 2H^+ \rightarrow H_2O_2, \qquad E^0_{RHE} = +1.71\,V$$

   This second step may proceed via surface-bound or solution-phase routes, with disproportionation of $HO_2\bullet$ also contributing to H_2O_2 formation.

6. **Photocathode reduction half reaction, direct two-electron pathway (secondary)**

   $$O_2 + 2H^+ + 2e^- \rightarrow H_2O_2, \qquad E^0_{RHE} = +0.95\,V$$

7. **Driving force considerations:**

   The first $1e^-$ $O_2 \rightarrow O_2\bullet^-$ step operates close to thermodynamic equilibrium ($\Delta E \approx 10$ mV), making it kinetically limiting and consistent with the observed modest photocurrents (0.8 → 0.3 μA cm^{-2}). Water oxidation at the photoanode remains strongly driven under these conditions.

12.7.6 Z-scheme operation

In the Z-scheme, photogenerated electrons in α-Fe_2O_3's CB transfer to FeO_x's VB, recombining with holes there. This leaves strong oxidative holes in α-Fe_2O_3 for OER, and strong reductive electrons in FeO_x for the e^- ORR, allowing overall water-to-H_2O_2 conversion without external bias.

12.7.7 Implication for the observed small photocurrent (0.8 → 0.3 μA cm^{-2})

The FeO_x CB provides only a marginal overpotential for the first $1e^-$ step ($O_2 + e^- \rightarrow O_2{\bullet}^-$). Even though the reaction is thermodynamically allowed, such a tiny driving force typically yields slow interfacial electron-transfer kinetics. The second step, however, is strongly exergonic relative to the FeO_x CB, so the overall kinetics are likely dictated by the rate of the initial one-electron O_2 activation. Additional kinetic penalties can arise from (i) adsorption/activation of O_2 at the FeO_x surface, (ii) surface states and ligand shells, and (iii) O_2 mass transport at low current density. Hence the μA cm^{-2} currents are consistent with a rate-limiting $1e^-$ ORR step rather than a fundamental thermodynamic shortfall.

13. XPS Analysis

X-ray photoelectron spectroscopy (XPS) was performed using a Surface Science Instruments SSX-100 ESCA spectrometer, equipped with a monochromatic Al Kα X-ray source (hν =1486.6 eV). The pressure in the measurement chamber was maintained below $5*x10^{-9}$ mbar during data acquisition. The photoelectron take-off angle was 37° with respect to the surface normal. The diameter of the analyzed area was 1000 µm; the energy resolution was 1.26 eV (or 1.67 eV for a broad survey scan). XPS spectra were analyzed using the least-squares curve fitting program Winspec, developed at the LISE, University of Namur, Belgium and included a Shirley baseline subtraction and a peak deconvolution using a linear combination of Gaussian and Lorentzian functions, taking into account the experimental resolution. The spectra were fitted with a minimum number of peaks consistent with the structure of the surface. Binding energies of isolated peaks are given ± 0.05 eV; when more than one component was needed to reproduce the raw data, the error in peak position was ± 0.1 eV. Binding energies were referenced to the C 1s photoemission peak originating from adventitious carbon (C-C/C=C) at a binding energy of 284.8 eV.

FeO_x NP on FTO electrodes (including Nafion, see preparation above) were measured directly after preparation (sample before catalysis) or after its use in the photoelectrochemical cell (after catalysis). Both electrodes were compared to reference FTO electrodes coated with Nafion. Charge neutralization was achieved with a flood gun set to 0 eV and a molybdenum grid placed 3 mm above the sample.

Measurements of Fe 2*p*, C 1*s* and O 1*s* core level spectra of the FeO_x NPs photocathode (before and after catalysis, compared with reference material) are depicted in in **Figure S34** to **Figure S36**. The analysis of the Fe 2*p* core level spectrum indicates successful application of the FeO_x NPs as photocatalyst (Fe^{2+} component is peaked at a binding energy of 711.7 eV and the Fe^{3+} component at 714.3 eV). The overall signal-to-noise ratio decreased post-catalysis, possibly due to nanoparticle leaching, which is indicated in the C 1*s* and the O 1*s* spectra through increased presence of reference material (*i.e.* FTO electrode and Nafion), deduced from the higher relative spectral-intensity of the corresponding peaks at *e.g.* 292.0 eV being the $C\text{-}F_2$ component.

Generally, the active sites of the FeO_x NPs were successfully replenished through the photoelectrochemical reactions occurring on the photocathode. Notably, the FeO_x NPs photocathode remained stable during catalysis, as evidenced by CV and XPS analyses.

Notably, the FeO_x NPs photocathode remained stable during catalysis, as evidenced by CV and XPS analyses. In our previous study, FeO_x NPs prior to catalysis exhibited an Fe^{2+}/Fe^{3+} ratio of 83% and 17%, respectively, which shifted to 54% Fe^{2+} and 46% Fe^{3+} upon oxidation during deactivation.[2] In this study, the application and regeneration of FeO_x NPs within the photoelectrode system maintained a stable catalytic state, with the Fe^{2+}/Fe^{3+} ratio changing only slightly from 87.2% / 12.8% before catalysis to 83.1% / 16.9% after catalysis. Additionally, the FeO_x NPs photocathode remained stable for at least six months without signs of aging during storage in the dark.

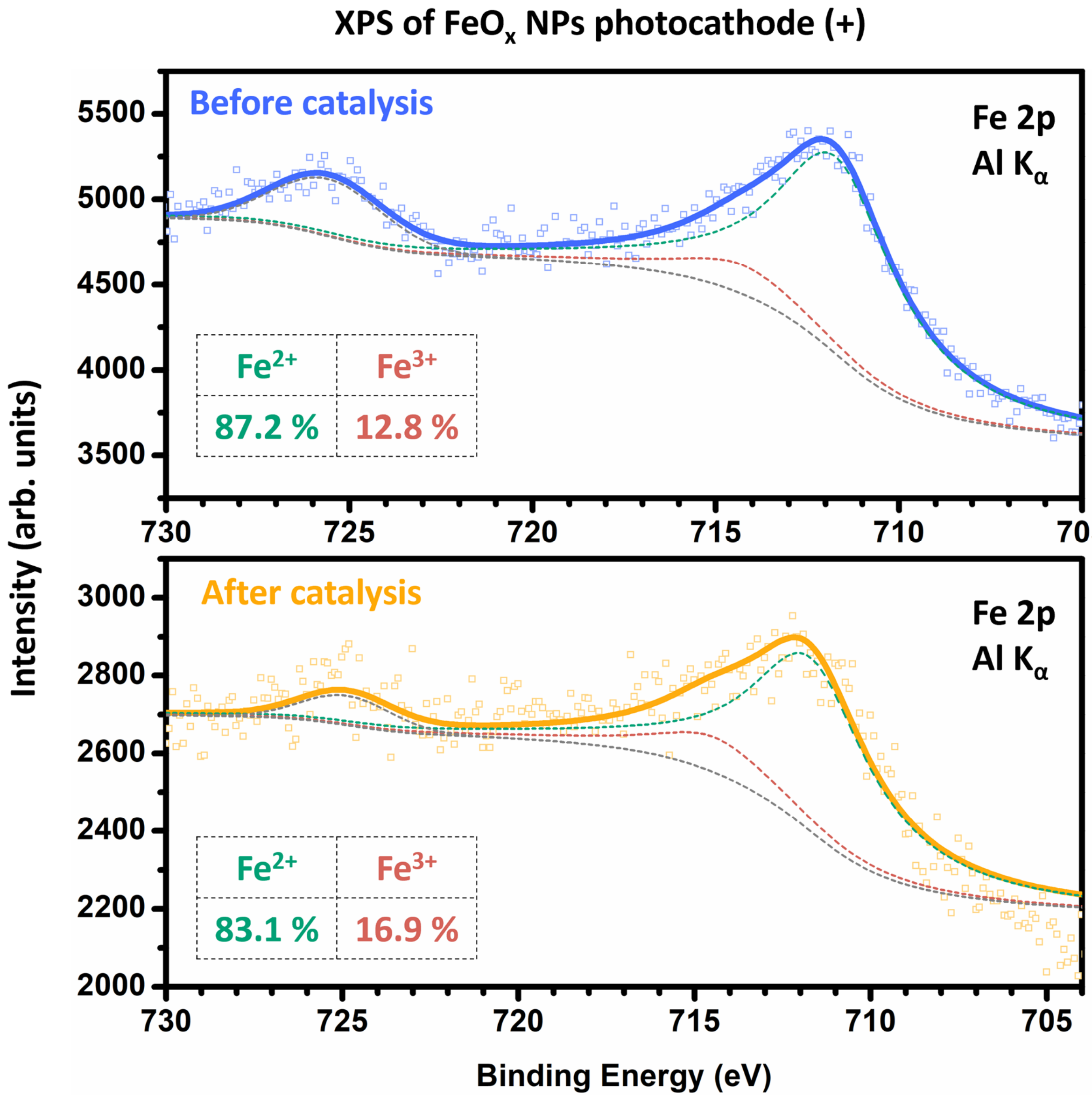

Figure S34: XPS of the Fe 2*p* core level region of a FeO_x NP photocathode (+), before and after catalysis; the percentages refer to the relative spectral intensities of the two components as deduced from the fit. Data are plotted as dots, the corresponding fits as continuous lines.

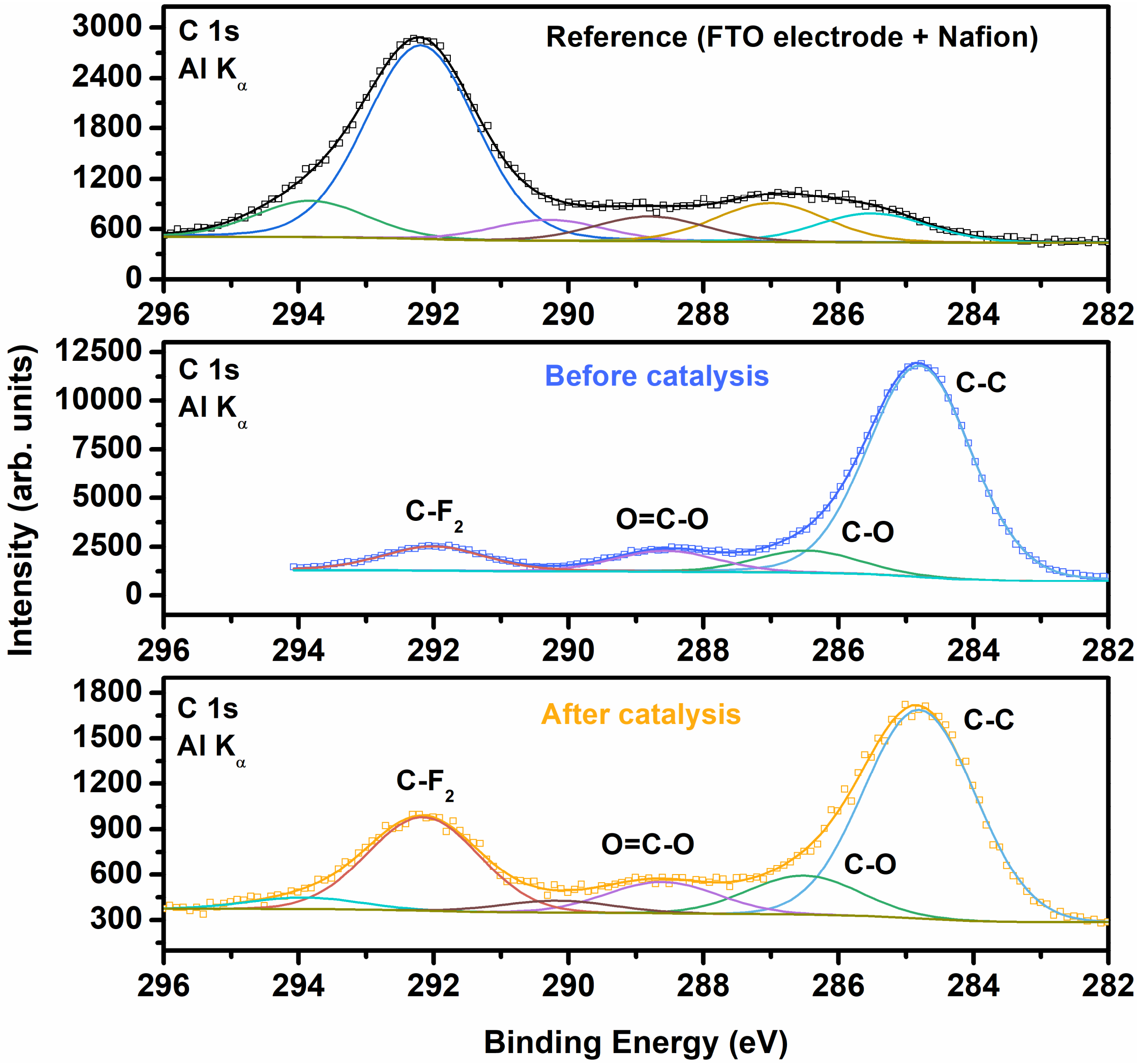

Figure S35: XPS spectra of the C 1*s* core level region of a FeO_x NP photocathode (+), before and after catalysis; for comparison the spectrum of the reference (Nafion coated on FTO glass electrode) is also shown. Data are plotted as dots, the corresponding fits as continuous lines.

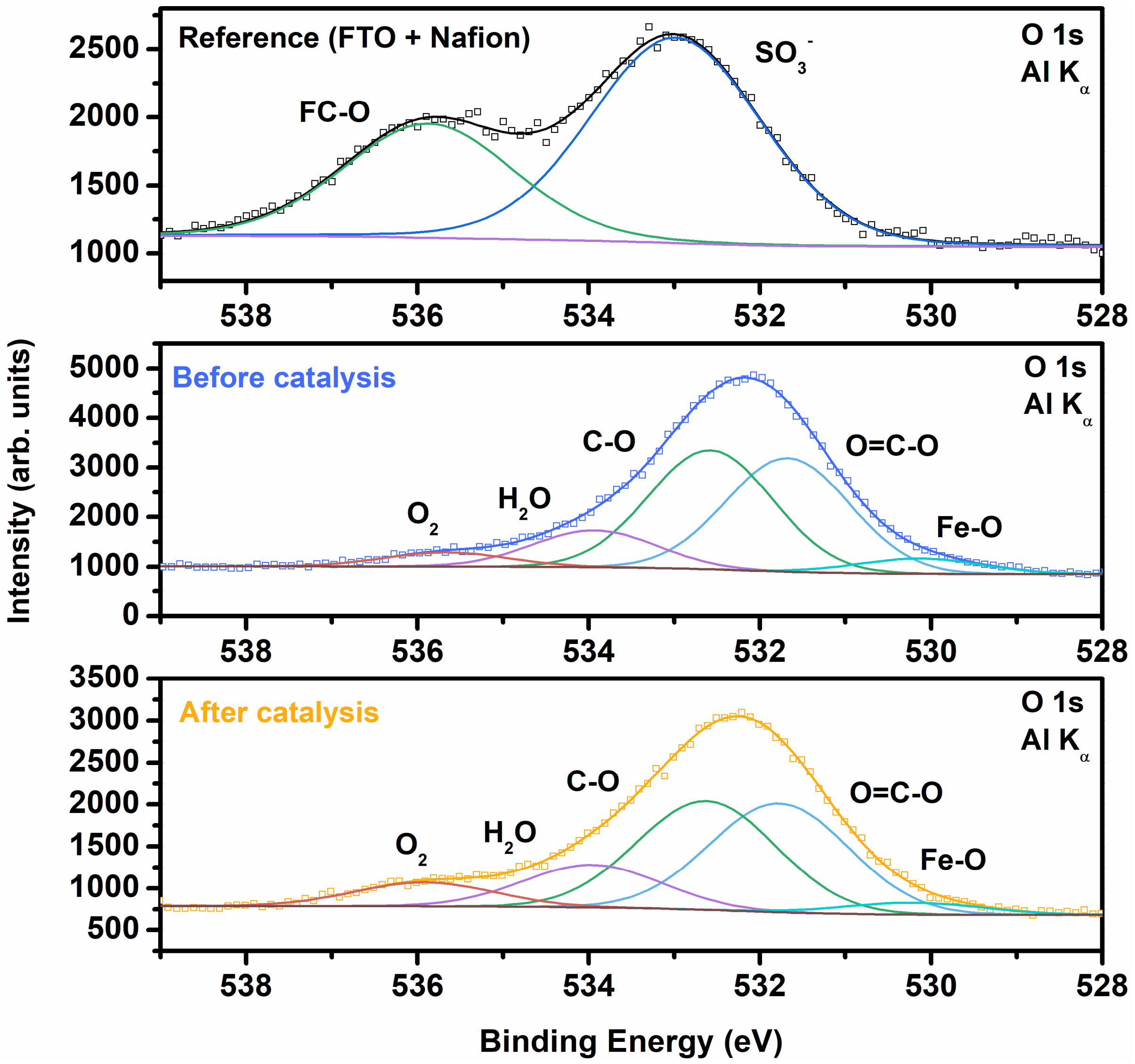

Figure S36: XPS spectra of the O 1s core level region of a FeO_x NPs photocathode (+), before and after catalysis; for comparison the reference (Nafion coated on FTO glass electrode) is also plotted; data are plotted as dots, the corresponding fits as continuous lines.

Table S5: Atomic percentages of the chemical species, before and after catalysis, as well as reference sample, as deduced from the XPS data. For the core level regions Fe 2*p*, C 1*s*, O 1*s* the relative spectral intensities of the components, as deduced from the fit of the XPS spectra, are depicted (top left table).

Elemental contribution	Before	After		Reference
Carbon	60.7 %	75.4 %		
Oxygen	37.1 %	23.0 %		
Iron	2.2 %	1.6 %		
**Fe 2*p* fit values**				
Fe^{2+}	87.2 %	83.1 %		
Fe^{3+}	12.8 %	16.9 %		
**C 1*s* fit values**				**Reference**
C-C/C-H	75.5 %	52.7 %	C-C	8.5 %
C-O	7.8 %	9.8 %	C-O	11.3 %
O-C=O	7.4 %	7.9 %	$C-SO_3$	7.2 %
C-F		3.0 %	C-F	6.1 %
$C-F_2$	9.3 %	23.7 %	$C-F_2$	56.4 %
$C-F_3$		2.9 %	$C-F_3$	10.6 %
**O 1*s* fit values**				
O-Fe	5.1 %	4.0 %		
O-C=O	38.0 %	35.8 %	O_3S	64.3 %
O-C	39.9 %	37.7 %	O-C	35.7 %
	12.3 %	14.4 %		
	4.7 %	8.1 %		

Binding energies of respective core level region	Before	After		Reference
**Fe 2*p***				
Fe^{2+}	711.7 eV	711.7eV		
Fe^{3+}	714.3 eV	714.4 eV		
**C 1*s***				**Reference**
C-C/C-H	284.8 eV	284.8 eV	C-C	285.5 eV
C-O	286.5 eV	286.5 eV	C-O	287.0 eV
O-C=O	288.6 eV	288.6 eV	$C-SO_3$	288.8 eV
C-F		290.2 eV	C-F	290.3 eV
$C-F_2$	292.0 eV	292.2 eV	$C-F_2$	292.2 eV
$C-F_3$		293.9 eV	$C-F_3$	293.8 eV
**O 1*s***				
O-Fe	530.1 eV	530.2 eV		
O-C=O	531.7 eV	531.8 eV		
O-C	532.6 eV	532.6 eV		
	533.9 eV	534.0 eV	O_3S	533.0 eV
	535.7 eV	535.9 eV	O-C	535.9 eV

Table S6: XPS Data on aging and stability of photoelectrodes upon storage. For the core level regions Fe 2*p*, C 1*s*, O 1*s* the relative spectral intensities of the components, as deduced from the fit of the XPS spectra, are depicted (top).

**Elemental contribution**	**Sample (January)**	**Sample (June)**
Carbon	60.7 %	75.6 %
Oxygen	37.1 %	22.7 %
Iron	2.2 %	1.7 %
**Fe 2*p* fit values**		
Fe^{2+}	87.2 %	88.5 %
Fe^{3+}	12.8 %	11.5 %
**C 1*s* fit values**		
C-C/C-H	75.5 %	62.3 %
C-O	7.8 %	8.9 %
O-C=O	7.4 %	12.2 %
C-F<br>$C-F_2$<br>$C-F_3$	9.3 %	16.6 %
**O 1*s* fit values**		
O-Fe	5.1 %	5.0 %
O-C=O	38.0 %	37.8 %
O-C	39.9 %	40.0 %
	12.3 %	11.0 %
	4.7 %	6.3 %

**Binding energies of respective core level region**	**Sample (January)**	**Sample (June)**
**Fe 2*p***		
Fe^{2+}	711.7 eV	711.7 eV
Fe^{3+}	714.3 eV	714.3 eV
**C 1*s***		
C-C/C-H	284.8 eV	284.8 eV
C-O	286.5 eV	286.6 eV
O-C=O	288.6 eV	288.7 eV
C-F<br>$C-F_2$<br>$C-F_3$	292.0 eV	291.9 eV
**O 1*s***		
O-Fe	530.1 eV	530.1 eV
O-C=O	531.7 eV	531.8 eV
O-C	532.6 eV	532.6 eV
	533.9 eV	534.0 eV
	535.7 eV	535.6 eV

13. References

1 L. N. Lameijer, S. Budzak, N. A. Simeth, M. J. Hansen, B. L. Feringa, D. Jacquemin and W. Szymanski, *Angew. Chem. Int. Ed.*, 2020, **59**, 21663–21670.

2 T. Freese, J. T. Meijer, M. B. Brands, G. Alachouzos, M. C. A. Stuart, R. Tarozo, D. Gerlach, J. Smits, P. Rudolf, J. N. H. Reek and B. L. Feringa, *EES Catalysis*, 2024, **2**, 262–275.

3 D. Prat, J. Hayler and A. Wells, *Green Chemistry*, 2014, **16**, 4546–4551.

4 D. Prat, A. Wells, J. Hayler, H. Sneddon, C. R. McElroy, S. Abou-Shehada and P. J. Dunn, *Green Chemistry*, 2015, **18**, 288–296.

5 L. Cseri, S. Kumar, P. Palchuber and G. Szekely, *ACS Sustain. Chem. Eng.*, 2023, **11**, 5696–5725.

6 T. Freese, R. Kat, S. D. Lanooij, T. C. Böllersen, C. M. De Roo, N. Elzinga, M. Beatty, B. Setz, R. R. Weber, I. Malta, T. B. Gandek, A. Krikken, P. Fodran, R. Pollice and M. M. Lerch, *ChemRxiv*, 2023, 1–76.

7 G. Wang, Y. Ling, D. A. Wheeler, K. E. N. George, K. Horsley, C. Heske, J. Z. Zhang and Y. Li, *Nano Lett*, 2011, **11**, 3503–3509.

8 C. Y. Lin, J. Y. Lin, C. C. Wan and T. C. Wei, *Electrochim. Acta*, 2011, **56**, 1941–1946.

9 D. F. Bruggeman, T. M. A. Bakker, S. Mathew and J. N. H. Reek, *Chem. – A European Journal*, 2021, **27**, 218–221.

10 C. C. L. McCrory, S. Jung, J. C. Peters and T. F. Jaramillo, *J. Am. Chem. Soc.*, 2013, **135**, 16977–16987.

11 L. Yu and Z. Ren, *Materials Today Physics*, 2020, **14**, 100253.

12 W. Zheng, *ACS Energy Lett.*, 2023, **8**, 1952–1958.

13 D. F. Bruggeman, A. A. H. Laporte, R. J. Detz, S. Mathew and J. N. H. Reek, *Angew. Chem. Int. Ed.*, 2022, **61**, e202200175.
